# New Information on the Cranial Anatomy of *Acrocanthosaurus atokensis* and Its Implications for the Phylogeny of Allosauroidea (Dinosauria: Theropoda)

**DOI:** 10.1371/journal.pone.0017932

**Published:** 2011-03-21

**Authors:** Drew R. Eddy, Julia A. Clarke

**Affiliations:** Department of Marine, Earth, and Atmospheric Sciences, North Carolina State University, Raleigh, North Carolina, United States of America; Raymond M. Alf Museum of Paleontology, United States of America

## Abstract

**Background:**

Allosauroidea has a contentious taxonomic and systematic history. Within this group of theropod dinosaurs, considerable debate has surrounded the phylogenetic position of the large-bodied allosauroid *Acrocanthosaurus atokensis* from the Lower Cretaceous Antlers Formation of North America. Several prior analyses recover *Acrocanthosaurus atokensis* as sister taxon to the smaller-bodied *Allosaurus fragilis* known from North America and Europe, and others nest *Acrocanthosaurus atokensis* within Carcharodontosauridae, a large-bodied group of allosauroids that attained a cosmopolitan distribution during the Early Cretaceous.

**Methodology/Principal Findings:**

Re-evaluation of a well-preserved skull of *Acrocanthosaurus atokensis* (NCSM 14345) provides new information regarding the palatal complex and inner surfaces of the skull and mandible. Previously inaccessible internal views and articular surfaces of nearly every element of the skull are described. Twenty-four new morphological characters are identified as variable in Allosauroidea, combined with 153 previously published characters, and evaluated for eighteen terminal taxa. Systematic analysis of this dataset recovers a single most parsimonious topology placing *Acrocanthosaurus atokensis* as a member of Allosauroidea, in agreement with several recent analyses that nest the taxon well within Carcharodontosauridae.

**Conclusions/Significance:**

A revised diagnosis of *Acrocanthosaurus atokensis* finds that the species is distinguished by four primary characters, including: presence of a knob on the lateral surangular shelf; enlarged posterior surangular foramen; supraoccipital protruding as a double-boss posterior to the nuchal crest; and pneumatic recess within the medial surface of the quadrate. Furthermore, the recovered phylogeny more closely agrees with the stratigraphic record than hypotheses that place *Acrocanthosaurus atokensis* as more closely related to *Allosaurus fragilis*. Fitch optimization of body size is also more consistent with the placement of *Acrocanthosaurus atokensis* within a clade of larger carcharodontosaurid taxa than with smaller-bodied taxa near the base of Allosauroidea. This placement of *Acrocanthosaurus atokensis* supports previous hypotheses of a global carcharodontosaurid radiation during the Early Cretaceous.

## Introduction

The most complete cranial specimen referred to the large-bodied theropod *Acrocanthosaurus atokensis*, NCSM 14345, comes from the Trinity Formation of North America (Aptian-Albian). The specimen was discovered along an incised creek bed southeast of Idabel, Oklahoma, with a nearly intact skull and associated, incomplete postcrania. Currie and Carpenter [1] originally described NCSM 14345, although the skull was incompletely prepared at that time. Sediment obscured the interior surfaces and, in some instances, entire views of cranial elements. Subsequent preparation of this specimen at the Black Hills Institute of Geological Research and the North Carolina Museum of Natural Sciences has allowed description and illustration of these previously undescribed cranial morphologies of *Acrocanthosaurus*. Here, we present a complete re-evaluation of the skull of *Acrocanthosaurus*, focusing on new data made available from NCSM 14345. From this morphological description, a suite of newly-recognized phylogenetic characters informative for allosauroid relationships is identified, and the phylogenetic position of *Acrocanthosaurus* is reassessed.

### Controversies concerning large theropods and “Carnosauria”

*Acrocanthosaurus atokensis* is among the largest non-avian theropod dinosaurs, which were historically thought to be more closely related to one another than to smaller-bodied forms. This notion led von Huene [2] to apply the name “Carnosauria” to what has subsequently been discovered to comprise a paraphyletic assemblage, including the supraspecific theropod taxa *Megalosaurus* Buckland 1824 [3], *Spinosaurus* Stromer 1915 [4], *Magnosaurus* von Huene 1932 [2], *Dryptosaurus* Marsh 1877 [5], and *Allosaurus* Marsh 1877 [5], and the rauisuchian *Teratosaurus* von Meyer 1861 [6]. This “carnosaurian” assemblage is now known to represent several independent origins of large size [7]–[11]. Although overall knowledge of non-avian theropod systematics has progressed substantially with discoveries of new species and specimens over the past 150 years, a detailed understanding of the evolutionary relationships of several theropod groups remains elusive [9]–[10], [12]–[14].

**Figure 1:**
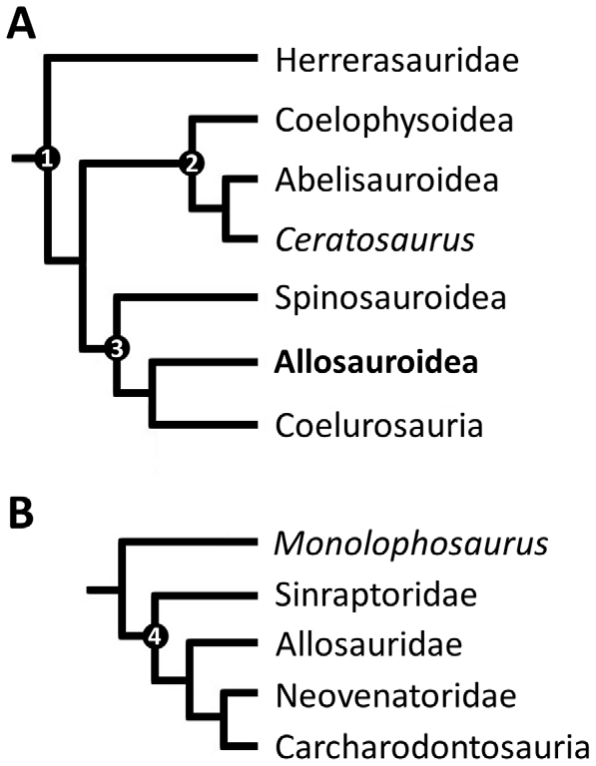
Generalized theropod phylogenies. Tree structures modified from Holtz *et al*. [12], O'Connor and Claessens [106], and the present analysis to illustrate the phylogenetic position of Allosauroidea (A) and relative placement of less-inclusive clades within Allosauroidea (B). **1**, Theropoda; **2**, Ceratosauria; **3**, Tetanurae; **4**, Allosauroidea.

Carnosauria von Huene 1920 [15] ( = Allosauroidea Currie and Zhao [16], see below) represents a particularly problematic theropod group that has historically fluctuated with respect to its included taxa and their interrelationships [1], [14], [17]–[25]. Gauthier's [14] early application of cladistic methodologies to estimate dinosaurian relationships led to his proposal that von Huene's name “Carnosauria” [15] be applied to a clade which excluded the basal theropods *Megalosaurus* and *Streptospondylus*, but included *Allosaurus*, *Acrocanthosaurus* Stovall and Langston 1950 [23], and several other theropod taxa. Additionally, his cladistic analysis suggested that Carnosauria be placed within Theropoda as the sister taxon to Coelurosauria [14], a hypothesis that has since been strongly supported (Figure 1) [1], [12], [13], [17], [24], [26]. However, Gauthier's proposed carnosaurian taxa [14] included several that are now recognized as coelurosaurs, such as *Tyrannosaurus rex* Osborn 1912 [27], *Daspletosaurus torosus* Russell 1970 [28], and *Albertosaurus sarcophagus* Osborn 1905 [29], as well as the abelisaurids *Indosuchus raptorius* von Huene and Matley 1933 [30] and *Indosaurus matleyi* von Huene and Matley 1933 [30]. As a result, Gauthier's suggested contents for Carnosauria were determined to be paraphyletic [9], [12], [17]; recognition of this paraphyly led to the practice of abandoning the name “Carnosauria” since it had become a “waste-basket” taxon for large-bodied theropods [8].

“Allosauroidea” was coined by Currie and Zhao [16] to refer to a clade including Allosauridae Marsh 1878 [31] and Sinraptoridae Currie and Zhao 1993 [16]. Sereno [8] proposed a similar stem-based definition for the name “Allosauroidea” that Holtz and Padian [18], [32] applied to the name “Carnosauria”: a clade including all taxa sharing a more recent common ancestor with *Allosaurus fragilis* than with *Passer domesticus* Linneaus 1758 [33]. In addition, Padian and Hutchinson [34] phylogenetically defined “Allosauroidea” prior to Sereno [8] as a node-based name for a clade including all descendants of the most recent common ancestor of *Allosaurus fragilis* and *Sinraptor dongi* Currie and Zhao 1993 [16]. The more restricted node-based name “Allosauroidea” and the stem-based name “Carnosauria” may both have utility in describing relationships among component taxa, although the presently known contents of these named clades may be identical. The present description and analysis follow the phylogenetic definitions for the names “Carnosauria” and “Allosauroidea” summarized in Padian *et al*. [32], but prefer to employ “Allosauroidea” in place of “Carnosauria” to maintain congruence with previous work on allosauroids.

### Taxonomic and phylogenetic history of Allosauroidea

Significant new specimens have illuminated the diversity within Allosauroidea during the past fifteen years [1], [20], [25], [35]–[37]. A consensus concerning the relationships of allosauroid taxa was problematic for some time [1], [9], [12]–[13], [17], [19]–[21], [26], [36], [38]–[41], but recent phylogenetic work has made substantial progress towards the resolution of the group [10], [22], [25], [42]. Within Allosauroidea, four subclades have been recognized and are regularly differentiated by phylogenetic analyses: Allosauridae, Sinraptoridae, Carcharodontosauridae Stromer 1931 [43], and Neovenatoridae Benson, Carrano, and Brusatte 2009 [42] (Figure 1). The name “Allosauridae” has been applied to the clade including all taxa more closely related to *Allosaurus fragilis* than to *Carcharodontosaurus saharicus* Depéret and Savornin 1927 [44] and *Sinraptor dongi* [32], [34], but presently comprises only the taxon *Allosaurus*. “Sinraptoridae” defines the clade including all taxa more closely related to *Sinraptor dongi* than to *Allosaurus fragilis* and *Carcharodontosaurus saharicus* [34], and frequently comprises the taxa *Sinraptor* and *Yangchuanosaurus* Dong, Chang, Li, and Zhou 1978 [45], although recent analyses [10], [25] found Sinraptoridae to also include *Lourinhanosaurus* Mateus 1998 [46] and *Metriacanthosaurus* Walker 1964 [47].

Stromer [43] coined the name “Carcharodontosauridae”, and Sereno [8] later gave it a phylogenetic definition as a stem-based name for a clade that includes all taxa more closely related to *Carcharodontosaurus saharicus* than to *Sinraptor dongi*, *Allosaurus fragilis*, or *Passer domesticus*. Discovery and subsequent phylogenetic placement of new allosauroid taxa (*i.e.*, *Australovenator wintonensis* Hocknull, White, Tischler, Cook, Calleja, Sloan, and Elliott 2009 [48]; *Concavenator corcovatus* Ortega, Escaso, and Sanz 2010 [25]; *Eocarcharia dinops* Sereno and Brusatte 2008 [49]; *Mapusaurus roseae* Coria and Currie 2006 [36]; *Shaochilong maortuensis* Brusatte, Benson, Chure, Xu, Sullivan, and Hone 2009 [37], [50]; and *Tyrannotitan chubutensis* Novas, De Valais, Vickers-Rick, and Rich 2005 [39]) has prompted the recognition of “Carcharodontosaurinae”, defined by Brusatte and Sereno [22] as a node-based name for the least-inclusive clade containing *Carcharodontosaurus saharicus* and *Giganotosaurus carolinii* Coria and Salgado 1995 [35]. Carcharodontosaurinae is consistently recovered as containing the derived carcharodontosaurid taxa *Carcharodontosaurus*, *Giganotosaurus*, and *Mapusaurus* [10], [13], [22], [36]–[37], [42].

Substantial taxonomic and phylogenetic modifications to Allosauroidea were proposed by Benson *et al.* [42] in their assessment of the relationships of several enigmatic Cretaceous theropod taxa with proposed allosauroid affinities. Although several of these taxa are known from largely incomplete specimens with little cranial material (*e.g.*, *Aerosteon riocoloradensis* Sereno, Martinez, Wilson, Varricchio, Alcober, and Larsson 2008 [51], *Australovenator wintonensis* [48], *Megaraptor namunhuaiquii* Novas 1998 [52], *Fukuiraptor kitadaniensis* Azuma and Currie 2000 [53], *Chilantaisaurus tashuikouensis* Hu 1964 [50]), a phylogenetic analysis combined with substantial postcranial data recovered within Allosauroidea the separate monophyletic group “Neovenatoridae” with *Neovenator salerii* Hutt, Martill, and Barker 1996 [54] as the most basal member [42]. Benson *et al*. [42] defined Neovenatoridae as the most inclusive clade containing *Neovenator salerii*, but not *Carcharodontosaurus saharicus*, *Allosaurus fragilis*, or *Sinraptor dongi*. Neovenatoridae is found to comprise the taxa *Aerosteon*, *Australovenator*, *Chilantaisaurus*, *Fukuiraptor*, and *Megaraptor* [10], [25]. The recovery of “Neovenatoridae” as the sister taxon to Carcharodontosauridae further prompted the formation of the name “Carcharodontosauria” Benson, Carrano, and Brusatte 2009 [42] to describe the most inclusive clade comprising *Carcharodontosaurus saharicus* and *Neovenator salerii*, but not *Allosaurus fragilis* or *Sinraptor dongi*. Amendment of the name “Carcharodontosauridae” was also proposed in order to change its phylogenetic definition to the most inclusive clade comprising *Carcharodontosaurus saharicus*, but not *Neovenator salerii*, *Allosaurus fragilis*, or *Sinraptor dongi* [42], and this distinction between Carcharodontosauridae and Carcharodontosauria is followed herein.

*Acrocanthosaurus atokensis* is the first-named and only species currently recognized as valid in the genus *Acrocanthosaurus*. The genus name stems from the Latin for “high-spined lizard”, as specimens referred to that taxon exhibit exceptionally tall neural spines along cervical and dorsal vertebrae [1], [21], [23]. The species name references Atoka County in southeastern Oklahoma, from which the holotype and paratype specimens were recovered. Reconstructions of the taxon upon its initial discovery were limited by a paucity of cranial material, although *Acrocanthosaurus atokensis* was suggested to be an intermediate form between allosauroids and tyrannosaurids [23]. Subsequent study suggested *Acrocanthosaurus atokensis* to be a tyrannosaurid due to similarities in size [55]. Conflicting phylogenetic placements of *Acrocanthosaurus atokensis* once prevented a consensus on relationships within Allosauroidea [22]. Previous analyses recovered this taxon alternatively as closely related to the smaller-bodied taxon *Allosaurus fragilis* from North America and Europe [1], [13], [36], [39], [56], or placed within Carcharodontosauridae [10], [12], [17], [19]–[22], [25], [42], [49]. However, recent phylogenetic work has shown consistent support for *Acrocanthosaurus atokensis* as a carcharodontosaurid [10], [22], [25], [37], [42].

### Institutional abbreviations

AMNH, American Museum of Natural History, New York, NY, USA; CMNH, Carnegie Museum of Natural History, Pittsburgh, PA, USA; CV, Municipal Museum of Chongqing, Chongqing, People's Republic of China; FWMSH, Forth Worth Museum of Science and History, Fort Worth, TX, USA; IVPP, Institute of Vertebrate Paleontology and Paleoanthropology, Beijing, People's Republic of China; MCF-PVPH, Museo Carmen Funes, Paleontología de Vertebrados, Plaza Huincul, Neuquén, Argentina; MIWG, Museum of Isle of Wight Geology, Sandown, U.K.; MNN, Musée National du Niger, Niamey, Republic of Niger; MPEF-PV, Museo Paleontológico “Egidio Feruglio”, Trelew, Argentina; MUCPv-CH, Museo de la Universidad Nacional del Comahue, El Chocón Collection, Neuquén, Argentina; NCSM, North Carolina Museum of Natural Sciences, Raleigh, NC, USA; OMNH, Sam Noble Oklahoma Museum of Natural History, Norman, OK, USA; PVL, Instituto Miguel Lillo, Tucumán, Argentina; PVSJ, Instituto y Museo de Ciencias Naturales, San Juan, Argentina; SGM, Ministére de l'Energie et des Mines, Rabat, Morocco; SMU, Southern Methodist University, Dallas, TX, USA; USNM, United States National Museum, Smithsonian Institution, Washington D.C., USA; UUVP, Utah Museum of Natural History, Salt Lake City, UT, USA.

## Methods

### Preparation and Imaging

**Figure 2:**
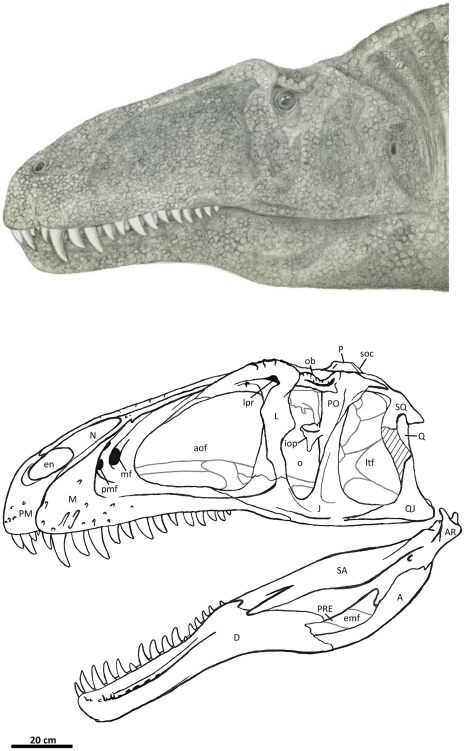
Flesh reconstruction and line drawing of the skull of *Acrocanthosaurus atokensis* (NCSM 14345) in left lateral view. Hatched lines represent missing bone. **A**, angular; **aof**, antorbital fenestra; **AR**, articular; **D**, dentary; **emf**, external mandibular fenestra; **iop**, intraorbital process of postorbital; **J**, jugal; **L**, lacrimal; **lpr**, lacrimal pneumatic recess; **ltf**, lateral temporal fenestra; **M**, maxilla; **mf**, maxillary fenestra; **N**, nasal; **o**, orbit; **ob**, orbital boss of postorbital; **P**, parietal; **PM**, premaxilla; **pmf**, promaxillary fenestra; **PO**, postorbital; **PRE**, prearticular; **Q**, quadrate; **QJ**, quadratojugal; **SA**, surangular; **soc**, supraoccipital; **SQ**, squamosal.

**Figure 3 pone-0017932-g003:**
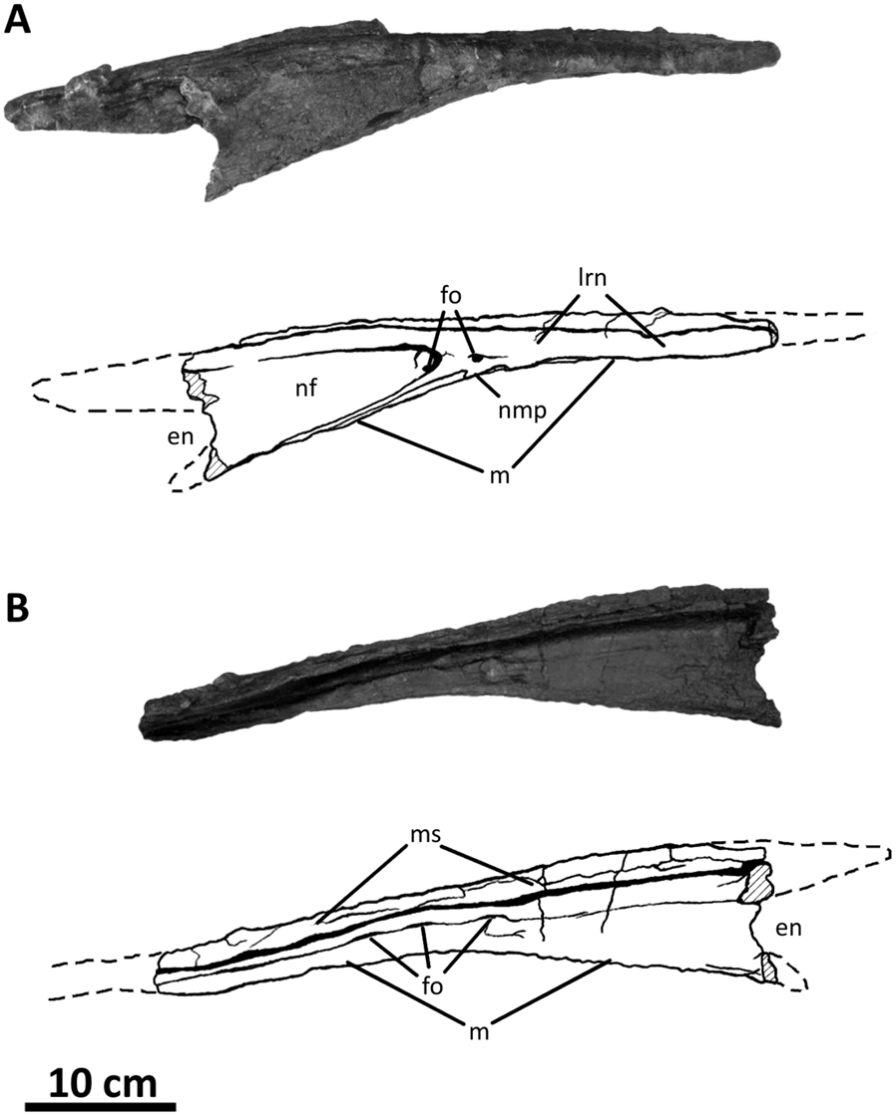
Left nasal of *Acrocanthosaurus atokensis* (NCSM 14345). Nasal in (A) lateral and (B) medial views. Hatched lines represent broken surfaces; dashed lines represent material not in figure. **en**, external naris; **fo**, foramina; **lrn**, lateral ridge of nasal; **m**, maxillary contact; **ms**, medial symphysis; **nf**, narial fossa; **nmp**, naso-maxillary process.

**Figure 4 pone-0017932-g004:**
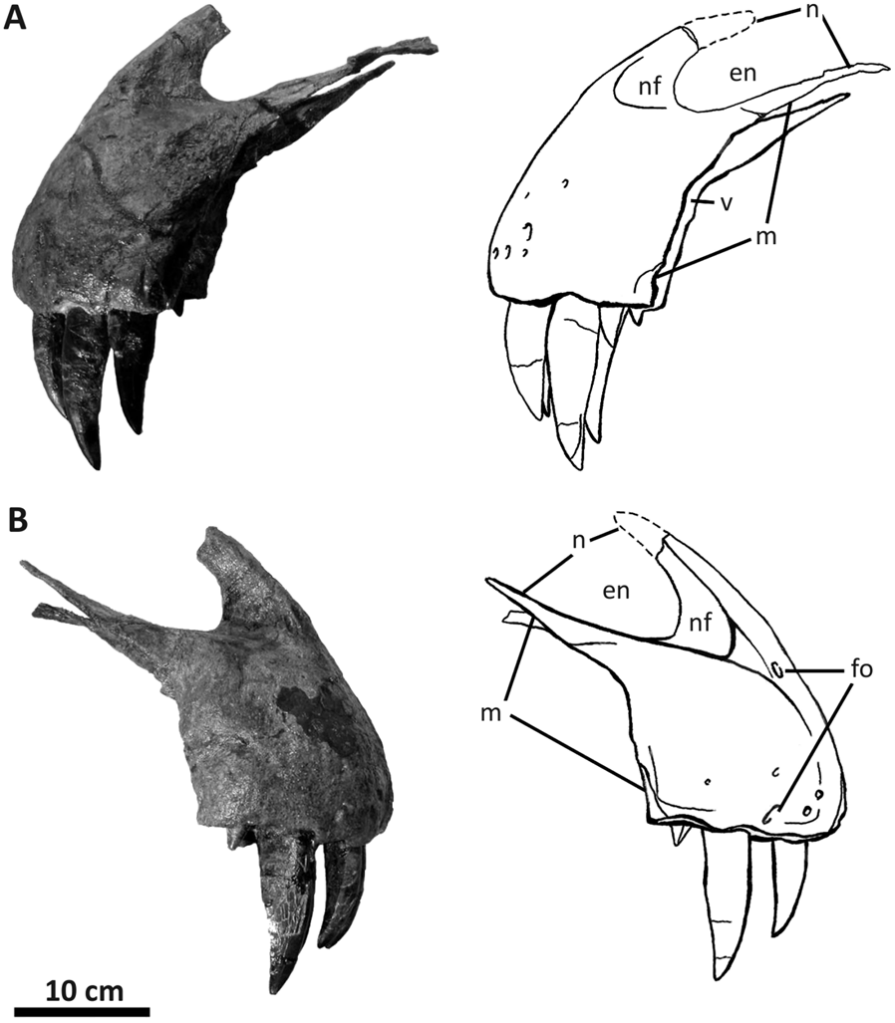
Premaxillae of *Acrocanthosaurus atokensis* (NCSM 14345). Premaxillae in (A) left lateral and (B) right lateral views. Dashed lines represent material not in figure. **en**, external naris; **fo**, foramina; **m**, maxillary contact; **n**, nasal contact; **nf**, narial fossa; **v**, vomeral contact.

The skull of NCSM 14345 is currently displayed at the North Carolina Museum of Natural Sciences in Raleigh, North Carolina. Most cranial elements are adhered together to strengthen the structure of the mounted skull. Therefore, line drawings (Figures 2–11, 19–32) were completed using cast material molded before the assembly of the skull. These carefully prepared study casts allowed the interior and articular surfaces of nearly all cranial elements to be fully described and illustrated. Line drawings made from cast material were compared to cranial elements as currently mounted to correct for features not reproduced by the casts (*e.g.*, small fossae, foramina). Photographs were taken of original material (Figures 3A, 4, 6–9, 10A, 10C, 11, 20, 21, 25, 26, 29, 30, 31, 45A) and casts (Figures 3B, 10B). X-ray computed tomographic (CT) scans of the braincase (Figures 12–16) were generated from data gathered at the North Carolina State University College of Veterinary Medicine and edited in OsiriX [57]. The scan is reposited at the North Carolina Museum of Natural Sciences. The dataset consists of 730 1.0 mm-thick slices with an inter-slice spacing of 0.79 mm. From these braincase slices, a digital endocast (Figures 17, 18) was constructed in Avizo v.5.0.1 [58] using a combination of manual and automatic segmentation. Measurements described in the text are from the left side of the skull and provided in Table 1.

**Figure 5 pone-0017932-g005:**
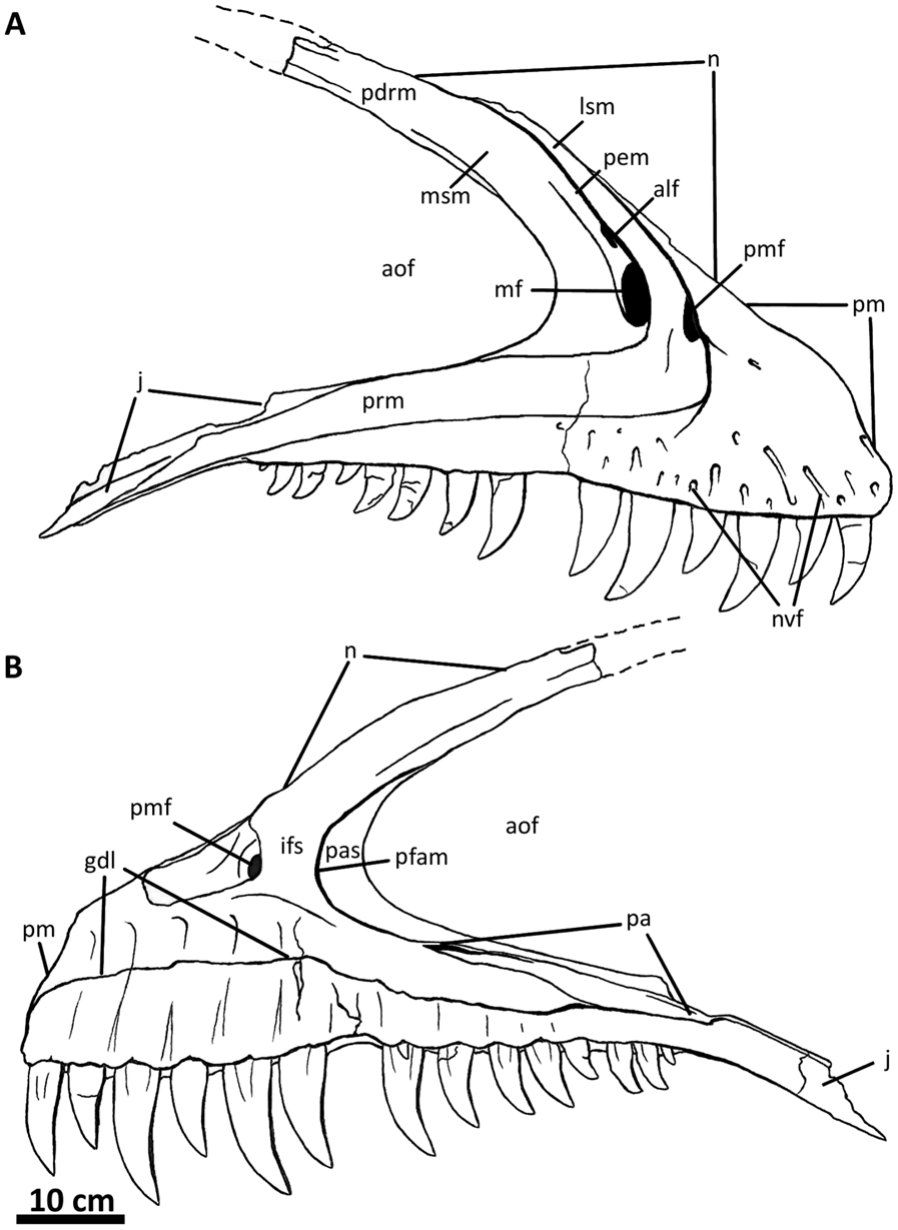
Right maxilla of *Acrocanthosaurus atokensis* (NCSM 14345). Maxilla in (A) lateral and (B) medial views. Dashed lines represent material not in figure. **aof**, antorbital fenestra; **alf**, accessory lateral fenestra of the maxilla; **gdl**, groove for dental lamina; **ifs**; interfenestral strut; **j**, jugal contact; **lsm**, lateral shelf; **mf**, maxillary fenestra; **n**, nasal contact; **nvf**, neurovascular foramina; **pas**, postantral strut; **pdrm**, posterodorsal ramus of the maxilla; **pem**, pneumatic excavation of the posterodorsal ramus; **pfam**, posterior fenestra of the maxilla; **pm**, premaxillary contact; **pmf**, promaxillary fenestra; **prm**; posterior ramus of the maxilla.

**Figure 6 pone-0017932-g006:**
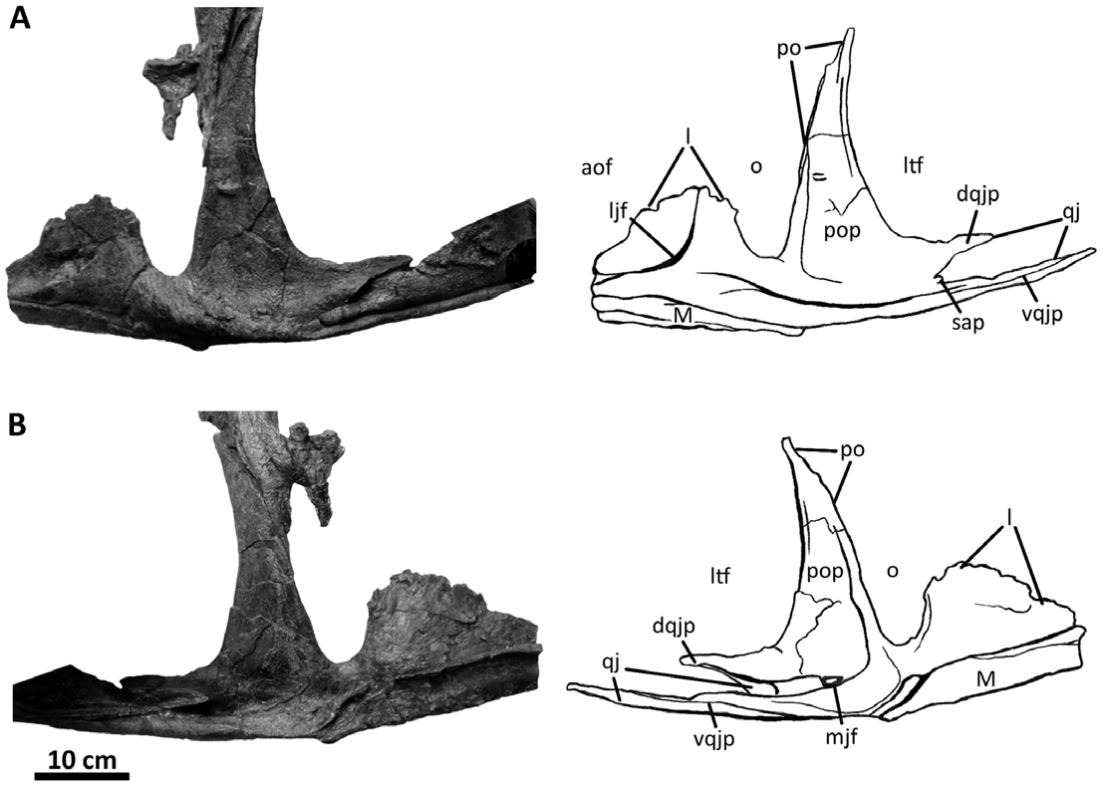
Left jugal of *Acrocanthosaurus atokensis* (NCSM 14345). Jugal in (A) lateral and (B) medial views. Dashed lines represent material not in figure. **aof**, antorbital fenestra; **dqjp**, dorsal quadratojugal prong; **l**, lacrimal contact; **ljf**, lateral jugal foramen; **ltf**, lateral temporal fenestra; **M**, maxilla; **mjf**, medial jugal foramen; **o**, orbit; **po**, postorbital contact; **pop**, postorbital process of jugal; **qj**, quadratojugal contact; **sap**, small accessory prong; **vqjp**, ventral quadratojugal prong.

**Figure 7 pone-0017932-g007:**
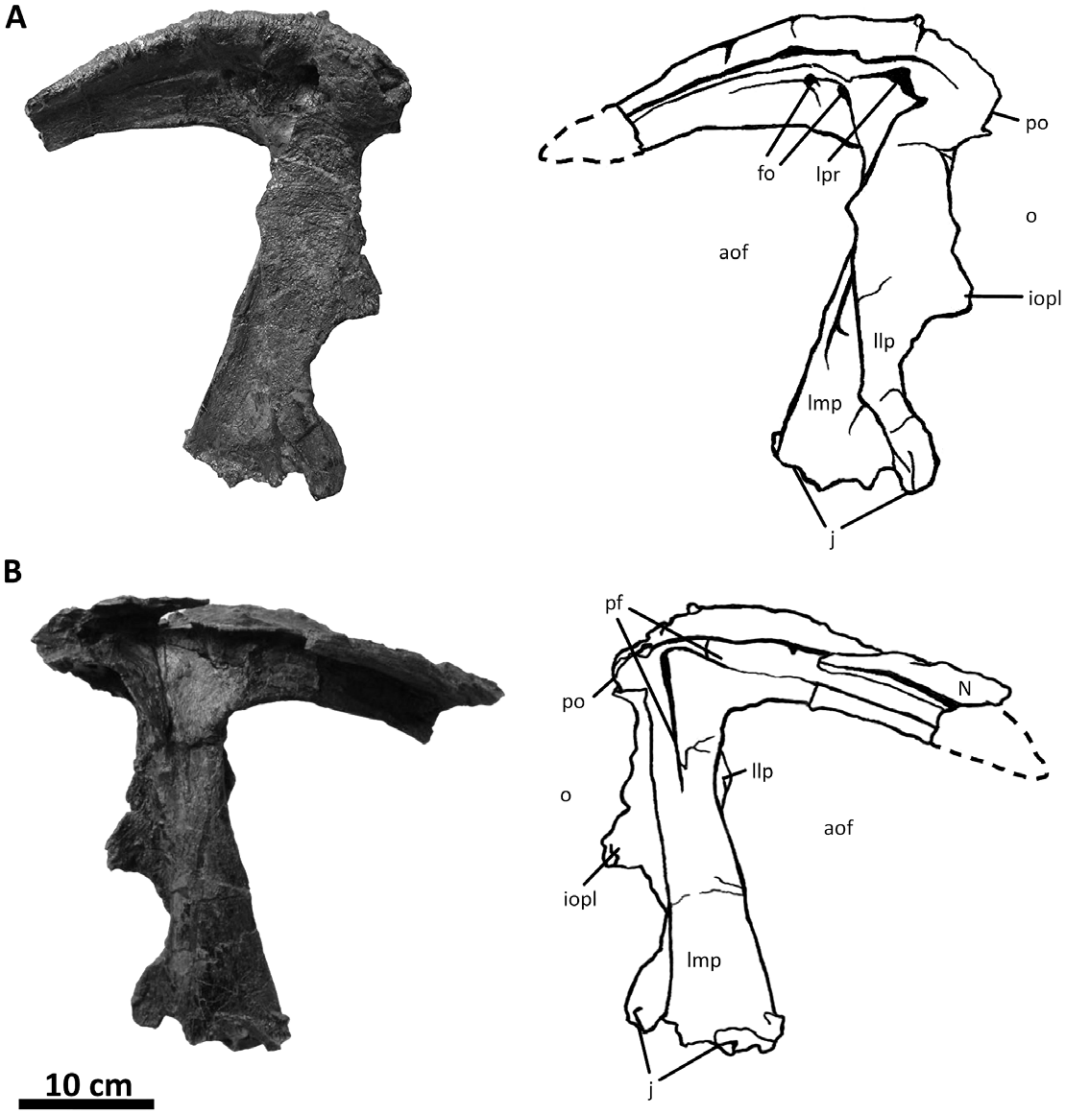
Left lacrimal of *Acrocanthosaurus atokensis* (NCSM 14345). Lacrimal in (A) lateral and (B) medial views. Dashed lines represent material not in figure. **aof**, antorbital fenestra; **fo**, foramina; **iopl**, intraorbital process of lacrimal; **j**, jugal contact; **llp**, lacrimal lateral plate; **lmp**, lacrimal medial plate; **lpr**, lacrimal pneumatic recess; **N**, nasal; **o**, orbit; **pf**, prefrontal contact; **po**, postorbital contact.

**Figure 8 pone-0017932-g008:**
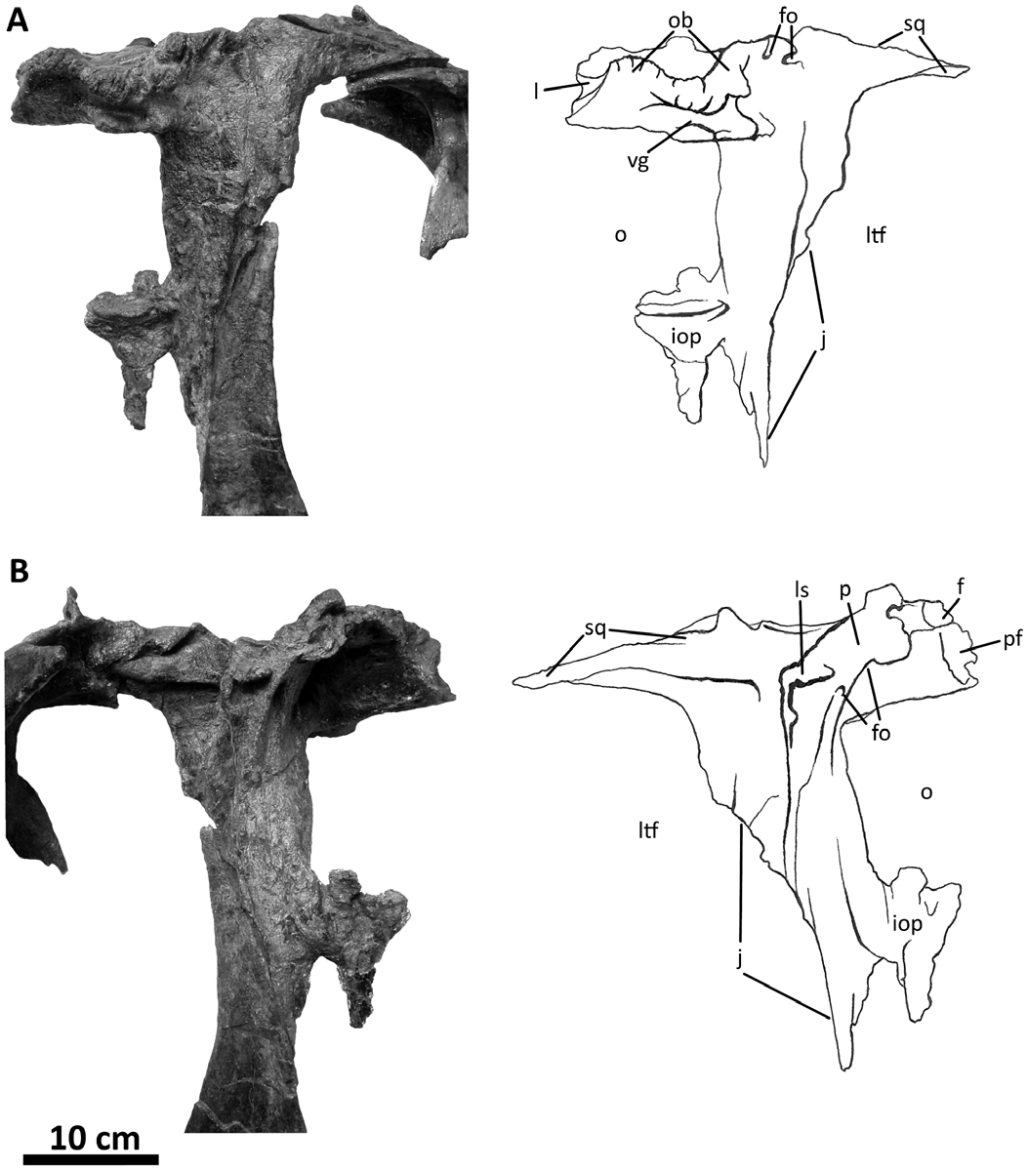
Left postorbital of *Acrocanthosaurus atokensis* (NCSM 14345). Postorbital in (A) lateral and (B) medial views. **f**, frontal contact; **fo**, foramina; **iop**, intraorbital process of postorbital; **j**, jugal contact; **l**, lacrimal contact; **ls**, laterosphenoid contact; **ltf**, lateral temporal fenestra; **o**, orbit; **ob**, orbital boss of postorbital; **p**, parietal contact; **pf**, prefrontal contact; **sq**, squamosal contact; **vg**, vascular groove.

**Figure 9 pone-0017932-g009:**
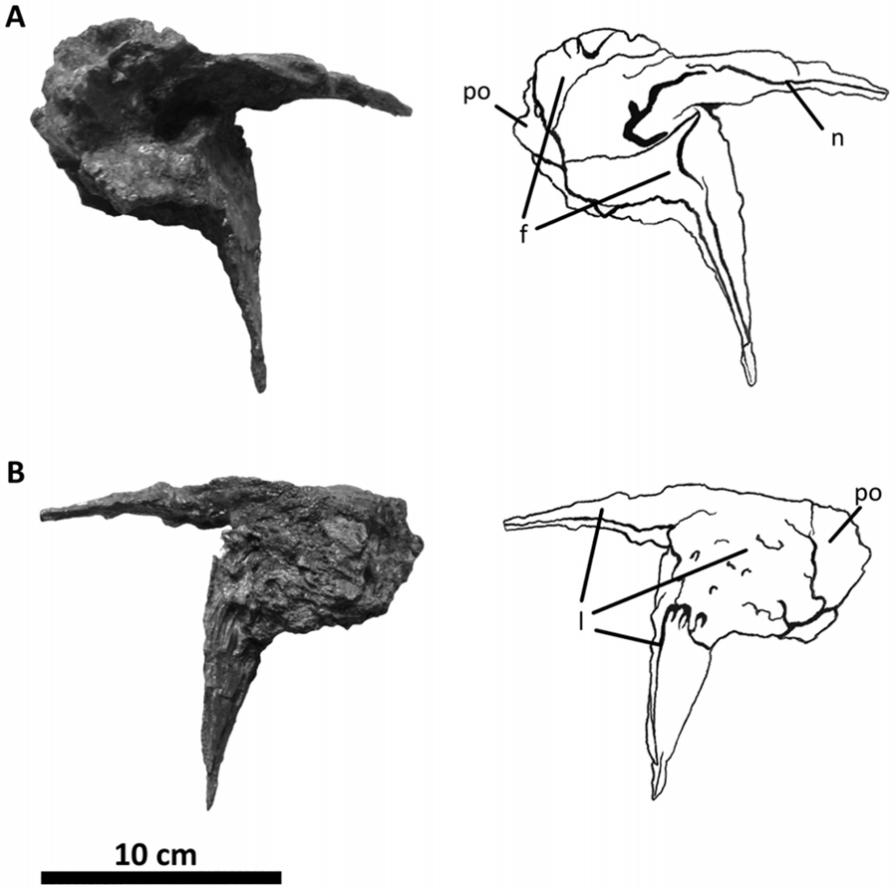
Left prefrontal of *Acrocanthosaurus atokensis* (NCSM 14345). Prefrontal in (A) medial and (B) lateral views. **f**, frontal contact; **l**, lacrimal contact; **n**, nasal contact; **po**, postorbital contact.

**Figure 10 pone-0017932-g010:**
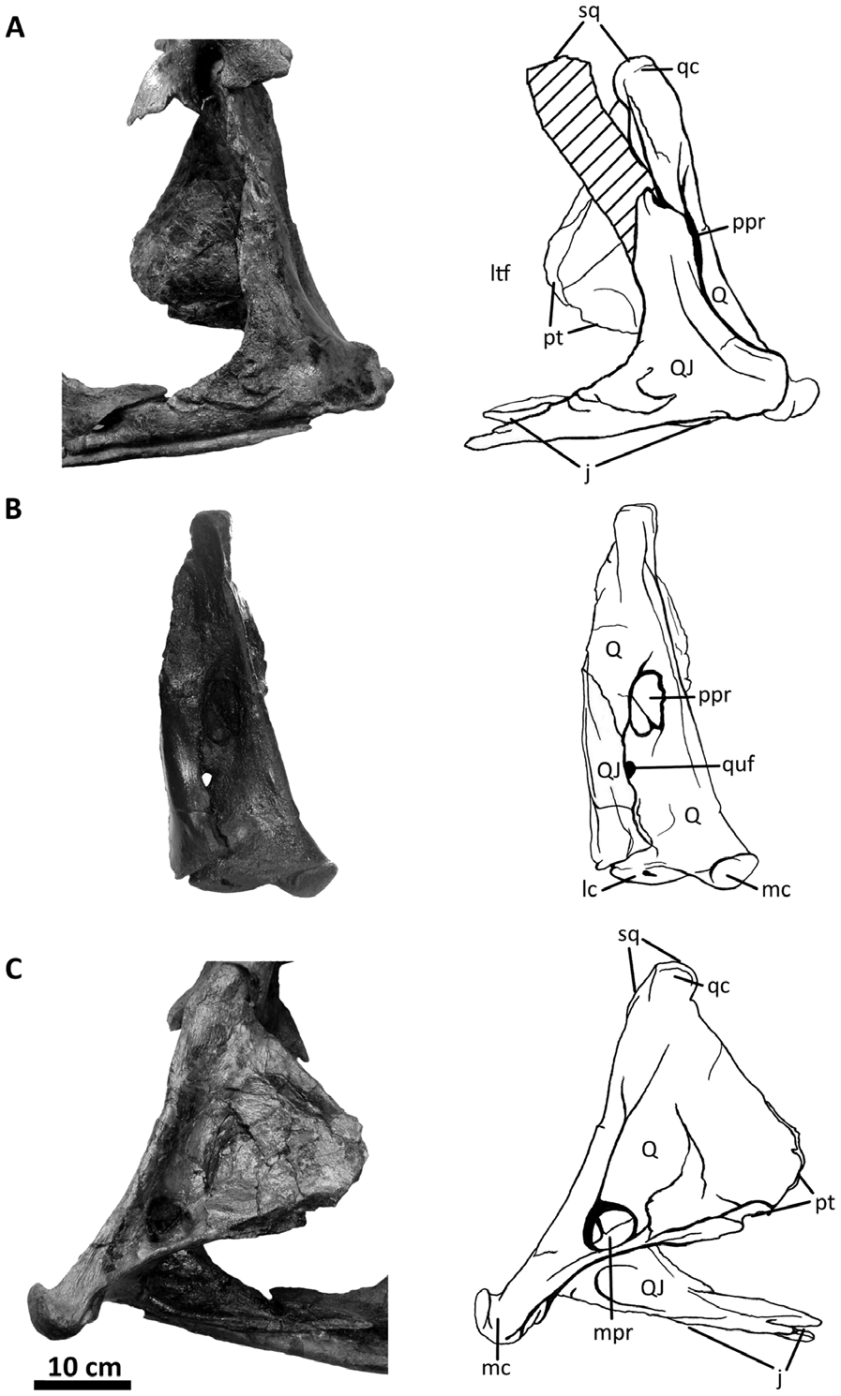
Left quadratojugal and quadrate of *Acrocanthosaurus atokensis* (NCSM 14345). Quadratojugal and quadrate in (A) lateral, (B) posterior, and (C) medial views. Hatched lines represent missing material. **j**, jugal contact; **lc**, lateral condyle of quadrate; **ltf**, lateral temporal fenestra; **mc**, medial condyle of quadrate; **mpr**, medial pneumatic recess of quadrate; **ppr**, posterior pneumatic recess of quadrate; **pt**, pterygoid contact; **Q**, quadrate; **qc**, quadrate cotylus; **QJ**, quadratojugal; **quf**, quadrate foramen; **sq**, squamosal contact.

**Figure 11:**
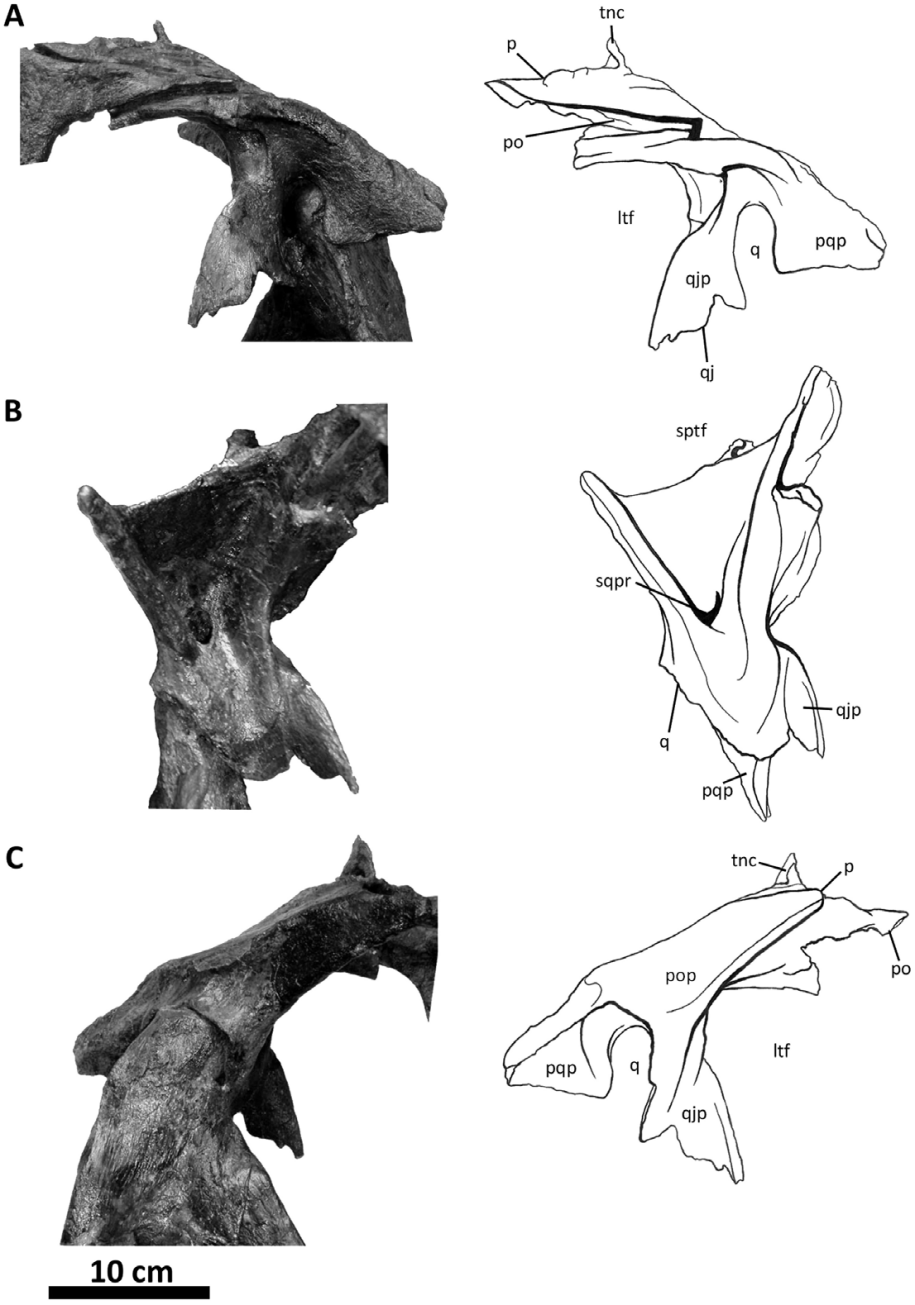
Left squamosal of *Acrocanthosaurus atokensis* (NCSM 14345). Squamosal in (A) lateral, (B) ventral, and (C) medial views. **ltf**, lateral temporal fenestra; **p**, parietal contact; **po**, postorbital contact; **pop**, contact with paroccipital process; **pqp**; postcotyloid process of squamosal; **q**, quadrate contact; **qj**, quadratojugal contact; **qjp**, quadratojugal process of squamosal; **sptf**, supratemporal fossa; **sqpr**, squamosal pneumatic recess (foramen); **tnc**, transverse nuchal crest.

**Figure 12:**
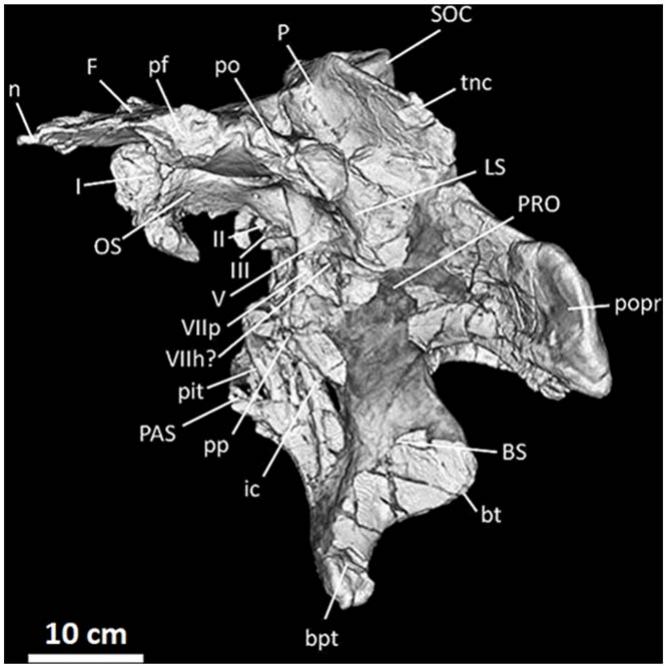
Frontals, parietals, and braincase of *Acrocanthosaurus atokensis* (NCSM 14345) in left lateral view. Reconstructed from CT scan data. **bpt**, basipterygoid process; **bt**, basal tubera; **BS**, basisphenoid; **F**, frontal; **I**, olfactory nerve exit; **ic**, internal carotid artery entrance; **II**, optic nerve exit; **III**, oculomotor nerve exit; **LS**, laterosphenoid; **n**, nasal contact; **OS**, orbitosphenoid; **P**, parietal; **PAS**, parasphenoid; **pf**, prefrontal contact; **pit**, pituitary fossa; **po**, postorbital contact; **popr**; paroccipital process; **pp**, preotic pendant; **PRO**, prootic; **SOC**, supraoccipital; **tnc**, transverse nuchal crest; **V**, trigeminal nerve exit; **VIIh**, hyomandibular branch of facial nerve exit; **VIIp**, palatine branch of facial nerve exit.

**Figure 13 pone-0017932-g013:**
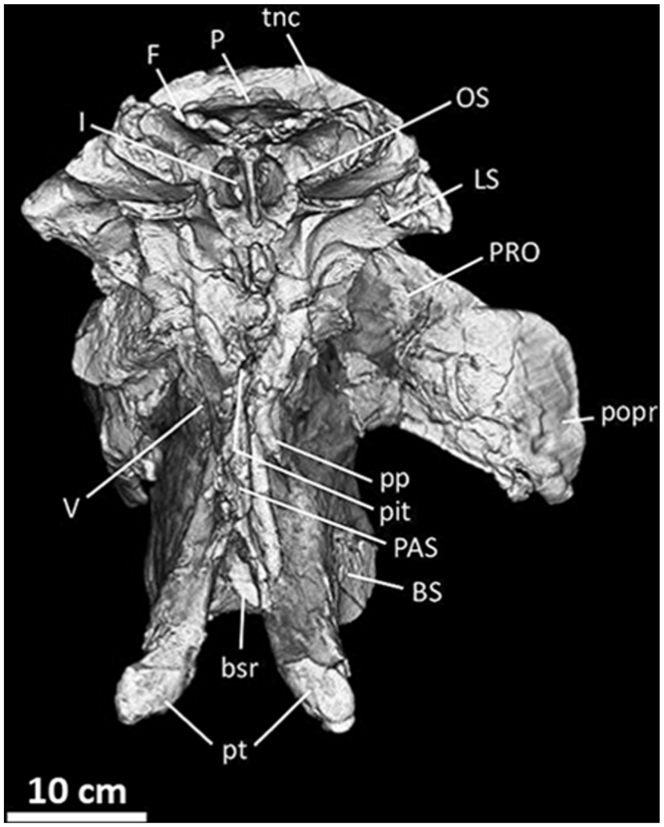
Frontals, parietals, and braincase of *Acrocanthosaurus atokensis* (NCSM 14345) in anterior view. Reconstructed from CT scan data. **BS**, basisphenoid; **bsr**, basisphenoid recess; **F**, frontal; **I**, olfactory nerve exit; **LS**, laterosphenoid; **OS**, orbitosphenoid; **P**, parietal; **PAS**, parasphenoid; **pit**, pituitary fossa; **popr**; paroccipital process; **pp**, preotic pendant; **PRO**, prootic; **pt**, pterygoid contact; **tnc**, transverse nuchal crest; **V**, trigeminal nerve exit.

**Figure 14 pone-0017932-g014:**
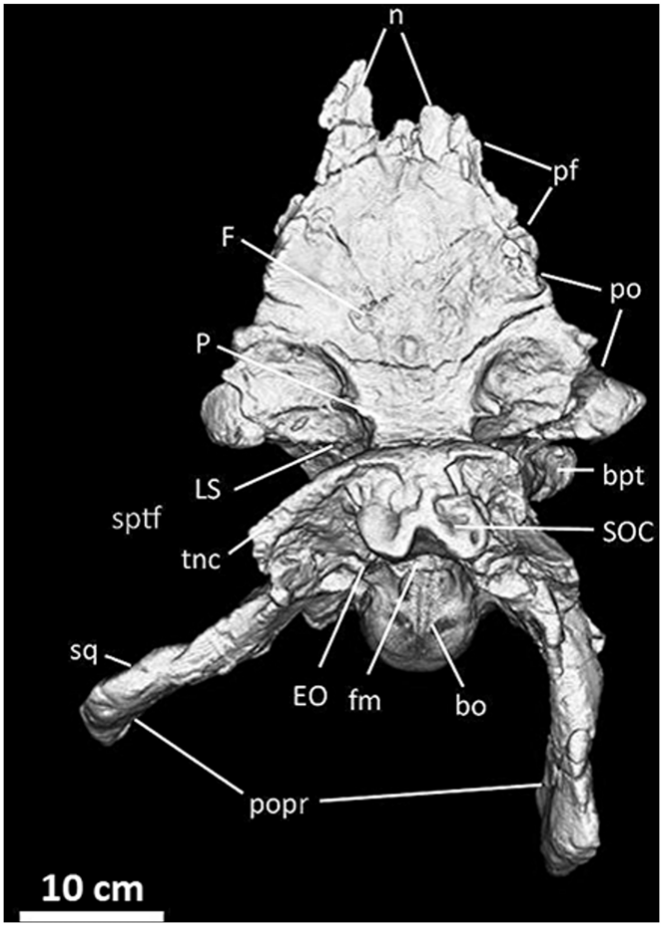
Frontals, parietals, and braincase of *Acrocanthosaurus atokensis* (NCSM 14345) in dorsal view. Reconstructed from CT scan data. **bo**, basioccipital; **bpt**, basipterygoid process; **EO**, exoccipital; **F**, frontal; **fm**, foramen magnum; **LS**, laterosphenoid; **n**, nasal contact; **P**, parietal; **pf**, prefrontal contact; **po**, postorbital contact; **popr**; paroccipital processes; **SOC**, supraoccipital; **sptf**, supratemporal fossa; **sq**, squamosal contact; **tnc**, transverse nuchal crest.

**Figure 15 pone-0017932-g015:**
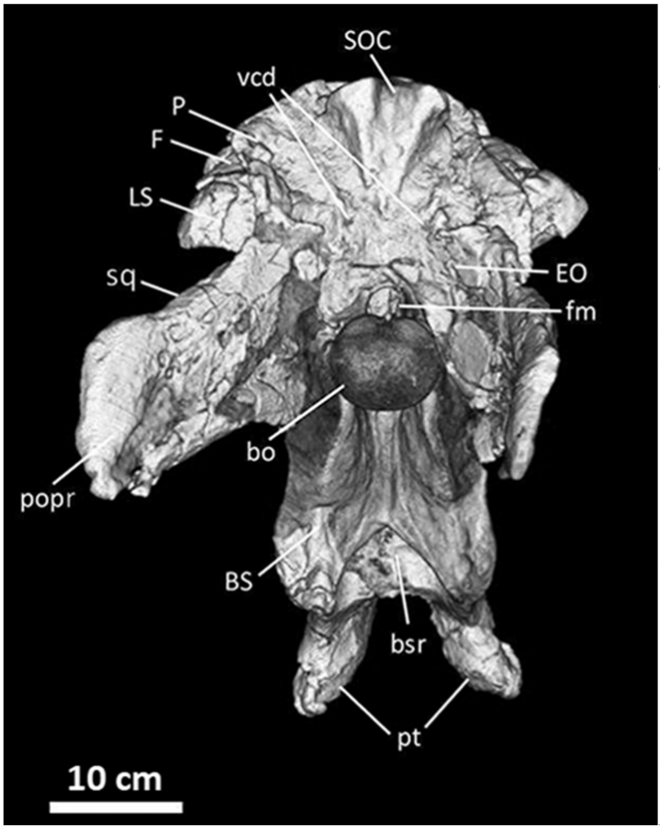
Frontals, parietals, and braincase of *Acrocanthosaurus atokensis* (NCSM 14345) in posterior view. Reconstructed from CT scan data. **bo**, basioccipital; **BS**, basisphenoid; **bsr**, basisphenoid recess; **EO**, exoccipital; **F**, frontal; **fm**, foramen magnum; **LS**, laterosphenoid; **P**, parietal; **popr**; paroccipital process; **pt**, pterygoid contact; **SOC**, supraoccipital; **sq**, squamosal contact; **vcd**, vena capita dorsalis.

**Figure 16:**
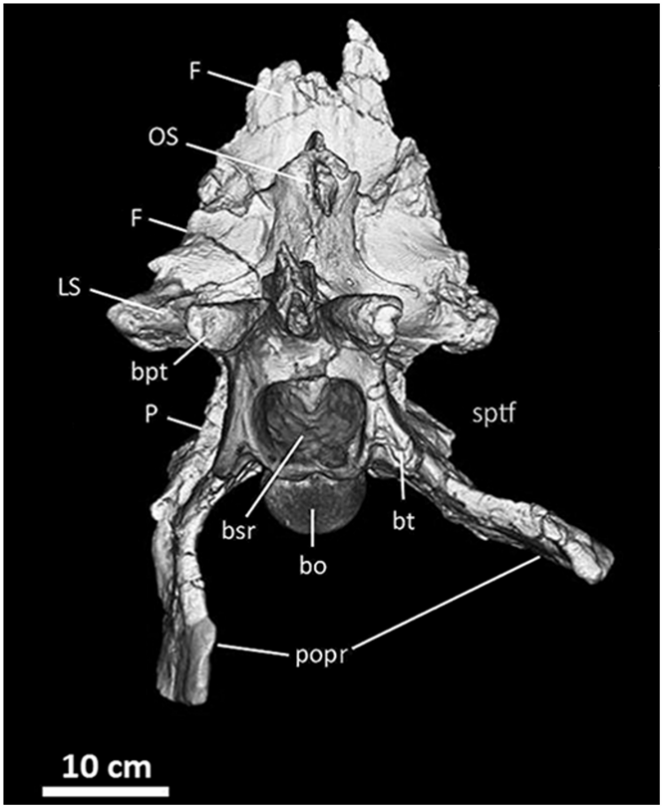
Frontals, parietals, and braincase of *Acrocanthosaurus atokensis* (NCSM 14345) in ventral view. Reconstructed from CT scan data. **bo**, basioccipital; **bpt**, basipterygoid process; **bsr**, basisphenoid recess; **bt**, basal tubera; **F**, frontal; **LS**, laterosphenoid; **OS**, orbitosphenoid; **P**, parietal; **popr**; paroccipital processes; **sptf**, supratemporal fossa.

**Figure 17:**
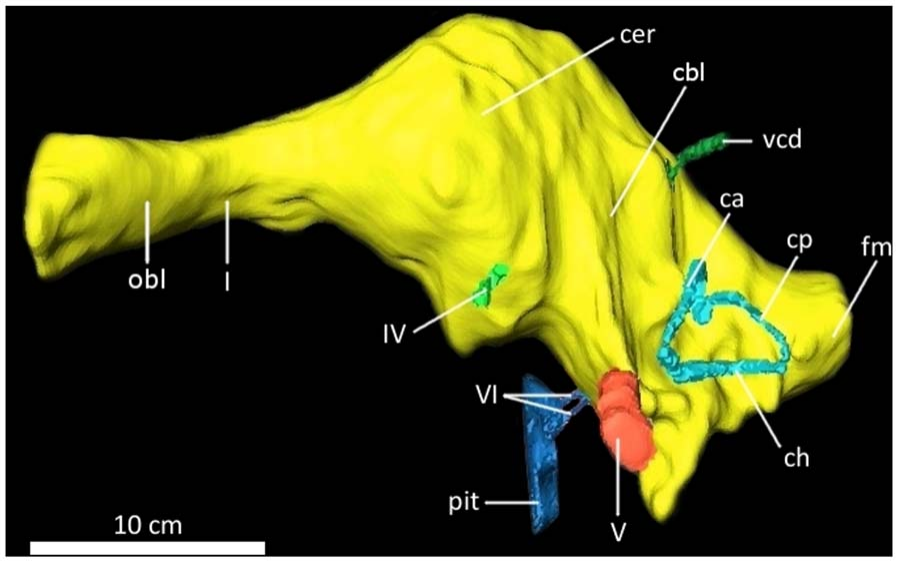
Digital endocranial endocast of the braincase of *Acrocanthosaurus atokensis* (NCSM 14345) in left lateral view. **ca**, anterior semicircular canal; **cbl**, cerebellum; **cer**, cerebrum; **ch**, horizontal semicircular canal; **cp**, posterior semicircular canal; **fm**, foramen magnum; **I**, olfactory nerve; **IV**, trochlear nerve; **obl**, olfactory bulbs; **pit**, pituitary; **V**, trigeminal nerve; **vcd**, vena capita dorsalis; **VI**, abducens nerve.

**Figure 18:**
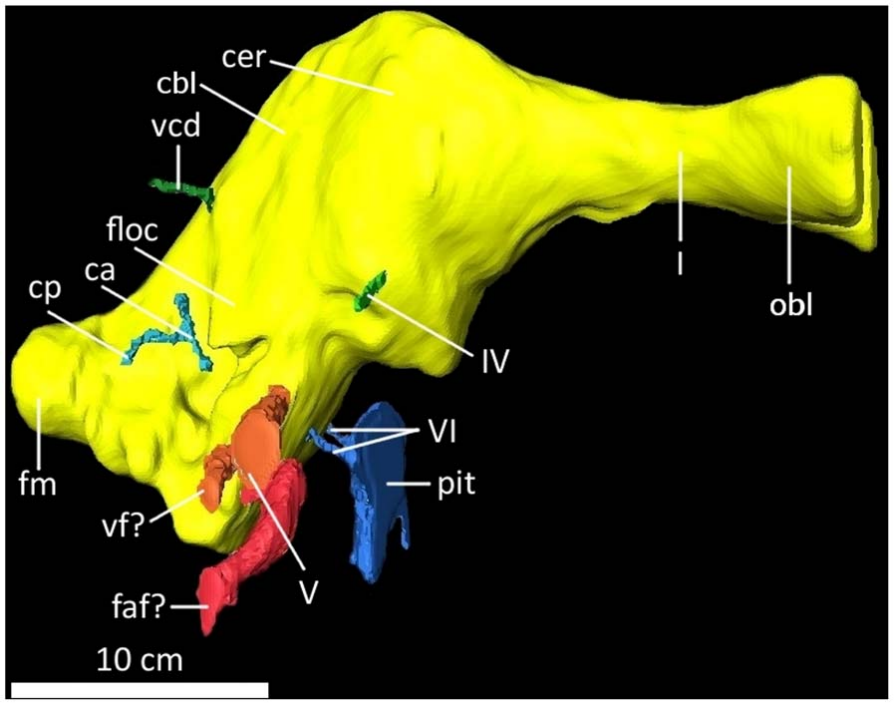
Digital endocranial endocast of the braincase of *Acrocanthosaurus atokensis* (NCSM 14345) in right lateral view. **ca**, anterior semicircular canal; **cbl**, cerebellum; **cer**, cerebrum; **ch**, horizontal semicircular canal; **cp**, posterior semicircular canal; **faf**, fossa acoustic-facialis; **floc**, flocculus; **fm**, foramen magnum; **I**, olfactory nerve; **IV**, trochlear nerve; **obl**, olfactory bulbs; **pit**, pituitary; **V**, trigeminal nerve; **vcd**, vena capita dorsalis; **vf**, vagus foramen; **VI**, abducens nerve.

**Table 1:** Measurements of *Acrocanthosaurus atokensis* specimen NCSM 14345, reported in centimeters.

Element	Right	*Left*	Element	Right	*Left*
**Premaxillary body, H**	10.99	*10.75*	**Occipital condyle, H**	5.59	
**Premaxillary body, L**	9.45	*9.84*	**Occipital condyle, W**	6.97	
**Premaxillae W**	15.10		**Vomer, L** *	46.10	
**Maxilla, H**	44.85	*38.58*	**Vomer, W**	3.66	
**Maxilla, L**	80.76	*82.33*	**Vomer, H**	4.45	
**Nasals, L**	75.45	N/A	**Palatine, H**	16.20	*16.55*
**Nasals, W**	12.72		**Palatine, L (maxillary-jugal process)**	31.30	*29.48*
**Lacrimal, H**	39.41	*35.97*	**Pterygoid, L** *	74.00	*78.40*
**Lacrimal horn, L**	33.70	*32.46*	**Pterygoid, H**	N/A	*23.73*
**Jugal, H**	32.11	29.97	**Ectopterygoid, L**	23.40	*23.02*
**Jugal, L**	51.77	*48.80*	**Ectopterygoid, W**	14.40	*15.51*
**Postorbital, H**	30.23	*29.50*	**Epipterygoid, H**	N/A	*15.56*
**Postorbital, L**	26.72	*28.75*	**Epipterygoid, L**	N/A	*5.80*
**Quadrate, H**	35.43	*35.20*	**Dentary, L**	83.10	*82.14*
**Quadrate, W (condyles)**	14.27	*13.85*	**Dentary, H (mid-tooth row)**	11.13	*10.47*
**Quadratojugal, L**	26.79	*26.60*	**Dentary, W (mid tooth row)**	4.55	*4.12*
**Squamosal, H**	16.70	*15.52*	**Surangular, L**	61.40	*59.08*
**Squamosal, L**	21.95	*20.90*	**Surangular, H**	16.44	*18.72*
**Prefrontal, H**	14.20	*14.36*	**Supradentary/Coronoid L**	N/A	*72.14*
**Prefrontal, L**	15.95	*16.17*	**Coronoid, H**	N/A	*8.10*
**Frontals, L**	19.10		**Supradentary, H**	2.24	*2.45*
**Frontals, W**	21.44		**Angular, L**	55.97	*61.50*
**Parietals, W (nuchal crest)**	15.82		**Prearticular, L**	69.05	*71.00*
**Foramen magnum, W**	3.54		**Splenial, L**	N/A	*60.51*
**Supraoccipital knob, W**	7.52		**Articular, W**	13.74	*14.25*

*Abbreviations:* **H**, maximum height; **L**, maximum length; **W**, maximum width;

*, measurement reconstructed from broken element; **N/A**, not available.

### Comparative material

The holotype specimen of *Acrocanthosaurus atokensis* (OMNH 10146) includes a braincase and fragmentary elements of the posterior skull and mandible recovered from the Trinity Formation (Aptian-Albian) of southeastern Oklahoma [23] (Table 2). An additional specimen (OMNH 10147) preserving only post-cranial material was discovered in the same area and formation as the holotype, and designated as the paratype specimen of *Acrocanthosaurus atokensis* [23]. Material referred to *Acrocanthosaurus atokensis* between 1950 and the late 1990s was limited to various descriptions of tooth material tentatively assigned to the taxon [59]–[61]. One specimen was named during that interval as the holotype of a new European species *Acrocanthosaurus altispinax* Paul 1988 based on the presence of elongate neural spines on its dorsal vertebrae [62]. However, this specimen was later recognized as referable to a spinosauroid from England [12], [14], [63]–[64], now called *Becklespinax altispinax*.

**Table 2:** Specimens with cranial elements referred to the taxon *Acrocanthosaurus atokensis*.

	OMNH 10146 (Holotype)	OMNH 10147 (Paratype)	SMU 74646	UNSM 497718, 49772–49776	NCSM 14345
Premaxilla	-	-	-	-	x
Maxilla	-	-	-	-	x
Nasal	-	-	-	-	x
Lacrimal	x	-	-	-	x
Jugal	o	-	o*	-	x
Postorbital	x	-	x	-	x
Quadrate	-	-	-	-	X
Squamosal	o	-	-	-	x
Quadratojugal	-	-	-	-	o
Parietal	x	-	-	-	x
Frontal	x	-	-	-	x
Prefrontal	-	-	-	-	x
Braincase	x	-	-	-	x
Pterygoid	-	-	-	-	X
Palatine	-	-	x	-	x
Vomer	-	-	-	-	X
Ectopterygoid	x	-	x*	-	x
Epipterygoid	-	-	-	-	X
Articular	x	-	-	-	x
Surangular	o	-	o	-	x
Angular	-	-	-	-	x
Dentary	-	-	-	-	x
Splenial	-	-	o	-	X
Prearticular	-	-	o	-	X
Supradentary	-	-	-	-	X
Teeth	x	-	x	x	x
Postcrania	x	x	x	-	x

*Abbreviations*: –, element not preserved; **o**, partial or fragmentary element present; **x**, complete element present; **X**, first complete element described for *Acrocanthosaurus atokensis*;

*, inaccurately sided in original description.

The past thirteen years have witnessed the description of new specimens crucial to understanding the morphology and phylogenetic affinities of *Acrocanthosaurus atokensis* (a list of specimens preserving material referable to the taxon is presented in Table 2). Harris [21] referred a specimen to *Acrocanthosaurus atokensis* from the Early Cretaceous of Texas that preserves a large amount of post-cranial material and several cranial elements (SMU 74646). Similar to the holotype specimen, the skull of SMU 74646 is largely incomplete and preserves only a fragmentary jugal, ectopterygoid, palatine, and posterior mandible. A postorbital is also preserved, but likely prepared after Harris' description.

Comparisons with the skull of *Acrocanthosaurus atokensis* are drawn from cranial material referred to several taxa consistently recovered within Allosauroidea (*e.g.*, *Aerosteon riocoloradensis*, *Allosaurus fragilis*, *Australovenator wintonensis*, *Carcharodontosaurus saharicus*, *Eocarcharia dinops*, *Giganotosaurus carolinii*, *Mapusaurus roseae*, *Neovenator salerii*, *Shaochilong maortuensis*, *Sinraptor dongi*, *Tyrannotitan chubutensis*, *Yangchuanosaurus shangyouensis*), as well as other taxa within Theropoda (*e.g.*, *Baryonyx walkeri* Charig and Milner 1986 [65], *Coelophysis bauri* Cope 1887 [66], *Herrerasaurus ischigualastensis* Reig 1963 [67], *Tyrannosaurus rex*). Table 3 provides a full list of evaluated cranial elements referable to Allosauroidea, and Table S1 describes the methods by which comparative material was assessed.

**Table 3:** Cranial elements known for 14 allosauroid taxa.

	*Monolophosaurus *	*Yangchuanosaurus *	*Sinraptor *	*Allosaurus *	*Neovenator *	*Eocarcharia *	*Tyrannotitan *	*Shaochilong *	*Acrocanthosaurus *	*Carcharodontosaurus *	*Mapusaurus *	*Giganotosaurus *	*Concavenator *	*Aerosteon *
Premaxilla	x	x	x	x	x	-	-	-	x	-	-	x	x	-
Maxilla	x	x	x	x	x	x	-	x	x	x	x	x	x	-
Nasal	x	x	x	x	x	-	-	x	x	x	x	x	x	-
Lacrimal	x	x	x	x	-	-	-	-	x	x	x	x	x	-
Jugal	x	x	x	x	-	-	x	-	x	x	x	-	x	-
Postorbital	x	x	x	x	-	x	-	-	x	x	x	x	x	x
Quadrate	x	x	x	x	-	-	-	x	x	-	x	x	x	x
Squamosal	x	x	x	x	-	-	-	-	x	-	-	-	-	-
Quadratojugal	x	x	x	x	-	-	-	-	x	-	-	-	-	-
Parietal	x	x	x	x	-	x	-	x	x	-	-	x	?	-
Frontal	x	x	x	x	-	x	-	x	x	x	-	x	?	-
Prefrontal	x	x	x	x	-	x	-	-	x	x	x	x	?	x
Braincase	x	?	x	x	-	-	-	x	x	x	-	x	?	-
Pterygoid	?	?	x	x	-	-	-	-	x	-	-	x	x	x
Palatine	?	x	x	x	x	-	-	-	x	-	-	-	x	-
Vomer	?	?	x	x	-	-	-	-	x	-	-	-	x	-
Ectopterygoid	?	?	x	x	-	-	-	-	x	x	-	x	?	-
Epipterygoid	?	?	x	x	-	-	-	-	x	-	-	-	?	-
Articular	x	x	x	x	-	-	-	-	x	-	x	-	x	-
Surangular	x	x	x	x	-	-	-	-	x	-	x	-	x	-
Angular	x	x	x	x	-	-	-	-	x	-	-	-	-	-
Dentary	x	x	x	x	x	-	x	-	x	x	x	x	x	-
Splenial	x	x	x	x	-	-	-	-	x	-	x	-	?	-
Prearticular	x	x	x	x	-	-	-	-	x	x	x	-	?	x
Supradentary	x	x	x	x	-	-	-	-	x	-	-	-	?	-

*Abbreviations*: **x**, complete or fragmentary element present**?**, element present but not described; **–**, element not preserved.

Despite a seemingly broad sample of comparative skull material, relatively few crania referred to taxa within Allosauroidea are extensively described or represented by multiple specimens. The most well-studied allosauroid skull is that of *Allosaurus fragilis*, known from several specimens with complete (or nearly complete) crania [27], [68]–[70]. In addition to *Allosaurus*, four allosauroid taxa are known from specimens preserving relatively complete skulls (*Sinraptor* [16], *Yangchuanosaurus* [45], *Carcharodontosaurus* [20], *Acrocanthosaurus* [1]), as is one putative carnosaur (*Monolophosaurus* [71]). Of these, only a skull referred to *Sinraptor* is monographed with multiple illustrations of every cranial element. Descriptions of partially prepared skulls of *Monolophosaurus* and *Yangchuanosaurus* are more limited, restricted to lateral and dorsal views of cranial, palatal, and mandibular elements, and medial views of the mandible [45], [71]–[73]. Crania of specimens referred to several basally-positioned carcharodontosaurian taxa are largely incomplete (*i.e.*, *Neovenator* [74]–[75], *Tyrannotitan* [39], *Eocarcharia* [49], *Australovenator* [48], and *Shaochilong* [37], [76]). Taxa recovered within Carcharodontosaurinae are known from more complete crania (*i.e.*, *Giganotosaurus* [35], [41], *Mapusaurus* [36], *Carcharodontosaurus* [20], [75], and *Concavenator* [25]).

## Results

### Cranial morphology of *Acrocanthosaurus atokensis*

The following sections provide a detailed description of the cranial anatomy of *Acrocanthosaurus atokensis* specimen NCSM 14345. Unless otherwise indicated, descriptions of the morphology in *Acrocanthosaurus* focus on NCSM 14345. Cranial morphologies of *Acrocanthosaurus* described in previous works [1], [21], [23] are cited appropriately; all other observations are made by the authors. Traditional anatomical nomenclature is most often used over veterinary terminology (*e.g.*, “anterior/posterior” instead of “rostral/caudal”).

### Nasal

The skull of NCSM 14345 (Figure 2) preserves the only nasal referable to *Acrocanthosaurus atokensis*. The left and right nasals are complete, but broken posteriorly near their contacts with the lacrimals. The left nasal is also broken anteriorly near its contact with the premaxilla (Figure 3), whereas the right nasal displays an additional break at mid-length. A portion of the ascending ramus of the right maxilla remains attached to the ventral surface of the right nasal, and the posterior portion of the left nasal is adhered to the medial surface of the left lacrimal horn.

The nasal forms the posterior margin of the external naris with its contact to the subnarial processes of the premaxilla, excluding the maxilla from participating in the opening [1]. An elongated narial fossa extends posterodorsally from the rim of the external naris and depresses the lateral surfaces of the nasal (Figures 3A, 36B). Ridges border the narial fossa dorsally and ventrally, and converge at the posterior margin of the fossa. The thin ventral ridge articulates with the ascending ramus of the maxilla and contacts the premaxilla anteriorly [1], and the thicker dorsal ridge forms the upper rim of the external naris with the supranarial process of the premaxilla (Figure 2). The narial fossa is highly elongated in *Acrocanthosaurus*, *Carcharodontosaurus*, *Concavenator*, and *Tyrannosaurus* [20], [25], [27]. In *Sinraptor*, *Allosaurus*, *Neovenator*, and *Monolophosaurus* [16], [50], [69], [71]–[72], the reduced long axis of the narial fossa gives the depression a more rounded, ovular shape. Rounded narial fossae are also found in the coelurosaur *Dilong paradoxus* Xu, Norell, Kuang, Wang, Zhao, and Jia 2004 [77], and in basal theropods such as *Herrerasaurus ischigualastensis* and *Coelophysis bauri*. *Giganotosaurus* and *Mapusaurus* have highly rugose nasals that lack any expansion of the narial fossa.

The lateral ridge of the nasal (Figure 3) participates in the dorsal margin of the antorbital fossa and contacts the lacrimal horn posteriorly [1]. In contrast to the rugose nasals of *Mapusaurus*, *Neovenator*, *Carcharodontosaurus*, *Concavenator*, and *Giganotosaurus* [20], [25], [35]–[36], [75], the nasal ridge of *Acrocanthosaurus* is relatively smooth as in *Sinraptor*, *Monolophosaurus*, and *Allosaurus*. Foramina above the antorbital fenestra perforate the nasal of *Acrocanthosaurus* [1]. These foramina are proportionally much smaller than the laterally-facing nasal pneumatic recesses of *Sinraptor* and *Allosaurus* (Figure 36A) which have been suggested to be homologous with ventrally-facing pneumatopores in *Concavenator*, *Giganotosaurus*, *Mapusaurus*, and *Neovenator* [36], [75]. However, these ventral pneumatopores are absent in *Acrocanthosaurus*. Along the ventral margin of the nasal, a narrow flange (referred to here as the “nasal-maxillary process”) projects anteroventrally to articulate with a notch along the dorsal margin of the ascending ramus of the maxilla (Figures 3, 36B). The nasal of *Carcharodontosaurus* (SGM-Din 1) also preserves this protrusion, but it is absent in specimens of *Sinraptor*, *Neovenator*, *Allosaurus*, and *Monolophosaurus*. Presence of the naso-maxillary process in *Mapusaurus* and *Giganotosaurus* is unclear, as rugosities cover the lateral surface of the nasals in these taxa. In medial view, a small ridge ventral and parallel to the roof of the nasal of *Acrocanthosaurus* flattens horizontally. The ridge is perforated posteriorly by three elongated foramina that open ventrally (Figure 3B) and likely represent foramina associated with the nasal vestibule [78]. Similarly positioned foramina also occur in *Allosaurus*.

### Premaxilla

The paired premaxillae preserved in NCSM 14345 (Figure 4) are the only premaxillary elements currently referred to *Acrocanthosaurus* (Table 2). In lateral view, the premaxillary body is taller than long (10.75×9.84 cm) [1], as in *Giganotosaurus* [35], *Yangchuanosaurus* [45], and several non-allosauroid theropods (*e.g.*, *Majungasaurus*, *Ceratosaurus* Marsh 1884 [79], *Tyrannosaurus* [80]–[82]). In *Allosaurus*, *Monolophosaurus*, *Neovenator*, and *Sinraptor*, the premaxilla is longer than tall, and this condition is exaggerated in the spinosauroid *Baryonyx walkeri* [65]. The premaxilla of *Acrocanthosaurus* has four alveoli [1], as in *Sinraptor* and *Giganotosaurus*. Five premaxillary alveoli occur in *Neovenator* and *Allosaurus*.

The supranarial and subnarial processes of the premaxilla of *Acrocanthosaurus* (Figure 2) extend posterodorsally to contact the nasal and form the anteroventral border of the external naris [1]. The subnarial process is dorsoventrally flattened, triangular in dorsal view, and excludes the maxilla from participating in the ventral margin of the external naris. The anterior region of the narial fossa depresses the rostrum between the supranarial and subnarial processes of the premaxilla (Figure 4). The medial view of the premaxilla is partially obscured in NCSM 14345, as the element is in contact with its counterpart to strengthen the mounted specimen. In posterior view, the small maxillary process articulates posteromedially with the maxilla, but does not surpass the posterior margin of the premaxillae as in *Sinraptor* and the tetanuran *Duriavenator* [83].

Foramina perforate the lateral surface of the premaxillary body and likely accommodated branching of the medial ethmoidal nerve and subnarial artery [1]. These premaxillary foramina in *Acrocanthosaurus* are shallower and less abundant than those in *Allosaurus* and *Neovenator*. An isolated, larger depression is present at the base of the right supranarial process (Figure 4B). *Sinraptor*, *Neovenator* and some specimens of *Allosaurus* (CM 1254; UUVP 1863) also possess a large foramen near the base of the supranarial process [16], [74].

### Maxilla

The left and right maxillae of NCSM 14345 represent the only such elements currently known for *Acrocanthosaurus*. Although the right maxilla is well-preserved, the left maxilla is missing seven teeth (alveoli 6–12) and a section of the posterior ramus above the fifth alveolus. The tooth of a crocodylomorph was removed from the left maxilla dorsal to the eleventh alveolus. The crocodylomorph tooth was overgrown by a thin layer of bone, suggesting that the event responsible for its emplacement likely occurred well before the death of this individual of *Acrocanthosaurus*. Lateral surfaces of the maxilla were previously described [1], although internal surfaces were not visible at that time.

The maxilla forms much of the anteroventral region of the skull in lateral view (Figures 2, 5). It contacts the premaxilla with a posterodorsally-sloped anterior margin as in *Sinraptor*, *Mapusaurus*, *Eocarcharia*, *Shaochilong*, and *Carcharodontosaurus* [13], [16], [36]–[37], [49]. The sloped maxillary-premaxillary contact in *Acrocanthosaurus* differs from that of *Allosaurus*, *Neovenator*, and *Monolophosaurus*, in which the margin is oriented dorsoventrally [69], [71], [74]. Posterodorsal to its contact with the premaxilla, the maxilla contacts the subnarial flange of the nasal with a slightly convex margin (Figure 5A), as in *Sinraptor*, *Mapusaurus*, *Carcharodontosaurus*, and *Eocarcharia*. The maxillae of *Allosaurus*, *Neovenator*, and *Monolophosaurus* are concave at the contact with the subnarial flange. Labial foramina (osteological correlates of neurovascular tracts [75]) pit the lateral surface of the anterior body of the maxilla. The majority of these depressions are small and isolated, similar to those present in *Allosaurus*, *Sinraptor*, and *Eocarcharia*. A few foramina form elongated, diagonal grooves in *Acrocanthosaurus* (Figure 5A), similar to the foramina along the alveolar margin in *Carcharodontosaurus* [20], [75]. However, the abundance of these grooved foramina in *Acrocanthosaurus* is substantially less than in *Carcharodontosaurus* [1].

Large, ovular foramina penetrate the maxilla of *Acrocanthosaurus* near the anteroventral corner of the antorbital fossa (Figure 5A) [1]. According to the terminology of Witmer [84], when two prominent openings are present in this region of the maxilla, the anterior opening is the ‘promaxillary fenestra’, while the smaller, posterior opening represents the ‘maxillary fenestra’. However, it is suggested here that the application of name ‘fenestra’ to these perforations is misleading since neither has a border formed by more than one element; the term ‘foramen’ more appropriately describes an opening contained within a single bone [85], but the standardized nomenclature is employed herein. The smaller (1.65 cm wide×3.70 cm tall), anteroventrally-placed promaxillary fenestra is partially obscured from lateral view in *Acrocanthosaurus* and tucked behind the rim of the antorbital fossa [1]. The medial vestibular bulla is broken, obscuring the nature of its connectivity with the maxillary antrum and promaxillary fenestra (Figure 5B).

The larger maxillary fenestra (3.94 cm wide×6.78 cm tall) lies posterior and slightly dorsal to the promaxillary fenestra, separated by a tall, narrow promaxillary strut. *Acrocanthosaurus* shares the presence of this opening with *Allosaurus*, *Sinraptor*, and *Neovenator*. In *Monolophosaurus* and *Carcharodontosaurus*, designation of similarly-placed openings as a ‘maxillary fenestra’ remains contentious [1], [9], [22], [84], whereas in *Mapusaurus* no maxillary fenestra is present [36]. Contrary to Currie and Carpenter [1], *Giganotosaurus* possesses a maxillary foramen, as the region anterior to this opening is broken and likely housed the promaxillary foramen [22], [75]. The size and position of the maxillary and promaxillary fenestrae in *Acrocanthosaurus* most closely resemble that of *Eocarcharia*. In *Eocarcharia*, a large, circular ‘accessory fenestra’ invades the posterodorsal ramus of the maxilla [49]. The maxilla of NCSM 14345 also possesses an accessory foramen in lateral view that was not discussed by Currie and Carpenter [1]. The accessory foramen opens ventromedially into medial apertures of the promaxillary and maxillary fenestra. Compared to *Eocarcharia*, in *Acrocanthosaurus* the accessory foramen is smaller, more elongated, and penetrates the medial shelf of the posterodorsal ramus dorsal to the promaxillary fenestra (Figure 5A).

Asymmetry of cranial pneumatic features is not uncommon in theropods [86] and occurs in the maxillae of *Acrocanthosaurus*. The accessory foramen of the left maxilla is tucked medially beneath the lateral shelf of the posterodorsal ramus and does not penetrate the medial shelf (Figure 5A), unlike in the right maxilla and the holotype specimen of *Eocarcharia* (MNN GAD2). A broader distribution of this feature within Allosauroidea is supported by the expression of a similarly positioned “foramen 4” ([16]: p. 2043) within the ascending ramus of the maxilla of *Sinraptor*. A fourth opening, the posterior fenestra in the maxillary antrum [84], is visible in posteromedial view near the juncture of the posterodorsal and posterior rami of the maxilla of *Acrocanthosaurus* (Figures 5B, 35B). This opening is internal to the postantral strut at the base of maxillary antrum and connects to the vestibular bulla, providing additional interconnectivity between the nasal cavity and antorbital fenestra. This posterior fenestra is absent in specimens of *Allosaurus* (UUVP 5499; BYU 725/5126; BYU 2028) and *Mapusaurus* (MCF-PVPH-108.169; MCF-PVPH-108.115), but present in *Sinraptor* as “pneumatic opening 10” ([16]: p. 2043), in *Carcharodontosaurus* (SGM-Din 1), and in many non-allosauroid theropods [84]. This region of the maxilla is broken in specimens referred to *Eocarcharia*, and its distribution within the remainder of Allosauroidea is poorly known.

The posterodorsal ramus of the maxilla separates the nasal from the antorbital fenestra and contacts the ventral surface of the lacrimal horn [1]. Along the anterodorsal margin of the posterodorsal ramus, a lateral shelf terminates anterior to a small notch for the naso-maxillary process of the nasal (Figures 5A, 36B). A similarly-positioned notch occurs along the anterodorsal margin in *Eocarcharia*. The broad medial shelf of the posterodorsal ramus is excluded from participating in the dorsal margin of the antorbital fossa by the lateral rim of the nasal. The shallow anterior extension of the antorbital fossa extends posterodorsally from the maxillary fenestra. This depression is narrow in *Acrocanthosaurus*, encompassing only half the width of the medial shelf. In *Allosaurus* the excavation occupies most of the width of the ramus [84]. Although the ascending ramus of *Acrocanthosaurus* does have small accessory pneumatic features, it lacks the extensive and complex pneumatic openings that perforate the ascending rami of *Sinraptor* and *Yangchuanosaurus* (Figure 36A).

The posterior ramus of the maxilla separates the tooth row from the antorbital fenestra [1], as it broadly contacts the ventral surface of the jugal and terminates ventral to the orbit. An anteroventral ridge slightly above the posterior ramus mid-height demarcates the ventral margin of the antorbital fossa. Posterior to the last maxillary alveolus, the ramus is deflected ventrally as in *Eocarcharia* and *Shaochilong* [37], [49], but unlike the straight posterior ramus of other allosauroids (*e.g.*, *Allosaurus*, *Sinraptor*, *Neovenator*). Medially, the posterior ramus of the maxilla contacts the palatine with a narrow shelf that tapers anteriorly (Figure 5B). This palatal contact terminates above the midline of the eighth maxillary alveolus in *Acrocanthosaurus*, as in *Eocarcharia*, *Carcharodontosaurus*, and *Neovenator*. In *Allosaurus* and *Sinraptor*, maxillary-palatal contact terminates further anteriorly above the seventh tooth (Figure 35A).

The interdental plates are fused and in medial view extend dorsoventrally across the main anterior body of the maxilla (Figure 5B). Interdental plate fusion is present in all allosauroid taxa except for *Sinraptor* [16]. Shallow, dorsoventral grooves indicate spacing between individual tooth plates. A horizontal ridge on the medial surface of the maxilla crosses the interdental plates (the ‘nutrient groove’ [81] or ‘groove for dental lamina’ [75]). The anterior end of this ridge is deflected anteroventrally at the level of the first alveolus. This ridge rises to mid-plate height across the first six maxillary alveoli before deflecting posteroventrally to contact the ventral margin of the palatal suture (Figures 5B, 35B). *Acrocanthosaurus* shares this sinuously-shaped ridge with *Neovenator* [87], *Eocarcharia*, *Carcharodontosaurus*, *Shaochilong* [37], *Mapusaurus*, and some megalosaurids [23]. In *Sinraptor* and *Allosaurus*, the ridge is straight (Figure 35A) and positioned closer to the tooth row.

### Jugal

Both jugals of NCSM 14345 are complete and appear morphologically similar to the left jugal of the holotype specimen of *Acrocanthosaurus* [23] and the right jugal of SMU 74646 [21]. The jugal from the holotype specimen is missing the posterior region, including the quadratojugal prongs, whereas the jugal of SMU 74646 lacks most of its postorbital and anterior processes.

The jugal of *Acrocanthosaurus* (Figure 6) is laterally compressed and tripartite. The anterior jugal process forms the posteroventral corner of the antorbital fenestra as the process broadly contacts the descending process of the lacrimal and is supported ventrally by the posterior ramus of the maxilla [1]. The antorbital fossa is demarcated by a curved ridge on the jugal that expands dorsally onto the lacrimal and anteriorly onto the maxilla (Figure 2). A foramen penetrates the jugal medial to this ridge (Figure 6B), as in *Sinraptor* [16], *Mapusaurus* [36], and *Monolophosaurus* [71] (although see [75]); the jugal of *Allosaurus* is apneumatic [72]. Posterior to the anterior jugal process in *Acrocanthosaurus*, a triangular postorbital process contacts the anterodorsal margin of the postorbital ventral ramus [1].

The posterior process of the jugal is split into two quadratojugal prongs that fit tongue-in-groove with the anterior ramus of the quadratojugal (Figure 6). The dorsal quadratojugal prong is more than twice as tall as the ventral prong in *Acrocanthosaurus* (4.4 cm and 2.17 cm, respectively). This ratio is observed in most allosauroid taxa except for *Allosaurus*, in which the ventral quadratojugal prong is consistently shorter (Figure 39). The ventral quadratojugal prong of the jugal in *Acrocanthosaurus* is thin, elongated, and overlaps most of the ventral margin of the anterior process of the quadratojugal. Between the two quadratojugal prongs, a small, rounded accessory prong is present laterally, but partially obscured in lateral view by overlap of the quadratojugal (Figure 6). This prong has not been described for *Acrocanthosaurus*, because the holotype specimen and SMU 74646 fail to preserve the posterior region of the jugal [21], [23]. The accessory prong on the lateral surface of the jugal of *Acrocanthosaurus* is distinct from the jugal of *Sinraptor*, in which the ventral quadratojugal prong is split into two processes and includes an exaggerated medial process overlapping the medial surface of the quadratojugal [16]. A small accessory prong is also preserved in *Mapusaurus* [36], *Tyrannotitan*, and possibly in *Carcharodontosaurus* (SGM-Din 1), but is absent in *Allosaurus*.

In medial view, the medial jugal foramen penetrates the jugal posterior to the junction of the quadratojugal prongs (Figure 6B). This foramen is expressed in SMU 74646 [21], and its presence in the holotype specimen of *Acrocanthosaurus* is likely because the jugal is highly pneumatic [23]. *Sinraptor* and *Carcharodontosaurus* also preserve a medial jugal foramen [16]. The left jugal of NCSM 14345 preserves an additional recess similar in size to the medial jugal foramen, but situated along the contact with the posterior ramus of the maxilla. *Sinraptor* also possesses a pneumatic opening in this region [16].

### Lacrimal

In addition to the left and right lacrimals of NCSM 14345, only the holotype specimen preserves lacrimal material referable to *Acrocanthosaurus*. The left lacrimal of the holotype is morphologically similar to those of NCSM 14345, although it is not as well-preserved and has a narrower descending process. Currie and Carpenter [1] described the lateral surface of the left lacrimal; medial surfaces were not visible at that time.

Aside from the dorsal boss of the postorbital, the lacrimal horn is one of the more laterally prominent features of the facial region in *Acrocanthosaurus*. Projection of the horn above the dorsal margin of the skull is reduced (Figure 2), consistent with *Carcharodontosaurus*, *Giganotosaurus*, *Concavenator*, and *Sinraptor*, but unlike the raised lacrimal horn of *Allosaurus* [1]. The anterior ramus of the lacrimal is relatively straight and long in dorsal view (∼32.5 cm), but the ramus curves laterally dorsal to the lacrimal pneumatic recess (Figure 38C). *Acrocanthosaurus* shares this curvature with *Carcharodontosaurus* and *Giganotosaurus*, whereas the lacrimal horns of *Sinraptor* and *Allosaurus* are straight in dorsal view.

The internal structure of the lacrimal pneumatic recess is well-preserved (Figure 7A). The lacrimal recess is assessable in the holotype specimen of *Acrocanthosaurus*, but the delicate septa dividing the openings have been crushed. Stovall and Langston [23] describe the pneumatic recess of the holotype as preserving two main openings, which differs from the single opening in NCSM 14345 [1]. However, both left and right lacrimal pneumatic recesses in NCSM 14345 are tri-radiate and divided by septa into three distinct cavities that extend anterodorsally, posteriorly, and posteroventrally. A single opening was also likely present in the holotype specimen, although breakage of the cavity caused it to appear to preserve multiple openings. Tri-radiate lacrimal pneumatic recesses are also present in *Allosaurus*, *Sinraptor*, and the coelurosaur *Tyrannosaurus* [84]. The lacrimal pneumatic recess in *Giganotosaurus* is also divided by at least one septum, but this condition is unknown for other carcharodontosaurids due to breakage of the lacrimal horns of *Carcharodontosaurus* and *Mapusaurus*.

Anterior to the primary lacrimal recess, additional openings are visible in both lacrimals of NCSM 14345, a feature not present in the holotype specimen of *Acrocanthosaurus*. These openings also occur in *Giganotosaurus*, *Concavenator*, *Sinraptor*, and some specimens of *Allosaurus*. In posterior view, the naso-lacrimal canal (‘lacrimal duct’ [16]) perforates the lacrimal of *Acrocanthosaurus* with a single foramen extending anterodorsally, as in *Allosaurus*. However, *Allosaurus* preserves this naso-lacrimal canal and several ‘orbital recesses’ that excavate the posterior margin of the lacrimal [84]. Multiple posterior lacrimal foramina are similarly present in *Sinraptor* [16] and *Mapusaurus* [36], but these features are absent in *Acrocanthosaurus*.

The lateromedially-flattened descending process of the lacrimal articulates broadly with the jugal [1]. This process is comprised by distinct medial and lateral layers that are separated by a deep sulcus along the anterior margin of the lacrimal (Figure 37). *Acrocanthosaurus* shares this characteristic with *Carcharodontosaurus*, *Concavenator*, and *Giganotosaurus*. In *Sinraptor*, *Allosaurus*, and *Monolophosaurus*, the descending process is not separated by a sulcus and instead has a rounded anterior margin. The lateral layer of the descending process in *Acrocanthosaurus* protrudes anteriorly into the antorbital fenestra [1] to demarcate the posterior margin of the antorbital fossa, while the medial layer occupies the edge of the antorbital fenestra (Figures 2, 7). The lateral layer also protrudes posteriorly into the orbital fenestra, as in *Giganotosaurus* and *Mapusaurus*. In contrast, the posterior margin of the lacrimal of *Allosaurus*, *Monolophosaurus*, *Concavenator*, and *Sinraptor* is nearly straight.

Medially, the lacrimal of *Acrocanthosaurus* preserves several anteroposteriorly-oriented ridges along the medial surface of the lacrimal horn that articulate with the nasal and maxilla anteriorly (Figure 7B). The ridges contact the prefrontal posterior to their contact with the nasal, at which point the ridges display a ventral curvature. The posterior margin of the lacrimal horn contacts the postorbital in *Acrocanthosaurus*, as in *Giganotosaurus*, *Carcharodontosaurus*, and *Mapusaurus* [1], [20], [35]–[36]. The lacrimal and postorbital are separated by a gap in *Sinraptor*, *Allosaurus*, and *Monolophosaurus* [16], [69], [71] that permits the prefrontal to be seen when the skull is in lateral view.

### Postorbital

The left and right postorbitals of NCSM 14345 are complete. The holotype specimen of *Acrocanthosaurus* preserves a left postorbital [23], although the orbital brow and anterior margin of the postorbital are weathered and broken. Additionally, SMU 74646 has an undescribed, fragmentary right postorbital with a reconstructed ventral ramus and a tall dorsal boss.

The postorbital of *Acrocanthosaurus* is a robust, tripartite element that protrudes laterally from the dorsal margin of the skull (Figures 8, 40, 41). A rugose, sinusoidal orbital boss is present posterior to contact with the lacrimal and forms the roof of the orbit. The boss is split in lateral view by a sinuous vascular groove that extends along its entire length anteroposteriorly. The morphology and vascularization of this boss in *Acrocanthosaurus* is similar to that observed in *Concavenator*, *Mapusaurus* and *Carcharodontosaurus* [22], [25], and its presence is attributed to the possible fusion of a palpebral bone to the postorbital [36]. The postorbital in *Eocarcharia* displays a vascular groove along the anterior half of the orbital boss. *Giganotosaurus* lacks this vascular groove completely, although weathering of the bone surface may have removed this feature. The likely presence of a vascular groove on the postorbital of *Giganotosaurus* is supported by the presence of a palpebral bone covering the dorsal surface of its postorbital [41]. Palpebral-postorbital fusion is probable in *Acrocanthosaurus* as well, and although no sutures between the elements are visible, small fossae along the posterior termination of the dorsal boss of the postorbital may indicate postorbital-palpebral contact as in *Mapusaurus* and *Eocarcharia* [36], [49]. Postorbital rugosity has been noted in specimens of *Allosaurus* [69], although this taxon and *Monolophosaurus* lack a laterally expanded, vascularized postorbital boss. Posterior to the orbital boss of *Acrocanthosaurus*, a triangular, tapering process fits into a grooved articulation with the squamosal (Figure 8).

The descending ramus of the postorbital tapers along its posterior margin near the contact with the jugal. Together these elements form the anterior edge of the lateral temporal fenestra. The left postorbital preserves a triangular flange (‘intraorbital process’ [49]) anteriorly along the descending ramus, a feature not previously described for *Acrocanthosaurus*. This flange protrudes into the orbital fenestra and denotes the lower limit of the ocular cavity with the posterior projection of the descending process of the lacrimal (Figures 2, 8). The right postorbital of NCSM 14345 and the postorbital of the holotype specimen have broken anterior margins, inferred by Brusatte and Sereno [22] to represent missing intraorbital processes. The robustness of the intraorbital process in *Acrocanthosaurus* resembles that of the abelisaurid *Carnotaurus sastrei*. The carcharodontosaurian taxa *Carcharodontosaurus*, *Concavenator*, *Eocarcharia*, and *Giganotosaurus* also possess postorbitals with an intraorbital process [13]–[14], [35], [49], although the protrusion is laterally compressed, triangular, and proportionally smaller in these taxa (Figure 38). A lateromedially-flattened intraorbital process is also present in *Tyrannosaurus* and *Majungasaurus* [27], [80], although the process is dorsoventrally taller in these taxa than in members of Allosauroidea. In *Allosaurus*, *Monolophosaurus*, *Sinraptor*, and *Yangchuanosaurus*, the anterior margin of the postorbital is smooth and lacks an intraorbital process (although a small convexity is present in *Monolophosaurus* and *Sinraptor* [72]), a condition shared with *Herrerasaurus*, *Coelophysis*, and most other non-allosauroid theropods [88]–[89].

The medial surface of the postorbital of *Acrocanthosaurus* has a medially-expanded shelf that contacts the prefrontal and frontal anteriorly. Several small fossae are tucked beneath the margin of the shelf near its contact with the parietal and laterosphenoid (Figure 8B). Here, the shelf is divided into a posterior shelf and a ventral ridge. The posterior extension of the shelf parallels the dorsal surface of the postorbital, and is overlapped laterally by the squamosal. The ventral ridge is curved and terminates along the ventral ramus of the postorbital near contact with the jugal. Similar medial shelf morphologies are present on the postorbitals of *Giganotosaurus*, *Mapusaurus*, and *Sinraptor*. The anterior portion of this shelf that contacts the prefrontal of *Acrocanthosaurus* appears similar in morphology and location to a shelf figured for the postorbital of *Eocarcharia*, although in *Eocarcharia* this shelf contacts the frontal [49].

In dorsal view, the postorbital is lateromedially expanded, pitted with small fossa, and flattened except for the raised orbital boss along its lateral margin. The posterior region of the dorsal postorbital surface is depressed by the supratemporal fossa. The margin of this depression is curved medially near its expansion onto the frontal and parietal (Figure 41). *Acrocanthosaurus* shares a posteriorly-positioned depression of the dorsal surface of the postorbital with *Carcharodontosaurus*, *Mapusaurus*, and *Eocarcharia*. In *Allosaurus* and *Sinraptor*, the expression of the supratemporal fossa on the dorsal surface of the postorbital expands further anteriorly to approach the anterior margin of the postorbital [10], [42].

### Prefrontal

The first prefrontal material referred to *Acrocanthosaurus* is from NCSM 14345 (Figure 9), of which the dorsal surfaces have been described. Prefrontal exposure in dorsal view is minimal, and the relatively small element appears as a triangular wedge between the lacrimal horn, frontal, postorbital, and posterior process of the nasal [1]. The prefrontal contacts the lacrimal with a flattened articular surface pitted by numerous small depressions (Figure 9B). The prefrontal-lacrimal contact is not fused in *Acrocanthosaurus*, *Sinraptor*, *Eocarcharia* and *Allosaurus*; the prefrontal is fused to the lacrimal in *Giganotosaurus*, *Mapusaurus*, and *Carcharodontosaurus* [1], [22], [49].

The medial articular surface of the prefrontal is auriform in *Acrocanthosaurus* (Figure 9A), similar to *Sinraptor* [16] but unlike the triangular prefrontal of *Allosaurus* [69]. Blade-like processes extend anteriorly and ventrally from the main body of the prefrontal to contact the frontal and nasal, respectively. These processes converge upon the body of the prefrontal. Posteriorly, the anterior blade forms a ridge upon the body of the prefrontal that curves posteromedially to surround a deep sulcus. The ventral blade of the prefrontal contacts the frontal and curves posterodorsally to meet the rounded ridge formed by the anterior blade. Posterior to this ridge, a small flange contacts the postorbital.

### Quadratojugal

The skull of NCSM 14345 preserves the only quadratojugal material referred to *Acrocanthosaurus*. The dorsal rami of both quadratojugals are broken (Figure 10), and the medial surfaces of the quadratojugals are obscured by close contact with the quadrate. The quadrate and quadratojugal were cast in articulation.

The quadratojugal of *Acrocanthosaurus* is an L-shaped bone at the posteroventral corner of the cranium that forms the majority of the posterior margin of the lateral temporal fenestra. The lateral surface of the quadratojugal is relatively smooth and unornamented. The right quadratojugal, at the base of its dorsal ramus, preserves a small lateral fossa. This ramus would have likely contacted the precotyloid process of the squamosal dorsally, and the articulation of these processes is inferred to have curved anteriorly into the lateral temporal fenestra [1] to create a convex posterior margin of the fenestra in lateral view. The shape of the quadratojugal immediately below its dorsal breakage suggests that *Acrocanthosaurus* likely has a narrow dorsal quadratojugal ramus, as in *Sinraptor*, *Monolophosaurus*, and *Yangchuanosaurus*, but unlike the anteroposteriorly broader dorsal rami of *Allosaurus* and *Tyrannosaurus* [90]. The anterior ramus of the quadratojugal is trifurcated to fit tongue-in-groove between the dorsal and ventral quadratojugal prongs of the jugal (Figures 10A, 10C). The medial projection of the anterior ramus overlaps the medial surface of the jugal. The forked lateral projection sutures tightly between the quadratojugal prongs of the jugal and covers the small accessory prong of the jugal in lateral view. The convex posterior surface of the quadratojugal is curved along its contact with the quadrate (Figures 10A, 10B). The quadratojugal terminates ventrally at the dorsolateral surface of the lateral condyle of the quadrate. This quadrate-quadratojugal suture extends dorsally and terminates as the quadratojugal is deflected anterolaterally to contact the squamosal.

### Quadrate

Both left and right quadrates of NCSM 14345 are relatively intact (Figure 10), although the pterygoid wing of the right quadrate is broken and reconstructed. The posterior surface of the quadrate of *Acrocanthosaurus* was previously described [1], and NCSM 14345 preserves the only quadrate material referred to the taxon.

The medial condyle of the quadrate is positioned further posteriorly than the lateral condyle (Figure 10C), although the lateral condyle is wider in posterior view (Figure 10B), similar to the condition in most theropods [9]. The quadratojugal overlaps the quadrate and obscures most of the lateral surface of the quadrate from view. Along the quadrate-quadratojugal suture, the quadrate is pierced posteriorly by the quadrate foramen. Similar to *Allosaurus*, *Giganotosaurus*, *Mapusaurus*, and *Sinraptor* [16], [35]–[36], [69], most of the quadrate foramen of *Acrocanthosaurus* is enveloped by the quadrate, with the quadratojugal forming a reduced portion of the lateral rim of the opening (Figure 10B). In *Monolophosaurus* and *Tyrannosaurus*, the quadratojugal participates more extensively in the lateral rim of the quadrate fenestra [9], [72], [82]. Dorsal to the quadrate fenestra, a large depression (5.80 cm tall×3.44 cm wide) referred to here as the ‘posterior pneumatic recess’ penetrates the body of the quadrate and is split into two blind cavities by a thin septum. *Acrocanthosaurus* shares the presence of posterior quadrate pneumaticity with *Aerosteon*, *Giganotosaurus*, and *Mapusaurus* [10]. In *Giganotosaurus* and *Mapusaurus*, the pneumatic recess lacks a visible septum (Figure 44). The quadrate fenestra of *Aerosteon* is of a similar size and position to the posterior pneumatic recess of *Acrocanthosaurus*, although in *Aerosteon* the quadrate fenestra opens completely through the quadrate and is accompanied ventrally by a large blind fossa (‘pneumatocoel’ [51]).

The lateromedially-flattened pterygoid wing projects anteriorly from the quadrate to articulate with the quadrate ramus of the pterygoid. In medial view, the left quadrate preserves a large, rounded pneumatic recess (3.27 cm tall×3.73 cm wide) within the posteroventral corner of the pterygoid wing (Figure 10C). The ventral surface of the pterygoid wing of the quadrate forms the floor of this depression, referred to here as the ‘medial pneumatic recess’. A septum splits the medial pneumatic recess of the quadrate of *Acrocanthosaurus*, similar to the posterior pneumatic recess. *Giganotosaurus* and *Mapusaurus* also preserve a medial pneumatic recess in a comparable position. However, in *Giganotosaurus* the recess is small and round, and in *Mapusaurus* the recess is anterodorsally elongated and undivided. Quadrate material referred to *Allosaurus*, *Shaochilong*, and *Sinraptor* preserves a shallow depression in this region [16], [37], [69], but lacks a sharply defined medial pneumatic recess.

### Squamosal

The intact left and right squamosals of NCSM 14345 are the most complete squamosal elements referred to *Acrocanthosaurus*. Squamosal material from the holotype specimen includes a fragmentary left squamosal missing most of the quadratojugal and postcotyloid processes [23]. Both squamosals of NCSM 14345 are tri-radiate elements at the posterodorsal margin of the skull that form the posterodorsal corners of the lateral temporal fenestrae (Figures 2, 11). The dorsal process of the squamosal is lateromedially broad, and its lateral surface bears a rectangular suture for the posteriorly projecting squamosal process of the postorbital (Figure 11A). The medial surface of the dorsal process contacts the parietal with an anteriorly tapering, flat surface. The lateral margin of the nuchal crest is also preserved on the left squamosal.

The quadratojugal process of the squamosal extends anteroventrally into the lateral temporal fenestra [1]. The postcotyloid process of the squamosal is expanded and triangular in lateral view (3.70 cm wide neck, 6.27 cm wide distal expansion; Figure 11A), and wraps around the posterodorsal edge of the quadrate cotyle. The expanded distal end of the postcotyloid process in *Acrocanthosaurus* contrasts with distally-tapering processes in *Allosaurus* and *Monolophosaurus* (Figure 42). An expanded postcotyloid process is interpreted as being present in *Sinraptor* ([16]: p. 2048), but missing squamosal material prevents the confirmation of this morphology. Because the postcotyloid process is not distally expanded in *Yangchuanosaurus*, it is therefore possible that in Sinraptoridae (*i.e.*, *Sinraptor* and *Yangchuanosaurus* in this analysis) the postcotyloid process of the squamosal is tapered, as in other basally-positioned allosauroids.

In ventral view, the squamosal appears triangular and quadri-radiate (Figure 11B). A small, blind fossa penetrates the posterodorsal corner formed by the junction of the dorsal and quadratojugal processes of the squamosal. This opening occurs in *Tyrannosaurus* and *Majungasaurus* [80], [82], but not in the allosauroids *Allosaurus* and *Sinraptor* [16], [69].

### Frontal

The frontal, parietal, and braincase elements of NCSM 14345 are fused, as in the holotype specimen of *Acrocanthosaurus* [23]. The paired frontals of *Acrocanthosaurus* are dorsoventrally flattened and form the majority of the cranial surface dorsal to the orbital and olfactory regions of the braincase (Figures 12–16). The frontals were cast and are presently mounted in articulation with the parietal and orbitosphenoid, obscuring the connective surfaces among those elements. The suture between the frontals is completely fused [1], and the paired frontals form a triangular shape in dorsal view (Figure 14). Frontal fusion also occurs in the holotype specimen of *Acrocanthosaurus* [23], and in *Carcharodontosaurus*, *Giganotosaurus*, *Eocarcharia*, and *Shaochilong* [41], [49], [75], [76]. The frontals of the allosauroids *Allosaurus* and *Sinraptor* are unfused [76]. The frontal of *Acrocanthosaurus* contacts the posterior margin of the nasal with a flange-like triangular process. This process is exposed dorsally and slightly underlies the nasals, as in *Allosaurus*, *Eocarcharia*, *Giganotosaurus*, and *Shaochilong* [41], [49], [69], [76]. The nasal process of the frontal of *Sinraptor* extends proportionally further anteriorly beneath the nasal [16].

The frontal of *Acrocanthosaurus* contacts the prefrontal and postorbital anterolaterally and exhibits a shallow depression at the junction of these elements [1]. Posterior to this contact, the frontal displays a steep rim that flattens near its lateral contact with the postorbital. This rim forms the anteromedial margin of the supratemporal fossa (Figure 14). Posterior to this rim, the frontoparietal suture forms a sharply-raised ridge that expands laterally across the supratemporal fossa of the frontal and shallows near the postorbital contact. This ridge is pronounced and appears as a protuberance adjacent to the laterally-facing shelf of the supratemporal fossa, a condition also present in *Eocarcharia* [49] and *Sinraptor*. In ventral view, the frontal is separated from the orbitosphenoid by an anteroposteriorly-oriented sulcus that curves laterally near its contact with the laterosphenoid (Figure 16).

### Parietal

Similar to the frontals of NCSM 14345, the parietals are also fused. This occurs in the holotype specimen of *Acrocanthosaurus* and the carcharodontosaurids *Carcharodontosaurus*, *Giganotosaurus*, and *Shaochilong* [41,75, –76]. The anterior portion of the parietal contacts the frontal and extends laterally to contact the postorbital (Figures 12, 14, 16). The parietals contact each other along the midline of the skull, forming a flat, anteroposteriorly-oriented crest between the transverse nuchal crest and the frontals (Figure 14). In *Acrocanthosaurus*, the lateral margin of this crest is oriented vertically to form the medial wall of the supratemporal fossa, as in *Allosaurus*, *Monolophosaurus*, and *Sinraptor*. This crest is proportionally wider in *Carcharodontosaurus* and *Giganotosaurus*, and in these taxa the transverse nuchal crest is shifted forward to overlap the posteromedial corner of the supratemporal fossa [41], [75]. The relative size and length of the supratemporal fossa in *Acrocanthosaurus* are similar to that of *Eocarcharia*, and both taxa have proportionally longer and larger fossae than in *Carcharodontosaurus*, *Giganotosaurus*, and *Shaochilong*, but smaller than in *Sinraptor* and *Allosaurus* [76].

The parietal of *Acrocanthosaurus* forms the posterior wall of the supratemporal fossa. The transverse nuchal crest extends posterolaterally from the midline to contact the dorsal surface of the exoccipital process. Anteroventral to the nuchal crest, the parietal-laterosphenoid contact is slightly distorted by posterior crushing of the skull. Posterodorsally, the parietals contact the supraoccipital process near the midline of the braincase, although the lateral extent of this contact is also damaged (Figures 14, 15). The nuchal crest surrounds the supraoccipital of *Acrocanthosaurus* and exhibits a squared morphology in posterodorsal view as in *Sinraptor* [16], but unlike the rounded nuchal crest in *Allosaurus*, *Giganotosaurus*, and *Monolophosaurus* [19], [41], [71]. Additionally, the dorsal margins of the parietals are even with or slightly lower than the supraoccipital in *Acrocanthosaurus*. In *Allosaurus* and *Sinraptor*, the nuchal crest of the parietals extends above the supraoccipital.

### Braincase

The braincase is well-preserved, although several of the sutures, cranial nerve exits, and delicate bony processes (*e.g.*, preotic pendant, interorbital septum, parasphenoid, stapes) have been lost or distorted by crushing of the specimen (Figures 12–16). Furthermore, application of stabilizing material to prevent additional braincase breakage has obscured several openings and sutures. Sutures that are visible between braincase elements are noted in the description by explicitly mentioning the contact between two elements. An endocranial endocast generated from X-ray computed tomographic (CT) scans of the braincase of the holotype specimen has been described [91], and is compared with an endocast generated from the braincase of NCSM 14345 (Figures 17, 18).

The orbitosphenoid is the anterior-most element of the braincase and is bordered dorsally by the parietal (Figure 12). Sizable openings for the exit of the olfactory nerves (I) excavate the orbitosphenoid anteriorly. As in *Giganotosaurus* [41] and *Carcharodontosaurus* [75], openings for the olfactory nerve are cylindrical and separated by the mesethmoid in anterior view (Figure 13). In *Allosaurus* and *Sinraptor*, the exit for the olfactory nerve is expressed as a singular opening [16]. This may be an artifact of preservation in *Sinraptor* [91], although multiple specimens of *Allosaurus* lack an ossified mesethmoid (*e.g.*, UUVP 5961, 7145, 16645; BYU 671/8901). The ventral surface of the orbitosphenoid is broken in all specimens currently referred to *Acrocanthosaurus*, although remnants of an interorbital septum have been proposed in NCSM 14345 [76]. In *Giganotosaurus* and *Carcharodontosaurus*, this region clearly preserves an interorbital septum that connects the orbitosphenoid to the parasphenoid region [41]. The interorbital septum is absent in *Allosaurus*, *Eocarcharia*, *Shaochilong*, and *Sinraptor* [76].

The orbitosphenoid also preserves exits for the optic (II) and oculomotor nerves (III). In the holotype specimen, it is unclear whether the exit for the optic nerve and its accompanying vasculature are preserved as a single opening [91] as in *Allosaurus* and *Sinraptor*, or with two foramina as in *Carcharodontosaurus* and *Giganotosaurus*. The braincase preserves a single opening that is overlain by a lateromedially-flattened, tapering flange of bone in the approximate region for the exit of the optic nerve (Figure 12). However, this nerve exit was not reconstructed in the endocranial endocast (Figures 17, 18). The exit for the oculomotor nerve (III) is proximal to the optic nerve and exits posteroventrally to the optic nerve (C-II) on the right side of the braincase. The thin, bony process separating the optic and oculomotor nerves is broken. Crushing and specimen reconstruction have also obscured exits for the trochlear nerve (IV), which is located slightly posterodorsal to the ocular nerve exit in the braincase of the holotype specimen [23]. However, exits for the trochlear nerve (C-IV) are reconstructed in the appropriate region of the orbitosphenoid from both sides of the endocast (Figures 17, 18). The presence of a small, closed pit on the right side of the orbitosphenoid supports this observation. The pituitary fossa (Figures 12, 13) is composed of the basisphenoid and parasphenoid complex and is located ventral to the optic and oculomotor foramina. A more complete pituitary fossa is preserved in the braincase of the holotype specimen [23]. Openings for the abducens nerve (VI) are reconstructed on the endocast (Figures 17, 18) but are not visible on the exterior surface of the braincase. This paired nerve exit connects to the lateral margins of the posterior region of the pituitary fossa, as in the holotype specimen of *Acrocanthosaurus* [91] and in *Allosaurus*, *Carcharodontosaurus*, *Shaochilong*, and *Tyrannosaurus* [37], [90], [92]–[93].

Posteroventral to the laterosphenoid, the prootic is perforated by exits for the trigeminal (V) and palatine branch of the facial cranial nerves (VIIp), although the latter opening could not be reconstructed on the endocast. The foramen for the trigeminal nerve is large and ovular (Figure 12). This foramen exits the braincase lateroventrally between the prootic and the laterosphenoid in many large theropods [76], [80], although in NCSM 14345 the suture between these elements is not obvious. The foramen for the trigeminal nerve of *Acrocanthosaurus* exists as a single opening (Figure 17) as in *Carcharodontosaurus* and *Giganotosaurus*, but unlike the split trigeminal opening in *Allosaurus* and *Sinraptor* [76], [91]. Ventral and slightly anterior to the trigeminal foramen, the foramen for the palatine branch of the facial nerve (VIIp) opens posteroventrally. A small pit posterior to the facial nerve (VIIp) may represent the exit for the hyomandibular branch of the facial nerve (VIIh) from the braincase. The right prootic does not preserve openings for either palatine or hyomandibular branches of the facial nerve, but small foramina obscured by consolidants may represent where these nerves exited the braincase. The holotype specimen preserves exits for both of these nerves, and *Acrocanthosaurus* shares the presence of a branched facial nerve with *Shaochilong* and possibly *Giganotosaurus* [37], [91]. Ventral to the trigeminal and facial nerve exits, the left preotic pendant is broken at its base, permitting the assessment of the internal carotid artery (Figure 12).

**Figure 19:**
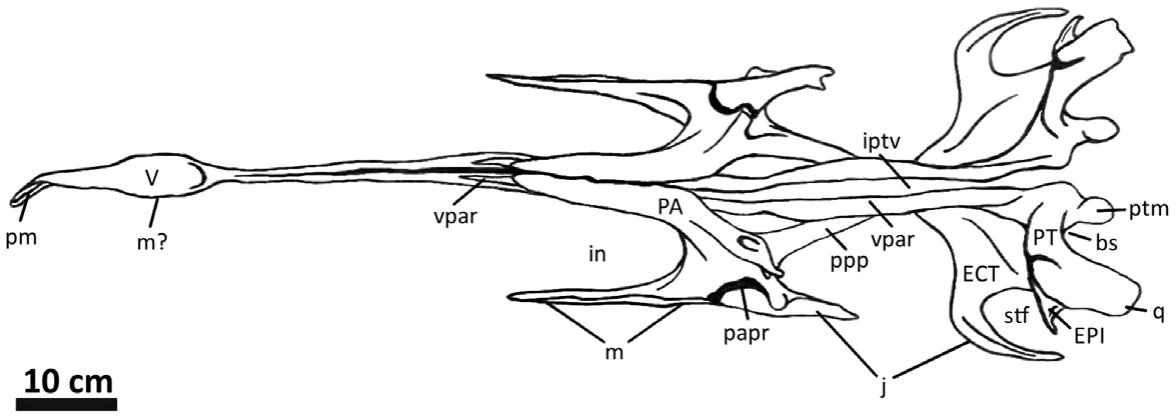
Palatal complex of *Acrocanthosaurus atokensis* (NCSM 14345) in dorsal view. **bs**, basisphenoid contact; **ECT**, ectopterygoid; **EPI**, epipterygoid; **j**, jugal contact; **in**, internal naris; **iptv**; interpterygoid vacuity; **m**, maxillary contact; **PA**, palatine; **papr**, palatine pneumatic recess; **pm**, premaxillary contact; **PT**, pterygoid; **ptm**, pterygoid medial process; **ppp**; pterygoid process of palatine; **q**, quadrate contact; **stf**, subtemporal fenestra; **V**, vomer; **vpar**, vomeropalatine ramus of the pterygoid.

Below the exoccipital and orbitosphenoid, the basisphenoid-parasphenoid complex forms the anteroventral region of the braincase (Figures 12, 13, 16). Unlike an exceptionally-preserved specimen of *Allosaurus* (UUVP 5961) [69], the gracile parasphenoid processes of specimens referred to *Acrocanthosaurus*, *Giganotosaurus*, *Carcharodontosaurus*, and *Sinraptor* are damaged or broken. In ventral view, a dorsally expanded, sub-cylindrical basisphenoid recess is developed between the basipterygoid processes and the basal tubera. A thin septum splits the basisphenoid recess dorsally (Figure 16). In *Acrocanthosaurus* and *Carcharodontosaurus* this recess is significantly deeper than in *Allosaurus* and *Sinraptor* [75]. Anteroventrally, the basipterygoid process articulates between the quadrate ramus and medial process of the pterygoid (Figure 19).

The most posterodorsal region of the braincase is composed of the supraoccipital, which articulates anteriorly with the transverse nuchal crest of the parietal and ventrally with the basioccipital (Figures 14, 15). The dorsal projection of the supraoccipital (‘supraoccipital wedge’ [9]) preserves a fold along its midline that separates the process into two distinct knobs termed the “double boss” ([1], p. 217). This boss narrows ventrally and gives the projection a triangular, wedge-like appearance in posterior view (Figure 15). Supraoccipitals of *Carcharodontosaurus*, *Giganotosaurus*, *Shaochilong*, and *Sinraptor* lack this fold, and instead preserve a single posterior ridge [16], [41], [75], [76]. Some specimens of *Allosaurus* preserve a folded supraoccipital wedge (DINO 11541) [19], although others exhibit a singular medial ridge (BYU 725/17879). Paired exits for the ‘vena capita dorsalis’ [84] penetrate the supraoccipital ventral to the supraoccipital wedge. The supraoccipital is bordered laterally by the exoccipitals. Similar to *Giganotosaurus*, the supraoccipital of *Acrocanthosaurus* participates ventrally in the dorsal margin of the foramen magnum but does not contact the basioccipital [41].

The exoccipital participates in the dorsolateral surface of the spherically-shaped occipital condyle, but the basioccipital comprises the majority of this structure (Figures 14, 15). A large opening penetrates the left exoccipital of *Acrocanthosaurus* along the neck of the occipital condyle and likely represents a paracondylar opening as seen in *Shaochilong* and other carcharodontosaurid taxa [37], [41]. This opening may have also been present in the right exoccipital, but medial deflection of the right paroccipital processes has deformed this region (Figure 14). The exoccipitals do not meet medially on the occipital condyle; instead, a thin dorsal exposure of the basioccipital separates the exoccipitals and forms the ventral margin of the foramen magnum. The basioccipital extends anteriorly into the foramen magnum and is depressed by a shallow, anteroposteriorly-oriented sulcus (Figures 14, 15). The paroccipital process of the exoccipital of *Acrocanthosaurus* is ventrally deflected well below the foramen magnum and occipital condyle, as in most other allosauroids. In *Monolophosaurus*, the paroccipital process extends only slightly below the occipital condyle [72]. The foramen magnum opens posteroventrally in *Acrocanthosaurus* and is surrounded by sharp, raised lateral rims that extend posteroventrally onto the occipital condyle (Figure 15). *Carcharodontosaurus* and *Giganotosaurus* have similarly-pronounced dorsal rims of the foramen magnum [75]. The sharp foramen magnum rim in *Acrocanthosaurus* contrasts with the smoother, more rounded rims of *Allosaurus* and *Sinraptor*. Internal to the foramen magnum, exits for the hypoglossal nerves (XII) perforate the lateral walls of the endocranium.

A computed tomographic (CT) scan of the braincase of NCSM 14345 generated an endocranial endocast that permitted reconstructions of the gross morphology of the brain and its surrounding soft tissue, nerve and blood vessel openings, pituitary fossa, and semicircular canals. Endocast material from other large theropods (*e.g.*, *Allosaurus* [92], [94], *Carcharodontosaurus* [93], [95], *Majungasaurus* [80], *Tyrannosaurus* [90]), including an endocast from the holotype specimen of *Acrocanthosaurus* [91], have contributed a growing collection of digital data to be compared with the endocast of NCSM 14345.

The canal containing the first cranial nerve (I) of *Acrocanthosaurus* is oriented anteriorly, spanning the area between the cerebrum and the olfactory bulbs (Figures 17, 18). The relative length of the canal containing the first cranial nerve of *Acrocanthosaurus* is similar to that seen in *Allosaurus* and *Carcharodontosaurus*, but is shorter than the elongated first cranial nerve of *Majungasaurus*. Posteriorly, the cerebrum exhibits a dorsal crest (‘sinus sagittalis dorsalis’ [91]) anterior to its contact with the cerebellum. On the posterior slope of this crest, a thin opening is reconstructed for the vena capita dorsalis.

The canals containing the optic (II) and oculomotor (III) nerves were not reconstructed from the endocast due to breakage of the braincase. However, a small, rounded protuberance ventral to the trochlear nerve (IV) represents the area from which these nerves exited the braincase, supported by the reconstruction of these nerve exits from the endocast of the holotype specimen [91]. The position of the canal containing the trochlear nerve in *Acrocanthosaurus* is dorsal and slightly anterior to the pituitary fossa; a trochlear nerve is reconstructed in a similar position in *Carcharodontosaurus* [93], although the nerve exit is shown as slightly posterior to the pituitary region in this taxon. Ventral to the pituitary region, the canal containing the narrow abducens nerve (VI) projects anteriorly from the endocast to overlap the sides of the pituitary fossa [91]. The anterior region of the pituitary fossa could not be reconstructed due to missing braincase material.

Posterior to the abducens nerve and pituitary fossa, a large, rounded exit for the trigeminal nerve (V) projects laterally from the cerebellum and exhibits a slight ventral deflection (Figures 17, 18). Two additional openings near the trigeminal nerve are reconstructed on the left side of the endocast and likely represent the canals for the fossa acustico-facialis and the vagus foramen (X). In *Acrocanthosaurus*, the flocculus extends posteroventrolaterally from the endocast, as in *Carcharodontosaurus* and *Allosaurus* [91]. The location of the flocculus is reconstructed posterodorsally to the canal for the trigeminal nerve (Figure 18); this location differs slightly from that of the holotype specimen (and endocasts from specimens of *Allosaurus* and *Carcharodontosaurus*), in which the flocculus is directly posterior to the trigeminal nerve.

The right flocculus reconstructed from the endocranial endocast is more complete than the left, but only a small portion of the right semicircular canals is able to be reconstructed (Figures 17, 18). The left semicircular canals are nearly intact. The anterior semicircular canal is angled posterodorsally. At its juncture with the posterior semicircular canal, a small section of the ventrally-oriented crus commune is oriented dorsoventrally (Figure 17). The posterior semicircular canal slopes posteroventrally to meet the horizontal semicircular canal. The orientation of the horizontal semicircular canal suggests the preferred head posture of *Acrocanthosaurus* is slightly downturned (see [96]), as shown in the lateral view of the skull in Figure 2. The horizontal semicircular canal meets the anteroventral margin of the anterior semicircular canal. The sub-triangular shape and positioning of the semicircular canals in NCSM 14345 closely resemble those described for the holotype specimen of *Acrocanthosaurus* [91], as well as an endocast of *Carcharodontosaurus* [93]. In *Allosaurus*, the angle between the anterior and posterior semicircular canals is more acute [92], giving the canals a more pointed appearance. The hypoglossal nerve (XII) was not reconstructed due to crushing of the braincase.

### Pterygoid

The palatal complex of *Acrocanthosaurus* was known previously from a right palatine and ectopterygoid of SMU 74646 and a partial right ectopterygoid from the holotype specimen. Although the palate of NCSM 14345 was obscured by matrix during the original description of the specimen [1], preparation has since revealed a nearly complete palatal complex (Figure 19). Only the right epipterygoid and a broken portion of the quadrate ramus of the right ectopterygoid are missing in NCSM 14345.

The pterygoid is the longest bone in the palatal complex of *Acrocanthosaurus*, measuring approximately 61 cm from the anterior tip of the vomeropalatine ramus to the posterior margin of the quadrate ramus (Figures 19, 23, 24). Posteriorly, an elongated interpterygoid vacuity is positioned along the midline of the skull, separating each pterygoid from its counterpart (Figure 19). The laterally compressed vomeropalatine ramus is expanded anteriorly from the main body of the pterygoid. The lateral surface of the ramus contacts the medial surface of the vomeropterygoid process of the palatine (Figures 21, 24). In lateral view, the pterygoid is limited in exposure at its contact with the palatine. The vomeropalatine ramus extends further anteriorly in ventral view than in dorsal view. The anterior tip of the vomeropalatine ramus slots between the branched posterior stem of the vomer (Figures 22, 24) and contacts its counterpart medially in ventral view (Figure 23), as in *Allosaurus* [16], [19]. This kinetic region of the palatal complex of *Acrocanthosaurus* resembles that of *Sinraptor*, although it differs slightly from the condition in *Sinraptor* in which vomeropalatine rami may have extended anteriorly past the split section of the vomer [16].

**Figure 20 pone-0017932-g020:**
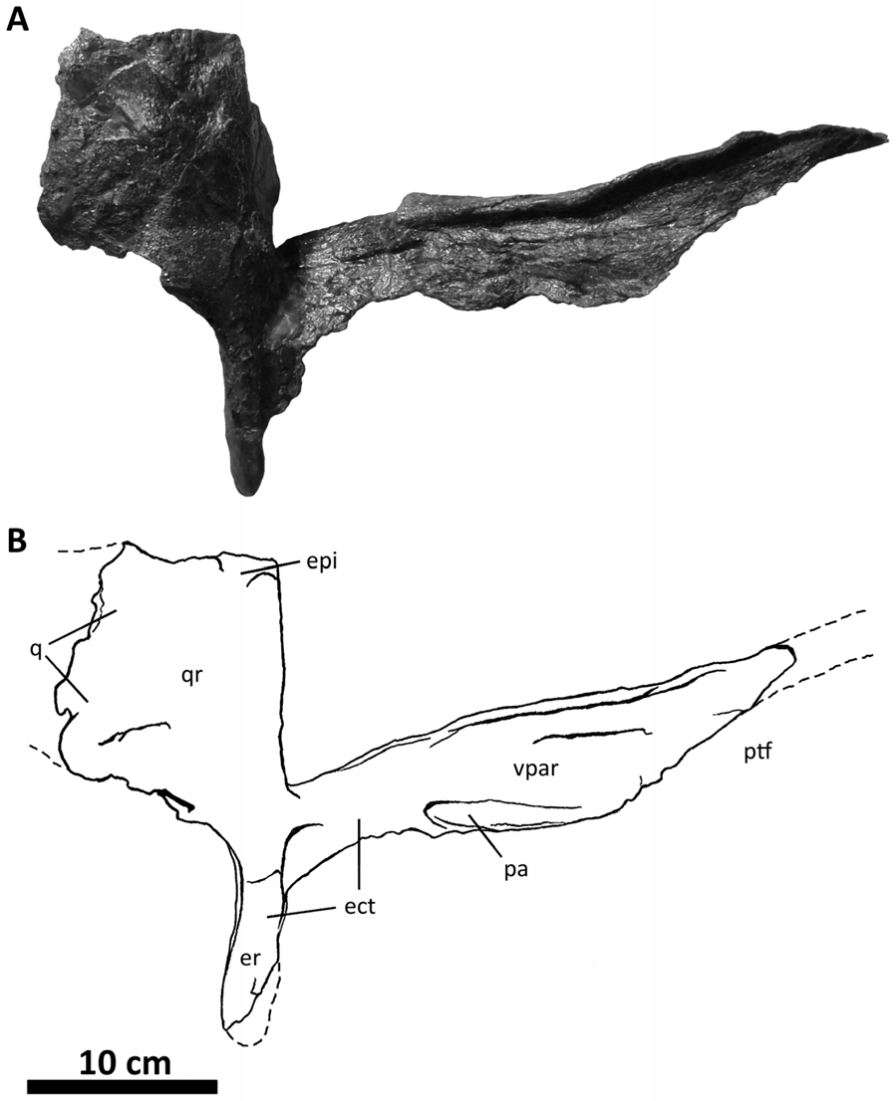
Right pterygoid of *Acrocanthosaurus atokensis* (NCSM 14345) in lateral view. Dashed lines represent material not in figure. **ect**, ectopterygoid contact; **er**, ectopterygoid ramus of pterygoid; **epi**, epipterygoid contact; **pa**, palatal contact; **ptf**; pterygopalatine fenestra; **q**, quadrate contact; **qr**; quadrate ramus of pterygoid; **vpar**, vomeropalatine ramus of pterygoid.

The short, blade-like ectopterygoid ramus of the pterygoid extends ventrally to contact the medial surface of the ectopterygoid and the ectopterygoid pneumatic recess (Figure 20). Medially, a large fossa penetrates the ectopterygoid ramus and extends dorsally into the medial pterygoid process (Figure 46). Similarly-positioned fossae are also present in *Sinraptor* [16], but these depressions are absent in the pterygoids of *Giganotosaurus*, *Allosaurus*, and *Majungasaurus*. The quadrate ramus of the pterygoid is tall and broad, and anterolaterally contacts the epipterygoid. The posterior margin of the pterygoid wing of the quadrate contacts the quadrate ramus of the pterygoid (Figure 20). The basipterygoid process of the basisphenoid articulates with the pterygoid between the quadrate ramus and the pterygoid medial process. Lateral to this contact with the basipterygoid, a deep, posteroventrally-sloping fossa is developed on the medial side of the quadrate ramus (Figure 24) and probably communicated with the fossa of the ectopterygoid ramus. This fossa is also present in the quadrate ramus of *Sinraptor* [16], but is absent in *Allosaurus* and *Giganotosaurus*. The pterygoid medial process (Figures 19, 24) is expressed as a rounded flange of bone that projects posteriorly or posteroventrally in many theropods (*e.g.*, *Allosaurus*, *Ceratosaurus*, *Majungasaurus*, *Tyrannosaurus*), confluent with the angle of the vomeropalatine ramus. However, in *Acrocanthosaurus* and *Sinraptor*, the flange is angled posterodorsally (Figure 46).

### Palatine

**Figure 21 pone-0017932-g021:**
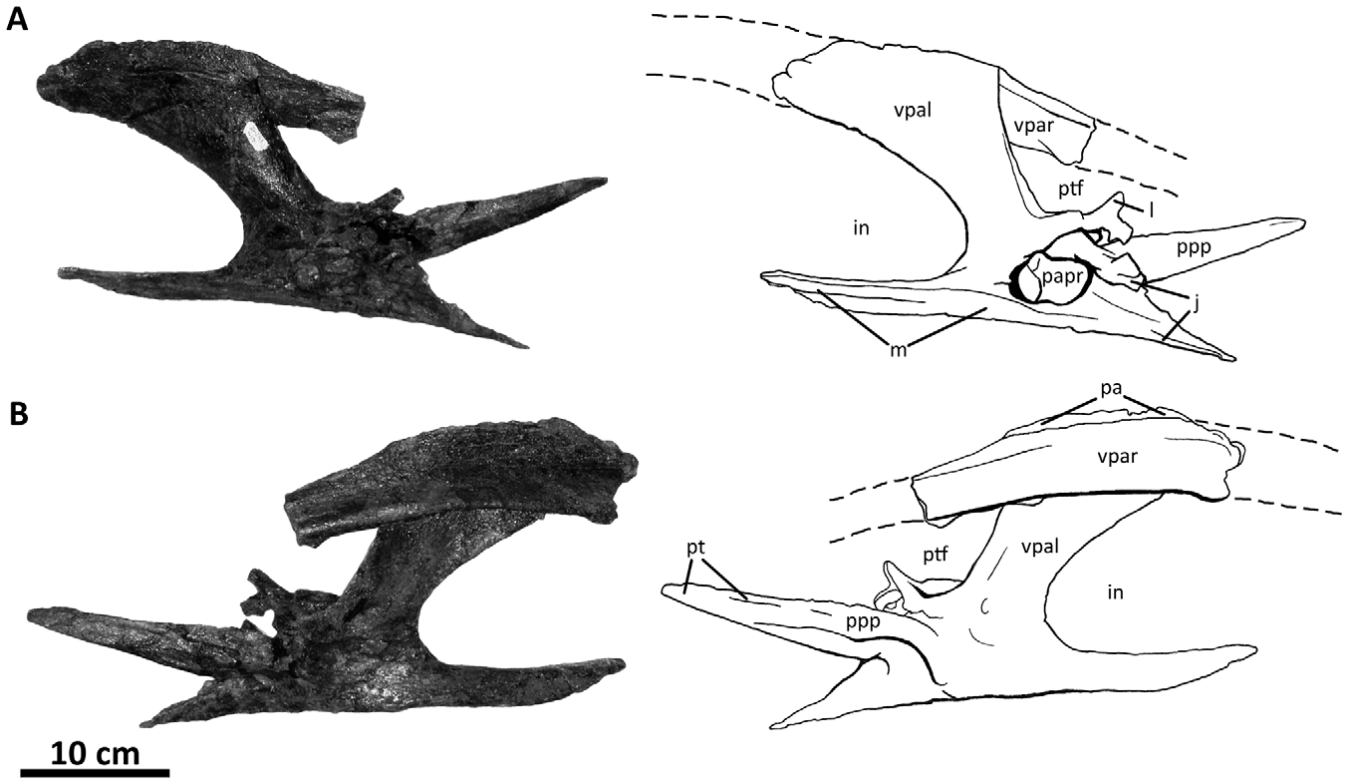
Left palatine of *Acrocanthosaurus atokensis* (NCSM 14345). Palatine in (A) lateral and (B) medial views. Dashed lines represent anterior extension of vomer and posterior extension of vomropalatine ramus of pterygoid. **in**, internal naris; **j**, jugal contact; **l**, lacrimal contact; **m**, maxillary contact; **pa**, palatine contact; **papr**, palatine pneumatic recess; **pt**, pterygoid contact; **ptf**, pterygopalatine fenestra; **ppp**, pterygoid process of palatine; **vpal**, vomeropterygoid ramus of the palatine; **vpar**, vomeropalatine ramus of the pterygoid.

**Figure 22 pone-0017932-g022:**
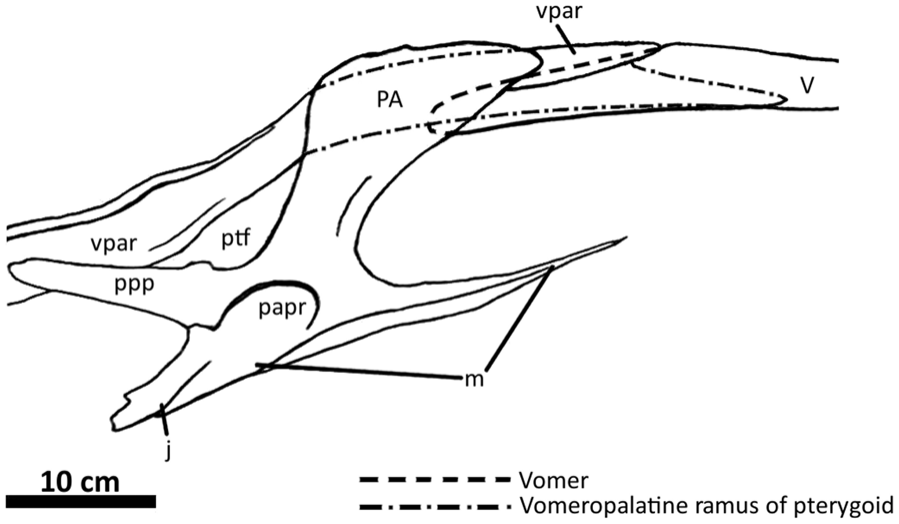
Reconstruction of right pterygoid, palatine, and vomer of *Acrocanthosaurus atokensis* (NCSM 14345) in lateral view. Dashed lines represent hidden surfaces inferred by observing medial and ventral surfaces of shown elements. **m**, maxillary contact; **j**, jugal contact; **PA**, palatine; **papr**, palatine pneumatic recess; **ptf**, pterygopalatine fenestra; **ppp**, pterygoid process of palatine; **V**, vomer; **vpar**, vomeropalatine ramus of pterygoid.

The left and right palatines of NCSM 14345 (Figures 19, 21, 22, 24) are similar in morphology to the right palatine of SMU 74646. Although the palatine of SMU 74646 is relatively complete, the dorsal portion of the vomeropterygoid ramus is broken [21]. Additionally, the internal naris of SMU 74646 is reduced in height, and the pterygoid process is more ventrally deflected in comparison to the palatines of NCSM 14345.

The palatine of *Acrocanthosaurus* is a complex bone comprising four major processes that brace the palatal complex against the facial skeleton along with the ectopterygoid [21], [80]. In lateral view, anteroposteriorly-oriented processes form the ventrolateral margin of the palatine (Figure 21A). Anteriorly, the maxillary process of the palatine is marked by longitudinal ridges that articulate with the medial surface of the posterior ramus of the maxilla. A second palatal process contacts the medial surface of the jugal posteriorly. A large pneumatic recess (∼4.6 cm wide) is developed within the main body of the lateral surface of the palatine of *Acrocanthosaurus* [21] as in *Neovenator* and *Sinraptor* [71], [75], but unlike the apneumatic palatine of *Allosaurus*. The posterodorsal edge of the palatine pneumatic recess is poorly defined by a two-pronged flange that contacts the jugal and lacrimal. Medial to this flange, the blade-like, tapering pterygoid process of the palatine contacts the lateral edge of the vomeropalatine process of the pterygoid and forms the ventral margin of the pterygopalatine fenestra (Figures 22, 24). This fenestra also occurs in *Allosaurus*, *Neovenator*, *Tyrannosaurus*, and *Yangchuanosaurus* [27], [45], [69], but is absent in *Sinraptor* [16].

**Figure 23 pone-0017932-g023:**
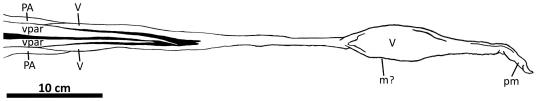
Vomer of *Acrocanthosaurus atokensis* (NCSM 14345) in ventral view. **m**, maxillary contact; **PA**, palatine; **pm**, premaxillary contact; **V**, vomer; **vpar**, vomeropalatine ramus of pterygoid.

The pendulum-shaped vomeropterygoid ramus is the largest of the four palatal processes in *Acrocanthosaurus* and is expanded anterodorsally from the main body of the palatine to form the posterior margin of the internal naris (Figure 21). The lateral surface of the vomeropalatine ramus is slightly rugose at its attachment site for the *M. pterygoidus*, *pars dorsalis* [84]. The medial surface of the ramus contacts the lateral surface of the vomeropalatine process of the pterygoid extensively (Figures 20–24). The palatines meet medially with a narrow symphysis dorsal to their contact with the vomeropalatine processes of the pterygoid (Figure 19). Ventromedial contact with the posterior of the vomer is reduced (Figures 22, 23).

### Vomer

**Figure 24 pone-0017932-g024:**
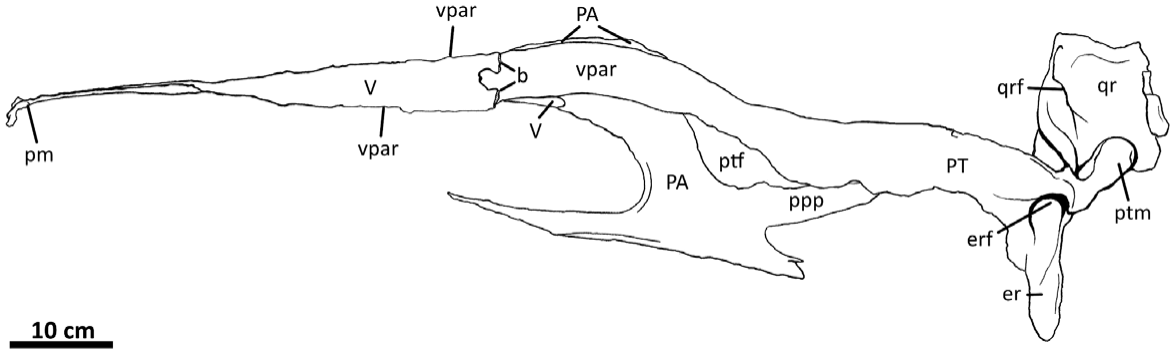
Right pterygoid, palatine, and vomer of *Acrocanthosaurus atokensis* (NCSM 14345) in medial view. Left vomer is figured until it reaches anterior extent of the right palatine. **b**, break; **er**, ectopterygoid ramus of the pterygoid; **erf**, fossa of the ectopterygoid ramus of the pterygoid; **PA**, palatine; **pm**, premaxillary contact; **PT**, pterygoid; **ptm**, pterygoid medial process; **ptf**, pterygopalatine fenestra; **ppp**; pterygoid process of palatine; **qr**, quadrate ramus of pterygoid; **qrf**; fossa of the quadrate ramus of the pterygoid; **V**, vomer; **vpar**, vomeropalatine ramus of the pterygoid.

The only vomeral material referred to *Acrocanthosaurus* comes from NCSM 14345. Anteriorly, the elongated vomer (46.1 cm in length) contacts the medial symphysis of the premaxilla with a short, tapering process that is ventrally deflected (Figures 19, 23, 24). Posterior to its contact with the premaxilla, the vomer flattens dorsoventrally and widens laterally (‘rhomboid flange’ [97]) near a possible contact point with the anteromedial processes of the maxillae, as in *Sinraptor* and *Tyrannosaurus*. Further posteriorly, the lateromedially-compressed ‘posterior stem’ of the vomer [97] is expanded dorsoventrally near its contact with the palatine (Figure 24). Anterior to this contact, the posterior stem is split along its midline into left and right processes by a deep sulcus that extends further anteriorly in ventral view than in dorsal view (Figures 19, 23).

Anterior to the palatine-vomer contact, the vomeropalatine rami of the pterygoid overlap the vomer laterally (Figures 19, 22). Unlike in *Sinraptor*, ventral troughs for contact with the pterygoid are not visible on the vomer of *Acrocanthosaurus*. *Acrocanthosaurus* shares the plesiomorphic condition of the vomer not contacting the pterygoids with *Sinraptor* and the coelurosaur *Tyrannosaurus* [97], but unlike *Allosaurus* in which the two elements contact each other [16], [98]. In ventral view, the vomeropalatine rami of the pterygoids are visible within the vomeral sulcus and are overlapped by the medial surfaces of the split posterior stem of the vomer. The vomer terminates posteriorly and is overlapped by the medial surfaces of the vomeropterygoid processes of the palatines (Figure 22), a condition also described in the palate of *Tyrannosaurus* [97]. A similar arrangement of palatal elements occurs in *Sinraptor* [16], although this taxon preserves dorsal troughs on the vomer that are not visible in *Acrocanthosaurus* due to palatine-vomer fusion.

### Ectopterygoid

Ectopterygoid material was previously referred to *Acrocanthosaurus*, including a partial right ectopterygoid from the holotype specimen [23] and a right ectopterygoid from SMU 74646 [21]. The ectopterygoid from SMU 74646 was mislabeled as a left element, but a suture on the medial surface of the right jugal articulates with the jugal ramus of the ectopterygoid, confirming its identification as a right element. The well-preserved ectopterygoids of NCSM 14345 are morphologically similar to those referred to both the holotype specimen and SMU 74646, but were not visible during the description by Currie and Carpenter [1].

**Figure 25 pone-0017932-g025:**
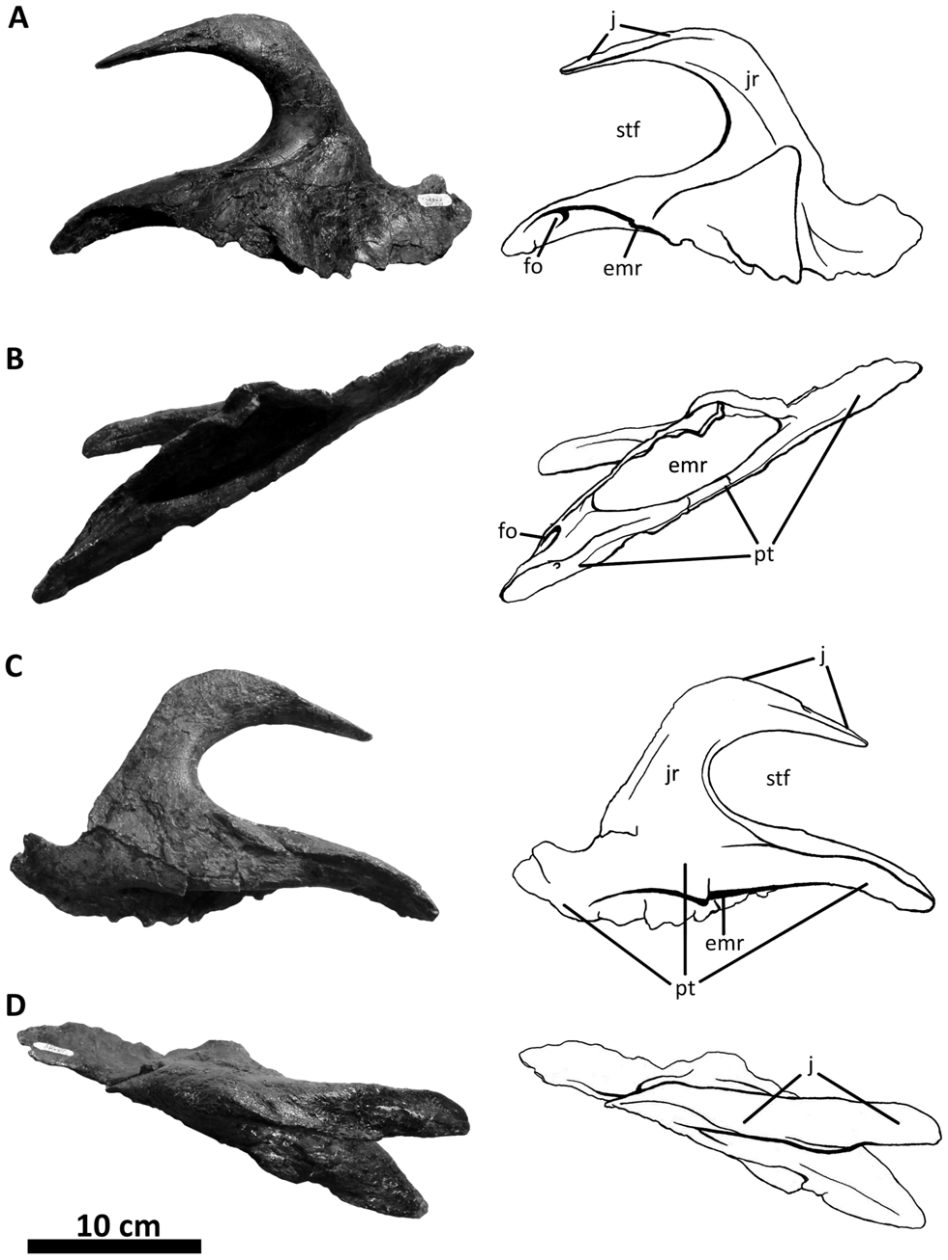
Left ectopterygoid of *Acrocanthosaurus atokensis* (NCSM 14345). Ectopterygoid in (A) dorsal, (B) medial, (C) ventral, and (D) lateral views. **emr**, ectopterygoid medial recess; **fo**, foramen; **j**, jugal contact; **jr**, jugal ramus of ectopterygoid; **pt**, pterygoid contact; **stf**, subtemporal fenestra.

The ectopterygoid of *Acrocanthosaurus* is hook-shaped in dorsal view [21], and the jugal ramus extends posterolaterally from the medial body of the bone to contact the medial surface of the jugal (Figure 25). In *Allosaurus*, *Sinraptor*, and the megalosauroid taxon *Dubreuillosaurus valesdunensis* Allain 2002 [56], the ectopterygoid contacts the jugal with an expanded, triangular ramus (Figure 47). In these taxa, the angle of the jugal ramus also parallels the ventral margin of the main body of the ectopterygoid. In *Acrocanthosaurus*, *Giganotosaurus*, and a probable partial ectopterygoid from *Carcharodontosaurus* (SGM-Din 1), the jugal ramus is rectangular in lateral view, with parallel dorsal and ventral margins. In *Acrocanthosaurus* and *Giganotosaurus* the ramus is inclined dorsally by approximately twenty degrees to the ventral margin of the ectopterygoid (Figure 47).

The medial surface of the ectopterygoid extensively contacts the pterygoid between the vomeropalatine and ectopterygoid rami (Figure 25B). The ectopterygoid is perforated medially by an elongate, ovular vacuity within the main body of the element that expands into the base of the jugal ramus, unlike the sub-circular ectopterygoid recess of *Tyrannosaurus* [99]. The ectopterygoid of *Acrocanthosaurus* is also characterized by small fossae along the medial edge of the main body, positioned posterior to the medial vacuity. These fossae are not preserved in either the holotype specimen or SMU 74646, but occur in both left and right ectopterygoids of NCSM 14345. The fossae open medially and are in close association with the fossae that perforate the ectopterygoid ramus of the pterygoid. *Giganotosaurus* preserves two accessory fossae on the medial surface of the ectopterygoid, whereas *Allosaurus* and *Sinraptor* preserve none.

### Epipterygoid

**Figure 26 pone-0017932-g026:**
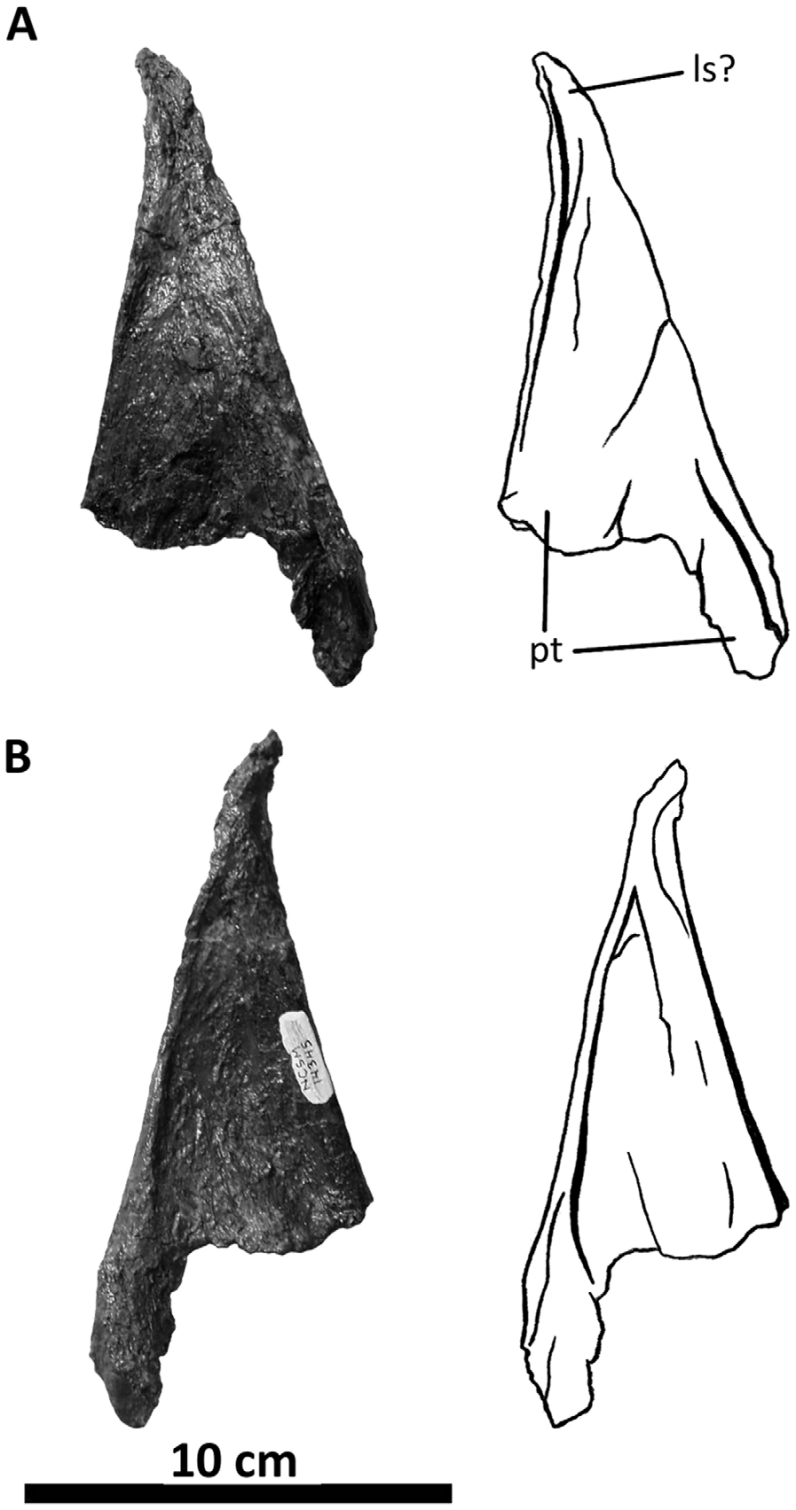
Left epipterygoid of *Acrocanthosaurus atokensis* (NCSM 14345). Epipterygoid in (A) lateral and (B) medial views. **ls**, laterosphenoid contact; **pt**, pterygoid contact.

The left epipterygoid of NCSM 14345 is the only such element currently referred to *Acrocanthosaurus*. The triangular epipterygoid is laterally compressed and overlaps the lateral surface of the quadrate ramus of the pterygoid (Figures 19, 26). In medial view, a thin ridge is expands posteriorly along the anteromedial margin of the epipterygoid. Ventral to this ridge, a short, rounded process overlaps the pterygoid. Dorsally, the epipterygoid tapers to a point as in *Ceratosaurus magnicornis* Madsen and Welles 2000 [81], *Cryolophosaurus ellioti* Hammer and Hickerson 1994 [100], and some tyrannosauroids [101]. In contrast, some specimens of *Allosaurus* (UUVP 1414; BYU 671/8901) [102] and *Tyrannosaurus* (FMNH PR2081) [82] have a wide, bulbous dorsal tip of the epipterygoid (Figure 48).

Dorsally, the epipterygoid of *Acrocanthosaurus* approaches, but does not articulate with the laterosphenoid; this contact is present in other theropods (*e.g.*, *Tyrannosaurus* [82], *Cryolophosaurus* [13]). In an articulated skull of *Allosaurus* (BYU 571/8901) [102], an undescribed epipterygoid articulates with the pterygoid and is in close proximity to the laterosphenoid, but does not contact the braincase. This is inconsistent with the interpretation [69] of a highly kinetic epipterygoid-laterosphenoid contact in *Allosaurus*. However, absence of epipterygoid-laterosphenoid contact in *Allosaurus* and *Acrocanthosaurus* supports the model proposed by Holliday and Witmer of a reduction in the size and kinetic nature of the epipterygoid in non-avian theropods [103].

### Dentary

**Figure 27 pone-0017932-g027:**
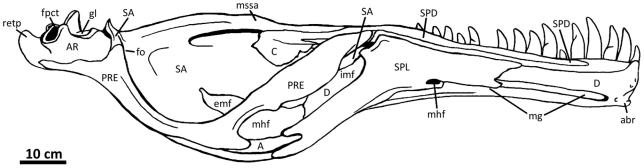
Left mandible of *Acrocanthosaurus atokensis* (NCSM 14345) in medial view. **A**, angular; **AR**, articular; **abr**, articular brace of the dentary; **C**, coronoid; **D**, dentary; **emf**, external mandibular fenestra; **fo**, foramen; **fpct**, foramen posterior chorda tympani; **gl**, glenoid region; **imf**, internal mandibular fenestra; **mg**, Meckelian groove; **mhf**, mylohyoid foramen; **mssa**, medial shelf of the surangular; **PRE**, prearticular; **retp**, retroarticular process of articular; **SA**, surangular; **SPD**, supradentary; **SPL**, splenial.

**Figure 28 pone-0017932-g028:**
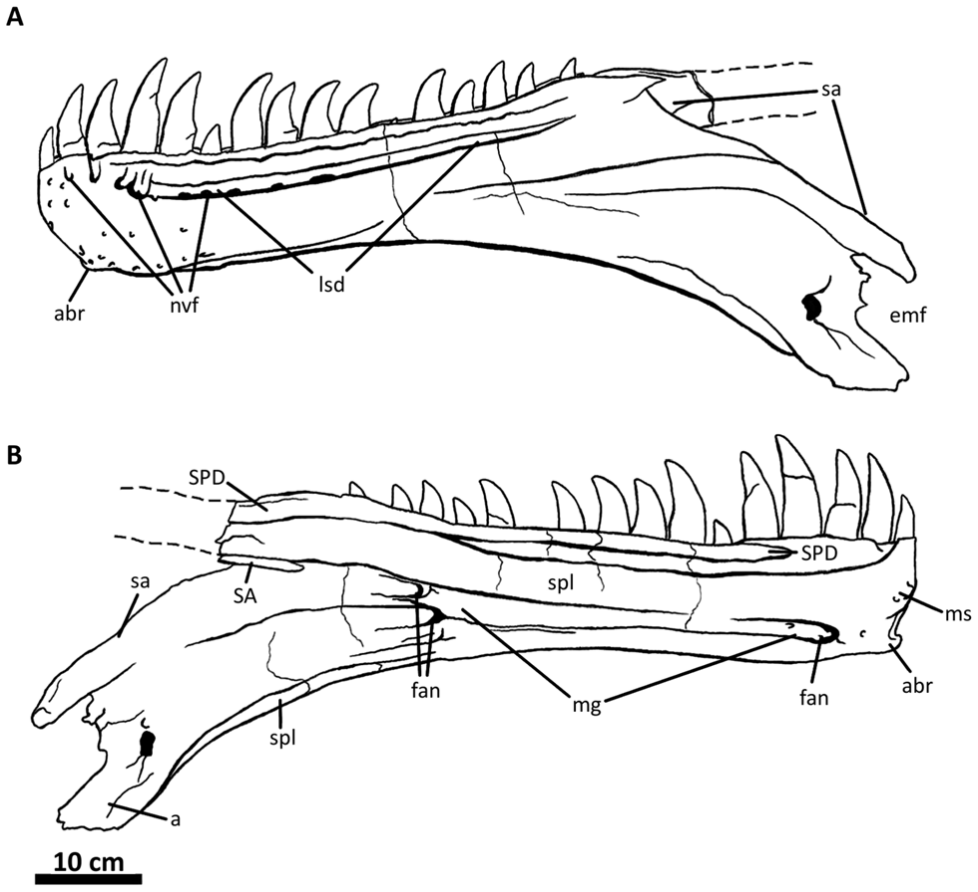
Left dentary of *Acrocanthosaurus atokensis* (NCSM 14345). Dentary in (A) lateral and (B) medial views. Dashed lines represent material not in figure. **a**, angular contact; **abr**, articular brace of the dentary; **emf**, external mandibular fenestra; **fan**, foramina of the inferior alveolar nerve; **lsd**, lateral sulcus of the dentary; **mg**, Meckelian groove; **ms**, medial symphysis; **nvf**, neurovascular foramina; **SA**, surangular; **sa**, surangular contact; **SPD**, supradentary; **spl**, splenial contact.

Only the lateral surface of the mandible, including the dentary of NCSM 14345, was described by Currie and Carpenter [1]. The mandible is complete except for a missing right coronoid. The dentary (Figures 2, 27, 28) is a long (82.1 cm), lateromedially compressed element (4.2 cm in width) that preserves 17 alveoli [1]. In dorsal view the dentary is relatively straight, as in *Australovenator*, *Neovenator*, and *Sinraptor* [16], [48], [75], but a slight anteromedial curvature occurs at the anterior region in *Acrocanthosaurus* as in other allosauroids. The number of dentary alveoli varies from 14 to 17 in specimens of *Allosaurus*, with a mean number of 16 [69]. Dentaries of a specimen of *Monolophosaurus* preserve 17 and 18 alveoli, whereas *Sinraptor* has 16 alveoli per dentary [16], [71].

In lateral view, the posterior margin of the dentary overlaps the surangular and angular (Figures 2, 28A). The intramandibular joint describes the region where the posteriorly-projecting lateral process of the dentary overlaps the surangular [1]. Ventral to this joint, the intramandibular process of the surangular narrowly overlaps the lateral surface of the dentary. This process also occurs in *Sinraptor*, but is reduced in *Monolophosaurus*, *Allosaurus*, and *Yangchuanosaurus*. The dentary of *Acrocanthosaurus* contacts the surangular posteroventrally from the intramandibular joint. The dentary is bifurcated posteriorly and forms the anterior margin of the external mandibular fenestra (Figure 28A). The upper prong articulates with the surangular; the lower prong overlaps the lateral surface of the anterior portion of the angular [1]. The forked posterior processes of the dentary in *Acrocanthosaurus* are not as pronounced as those of *Sinraptor* and *Yangchuanosaurus*. However, the dentary appears more strongly bifurcated in *Acrocanthosaurus* than in *Monolophosaurus* and *Allosaurus*.

Ventral to the alveolar margin, a deep lateral sulcus extends anteroposteriorly across the dentary surface (Figures 28A, 49C) to accommodate a row of elongated fossae [1]. Additional fossae are expressed past the anterior margin of the sulcus. These depressions are smaller and more rounded than the anteroposteriorly-elongated fossae set within the sulcus of the dentary of *Acrocanthosaurus*, a condition also present in *Mapusaurus* [36] and possibly *Carcharodontosaurus* (MNN IGU5) [75]. In contrast, the anterior region of dentaries referred to *Allosaurus*, *Neovenator*, and *Monolophosaurus* preserve fossae at the surface of the dentary (Figure 49A), not inset within a sulcus. *Sinraptor* displays an intermediate condition: the posterior-most fossae are inset within a sulcus, but the sulcus does not project anteriorly across the dentary (Figure 49B). In *Giganotosaurus*, the lateral sulcus is inset deeply within the posterior portion of the dentary and appears to project forward, but damage has obscured the anterior extent of this groove [104].

Much of the medial surface of the right dentary is obscured by the splenial, although the two elements are disarticulated in the left mandible. The enclosed mylohyoid foramen of the splenial opens internally onto the medial surface of the dentary. The posterior expansion of the Meckelian groove on the dentary is expressed medial to this opening as an elongated, deeply-inset medial sulcus (Figures 27, 28B). The Meckelian groove terminates posteriorly with two crescentic, posteriorly-opening foramina for paired branches of the alveolar nerve. The dorsal foramen is offset posteriorly from the ventral foramen, as in *Sinraptor*. In *Allosaurus*, the two foramina are spaced further apart, often by more than the width of a dentary alveolus. Anteriorly, the Meckelian groove in *Acrocanthosaurus* terminates ventral to the third dentary alveolus, and the alveolar nerve penetrates the dentary with the expression of a large foramen.

The anteroventral margin of the dentary of *Acrocanthosaurus* (Figure 28) is squared in medial and lateral views [1], but slightly less so than in *Giganotosaurus*, *Tyrannotitan*, *Mapusaurus*, and *Carcharodontosaurus* [36], [39], [49]. At its anteromedial surface, the dentary articulates with its counterpart along a short, anteroventral process (‘chin’ [75]). *Acrocanthosaurus* shares the presence of this flange with the four aforementioned carcharodontosaurids. In *Sinraptor*, *Yangchuanosaurus*, *Allosaurus*, and *Neovenator*, the anteroventral margin of the dentary is more rounded, and the articular flange is absent or greatly reduced. Dorsal to this flange in *Acrocanthosaurus*, the groove for the dental lamina is curved posteriorly from its origin at the base of the first dentary tooth and is overlain by the splenial. In dorsal view, the dentary symphysis is V-shaped at its convergence, similar to *Allosaurus* but unlike the higher-angled, U-shaped dentary symphysis of *Carcharodontosaurus* [75].

### Supradentary and Coronoid

The supradentary (‘intercoronoid’ [69]) and coronoid of NCSM 14345 are the only such elements referred to *Acrocanthosaurus* and are described here for the first time in this taxon. In medial view, the thin supradentary process overlaps the dentary immediately ventral to the alveolar margin (Figures 27, 28B). The anterior margin of the supradentary is positioned ventral to the fourth dentary alveolus in *Acrocanthosaurus*, as in *Monolophosaurus*. The lateromedially-flattened supradentary extends past the posterior margin of the dentary alveoli, at which point it displays a posteroventral curvature and narrowly contacts the medial surface of the surangular. The supradentary is fused with the dorsal process of the coronoid (Figures 27, 32B). Supradentary-coronoid continuity has been noted in *Monolophosaurus* [71] and some specimens of *Tyrannosaurus* [82]. This continuity may be more broadly distributed within Theropoda, but because coronoid and supradentary elements are prone to disarticulation due to their ligamentous attachment to the mandible, such fusion is not commonly preserved [82]. Propensity for this disarticulation in allosauroids is supported by the presence of isolated supradentaries and coronoids in specimens of *Allosaurus* [69], and the lack of these elements in an otherwise nearly complete skull of *Sinraptor* (IVPP 10600).

The coronoid of *Acrocanthosaurus* is lateromedially-flattened like the supradentary, sub-rectangular, and split anteriorly by a narrow sulcus that separates the element into dorsal and ventral processes. As previously mentioned, the dorsal process is continuous with the supradentary; the ventral process of the coronoid tapers to a point and dorsomedially overlaps an anteriorly-projecting process of the surangular (Figure 32B). Although the supradentaries of *Monolophosaurus*, *Tyrannosaurus*, and *Allosaurus* are similar in morphology to that of *Acrocanthosaurus*, coronoids from these taxa appear triangular in medial view and display a tapering posterodorsal flange that is absent in *Acrocanthosaurus*. Coronoid material has not been described for *Sinraptor*, *Yangchuanosaurus*, or taxa within Carcharodontosauria, and therefore the extent of morphological variation of the coronoid within Allosauroidea is uncertain.

### Splenial

Splenial material previously referred to *Acrocanthosaurus* (SMU 74646) [21] is highly fragmentary, and its identification as a splenial is equivocal. Both splenials of NCSM 14345 are well preserved but were not visible during the original description of the specimen [1]. The right splenial is mounted in articulation with the dentary, obscuring its internal surface, although the left splenial is isolated from the mandible.

**Figure 29 pone-0017932-g029:**
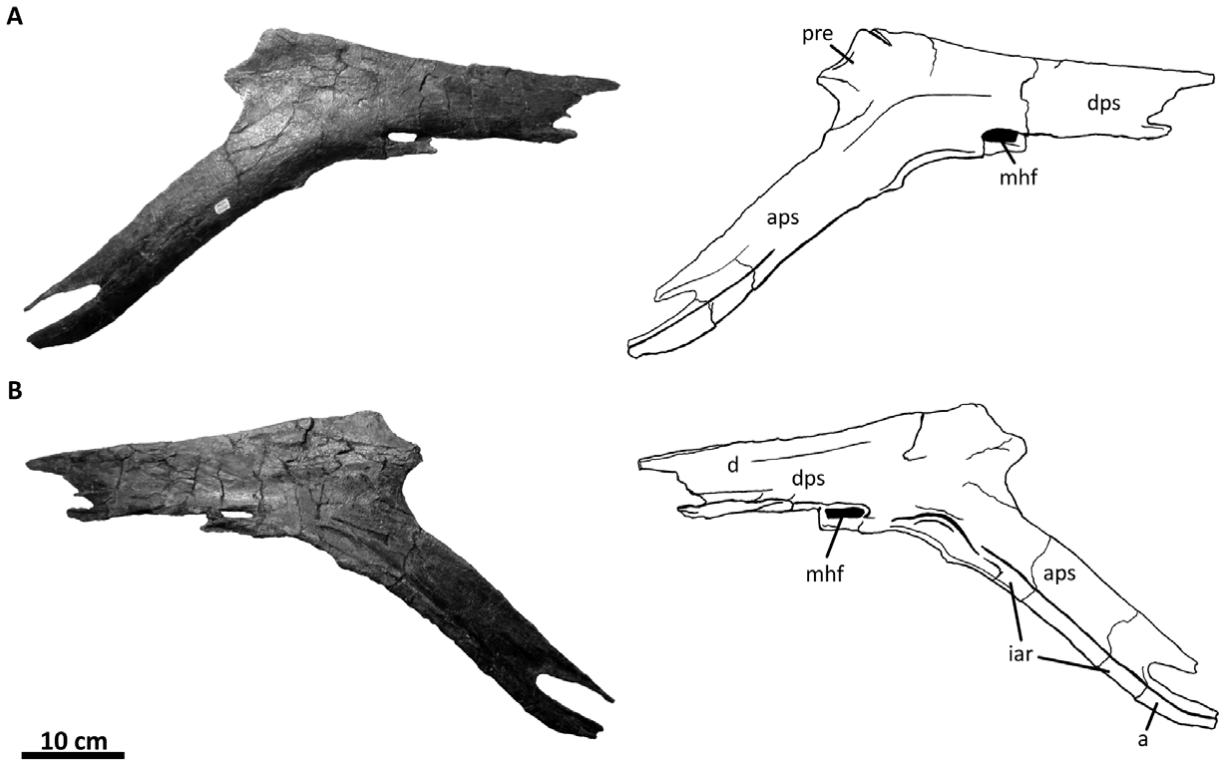
Left splenial of *Acrocanthosaurus atokensis* (NCSM 14345). Splenial in (A) medial and (B) internal views. **a**, angular contact; **aps**, angular process of the splenial; **d**, dentary contact; **dps**, dentary process of the splenial; **iar**, infra-angular ridge; **mhf**, mylohyoid foramen; **pre**, prearticular contact.

The splenial is a long (∼60.5 cm), medially convex sheet of bone that articulates with the medial surface of the dentary anteriorly and the articular posteriorly (Figures 27, 29). The ventral margin of the splenial is curved posteroventrally in *Acrocanthosaurus*, *Mapusaurus*, and *Allosaurus*; in *Sinraptor*, *Yangchuanosaurus*, and *Monolophosaurus*, this ventral margin is straight. The dentary process of the splenial is forked anteriorly into separate prongs in *Acrocanthosaurus*. The dorsal prong of the splenial terminates below the eighth dentary alveolus. The smaller ventral prong does not project as far anteriorly and ends below the ninth alveolus (Figure 27). Along the ventral margin, the anteroposteriorly-elongated anterior mylohyoid foramen is completely enclosed by the splenial (Figure 29). The splenial also entirely encloses the mylohyoid foramen in *Mapusaurus*, *Sinraptor*, and *Tyrannosaurus*. In *Allosaurus*, *Monolophosaurus*, and some non-allosauroid theropods (*e.g.*, *Dubreuillosaurus* , *Majungasaurus*) this anterior mylohyoid foramen is present, but its anterior margin is open and not surrounded by the splenial.

Posterodorsally, a squared splenial process contacts the prearticular of *Acrocanthosaurus*. The splenial surface that contacts the rounded tip of the prearticular is slightly concave (Figures 27, 29), and proportionally shorter than the posterodorsal projections of the splenial in *Allosaurus*, *Monolophosaurus*, *Sinraptor*, and *Tyrannosaurus*. *Acrocanthosaurus* shares a short posterodorsal projection of the splenial with *Mapusaurus*. In *Acrocanthosaurus*, the posteroventrally-downturned angular process of the splenial contacts and parallels the posteroventral curvature of the dentary. Internally, an infra-angular ridge is developed along the posteroventral margin of the splenial and contacts the dentary and angular. This ridge is relatively thin in *Acrocanthosaurus*, *Sinraptor*, and *Allosaurus*, compared to the thicker, raised infra-angular ridges of *Ceratosaurus*, *Tyrannosaurus*, and *Mapusaurus*. The shape of the infra-angular ridge is not known in *Giganotosaurus* and *Carcharodontosaurus*, because no splenial material has yet been referred to these taxa.

The posterior margin of the splenial forms the anterior margin of the internal mandibular fenestra (Figure 27; ‘Meckelian fossa’ [82]). This opening is also present between the splenial and prearticular of *Mapusaurus*, *Monolophosaurus*, and *Sinraptor*, but is greatly reduced in *Yangchuanosaurus*. This fenestra in the aforementioned allosauroid taxa is not homologous to the internal mandibular fenestra described for *Majungasaurus* by Sampson and Witmer [80], which is instead attributed to the open region dorsal to the prearticular and ventral to the medial surangular shelf.

### Prearticular

**Figure 30 pone-0017932-g030:**
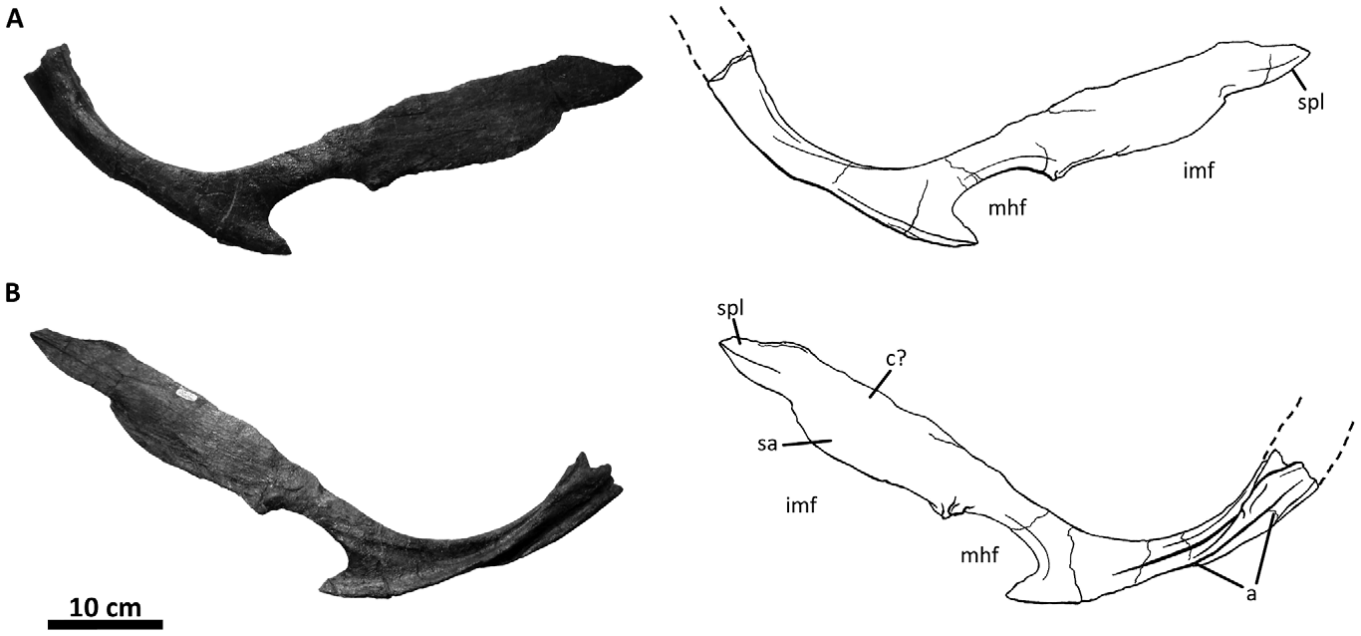
Left prearticular of *Acrocanthosaurus atokensis* (NCSM 14345). Prearticular in (A) medial and (B) internal views. Dashed lines represent material not in figure. **a**, angular contact; **c**, coronoid contact; **imf**, internal mandibular fenestra; **mhf**, mylohyoid foramen; **sa**, surangular contact; **spl**, splenial contact.

Incomplete prearticular material from SMU 74646 and one fragmentary prearticular from the holotype specimen were previously referred to *Acrocanthosaurus* [21], [23]. Both prearticulars are intact in NCSM 14345. This narrow, ventrally-bowed element is exposed along the medial surface of the mandible (Figures 27, 30). Posteriorly, the prearticular expands to broadly underlie the articular and posterior process of the surangular. Anteroventral to this contact, the prearticular is sub-cylindrical as it thickens lateromedially. Here, the prearticular contacts the medial surface of the angular, and the two elements form the ventral margin of the mandible. This region of the mandible is deflected deeply ventrally in *Acrocanthosaurus*, in contrast to more shallow posterior regions of the mandibles of *Allosaurus*, *Monolophosaurus*, *Sinraptor*, and several ceratosaurs [80].

Internally, a series of parallel ridges and depressions ornament the posterior region of the prearticular (Figure 30B). Anterior to these ridges, an elongated (8.55 cm in length) posterior mylohyoid foramen opens anteroventrally into the internal mandibular fenestra (Figure 27). Several theropods do not preserve a posterior mylohyoid fenestra, including *Dubreuillosaurus* , *Majungasaurus*, *Monolophosaurus*, *Sinraptor* (Figure 50A), and *Tyrannosaurus*. However, in *Sinraptor* and *Monolophosaurus*, the prearticular displays a slight concavity along its ventral margin in the approximate region of the posterior mylohyoid foramen. In *Allosaurus*, the posterior mylohyoid foramen (Figure 50B) is proportionally much smaller than in *Acrocanthosaurus* (Figure 50C). Above this opening, the anteroventral margin of the prearticular forms the posterodorsal rim of the internal mandibular fenestra. The prearticular tapers anteriorly as it nears the dorsal margin of the mandible (Figure 27) and may contact the coronoid (Figure 30). The anterior tip of the prearticular contacts the posterodorsal process of the splenial.

### Angular

**Figure 31 pone-0017932-g031:**
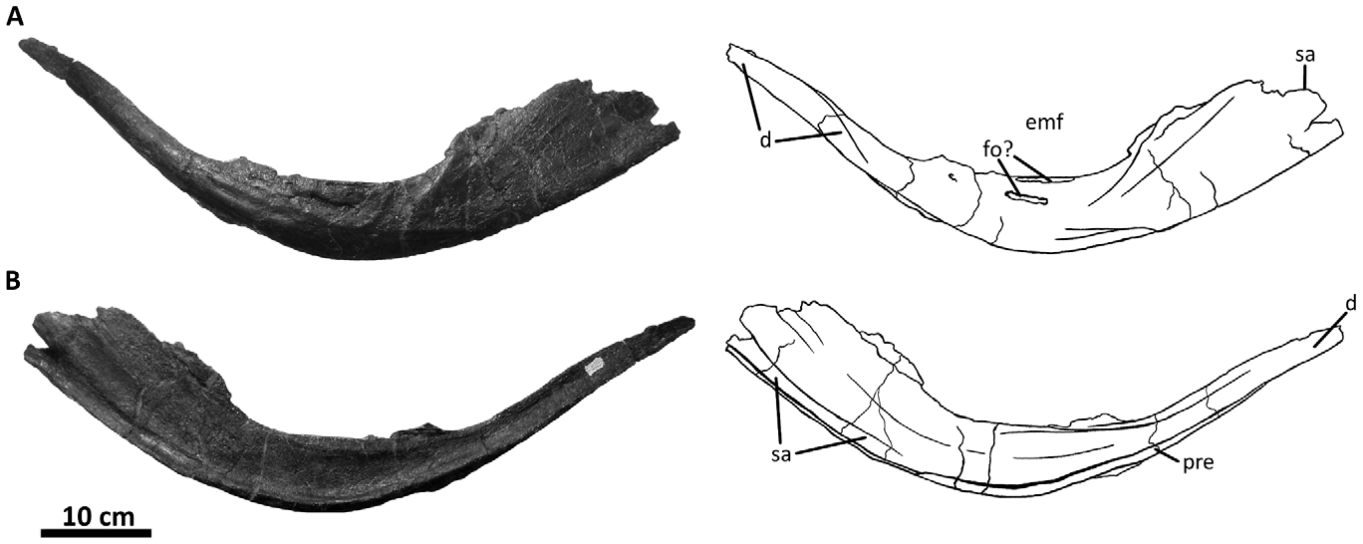
Left angular of *Acrocanthosaurus atokensis* (NCSM 14345). Articular in (A) lateral and (B) medial views. **d**, dentary contact; **emf**, external mandibular fenestra; **fo**, foramina; **pre**, prearticular contact; **sa**, surangular contact.

The lateral surface of the angular has been described for *Acrocanthosaurus* [1]. The intact left and right angulars of NCSM 14345 represent the most complete material referred to the taxon, although a fragmentary angular is recognized from the holotype specimen [23]. The angular is flattened lateromedially and parallels the curvature of the prearticular to form the ventral margin of the mandible (Figures 2, 31). Anteriorly, the angular narrows and curves anterodorsally to contact the medial surface of the dentary and internal surface of the splenial. The angular overlaps the lateral surface of the surangular posteriorly (Figure 2), and contacts the prearticular medially [1]. The medial surface of the angular preserves a thin ridge along its ventral margin that articulates with the medial ridge of the prearticular. The dorsal surface of the angular forms the ventral margin of the external mandibular fenestra. Anteroposteriorly-elongated fossae are visible below the dorsal margin of both angulars. It is unclear whether these depressions are accessory pneumatic structures of the external mandibular fenestra or simply neurovascular foramina. Fossae are absent in the angulars of *Sinraptor*, *Monolophosaurus*, and *Allosaurus*, although angulars referred to *Tyrannosaurus* preserve large openings in this region [82].

### Surangular

**Figure 32:**
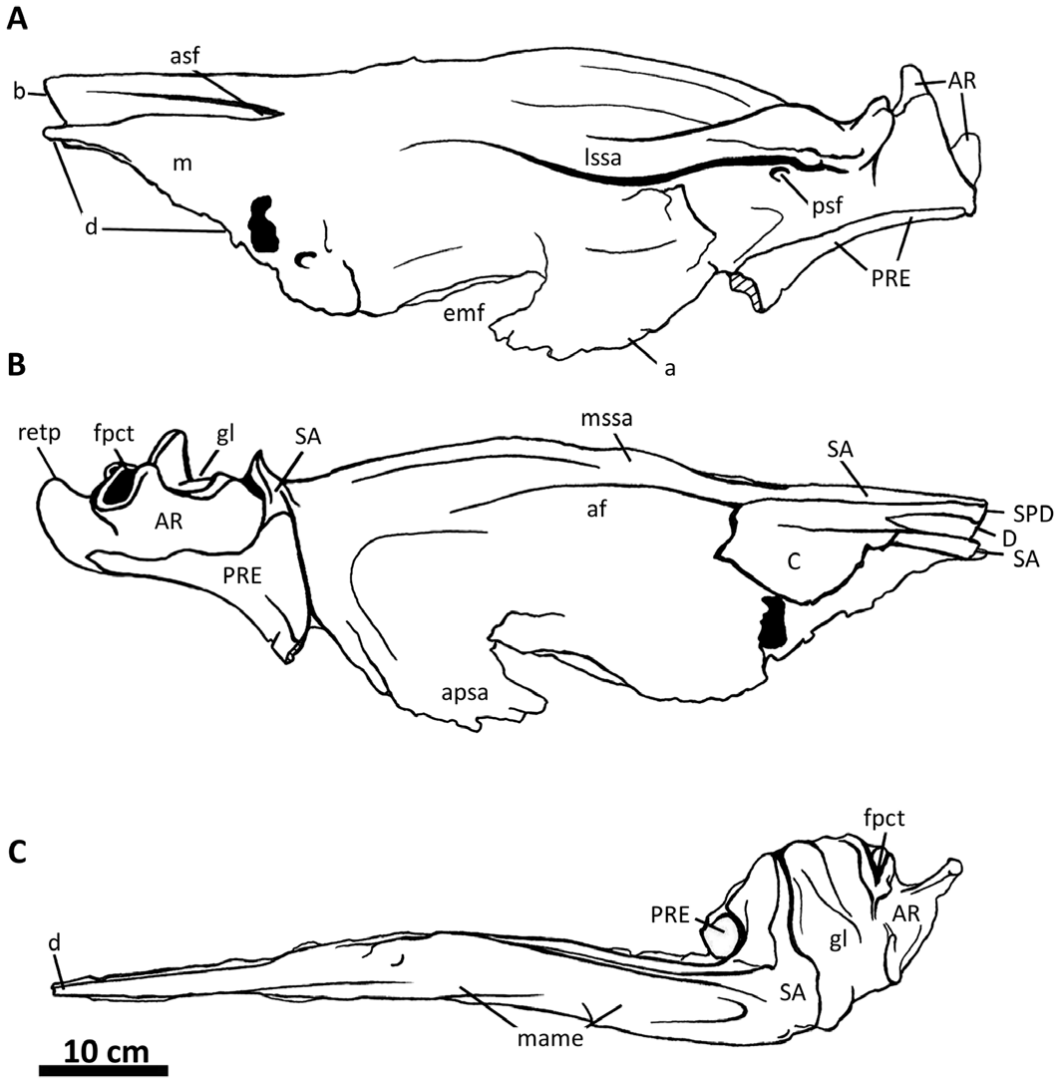
Left surangular, articular, and coronoid of *Acrocanthosaurus atokensis* (NCSM 14345). Surangular, articular, and coronoid in (A) lateral, (B) medial, and (C) dorsal views. Hatched lines represent broken surfaces. **a**, angular contact; **af**, adductor fossa; **apsa**, angular process of surangular; **AR**, articular; **asf**, anterior surangular foramen; **b**, break; **C**, coronoid; **D**, dentary; **d**, dentary contact; **emf**, external mandibular fenestra; **fpct**, foramen posterior chorda tympani; **gl**, glenoid region of articular; **lssa**, lateral shelf of surangular; **m**, maxillary contact (mouth closed); **mame**, insertion for the *M. adductor mandibulae externus*; **mssa**, medial shelf of surangular; **PRE**, prearticular; **psf**, posterior surangular foramen; **retp**, retroarticular process; **SA**, surangular; **SPD**, supradentary.

The surangular, articular, and posterior portion of the prearticular of NCSM 14345 are preserved in articulation (Figure 32), similar to material referred to *Acrocanthosaurus* from the posterior mandible of the holotype specimen and SMU 74646. Incomplete surangular material has been previously described, including a partial left surangular from the holotype and a partial right surangular from SMU 74646. Both surangulars are intact in NCSM 14345, with only the lateral surfaces previously described [1].

The surangular is an elongated element (∼59.1 cm) composed of a lateromedially- flattened anterior sheet of bone bordered dorsally by expanded lateral and medial surangular shelves. The medial shelf of the surangular is positioned further dorsally than the lateral shelf, and the *M. adductor mandibulae externus* presumably attaches dorsally and laterally along a depression between these shelves [80]. Here, the surangular also nears the mass occupied by the quadratojugal, jugal, and the posterior ramus of the maxilla when the mouth of *Acrocanthosaurus* is closed. A knob located near the posterior margin of the lateral surangular shelf of NCSM 14345 [1] is also present in SMU 74646 [21]. Ventral and slightly anterior to this knob, a rounded posterior surangular foramen is visible (Figure 32A). The angular laterally overlaps a flat, anteroventrally-projecting flange of the surangular to form the posterior and ventral margins of the external mandibular fenestra (Figure 2). Anteriorly, two large, irregularly-shaped openings perforate the surangular and the thin posterior process of the dentary (Figures 28, 32A). Similarly-positioned openings in greater abundance are described from the surangular of *Tyrannosaurus* as lesions with surrounding rings of inflated bone [82]. In *Acrocanthosaurus*, the openings exhibit flat margins and are suggested to represent post-depositional damage [1].

The anterior process of the surangular is tall (>16 cm) and contacts the dentary along a posteroventrally-sloped margin (Figure 32C). The anterior tip of the surangular participates in the external mandibular joint (Figures 27, 32A) with a thin, blade-like flange that overlaps the lateral surface of the dentary [1]. The dorsal margin of the flange terminates posteriorly at the entrance for the anterior surangular foramen. In anteromedial view, another large foramen opens anteriorly between the right surangular and the prearticular; this depression is absent in the left prearticular. Dorsal to this foramen, the medial shelf of the surangular splits anteriorly into two processes near lateral contact with the coronoid. The anterior extent of the dorsal process is obscured laterally by the supradentary (Figures 27, 32B); the ventral process contacts the internal surface of the coronoid and extends anteriorly past the mandibular joint to overlap the medial surface of the dentary.

### Articular

Unlike more gracile elements in the mandible, the robust articular of *Acrocanthosaurus* is represented in the holotype specimen, SMU 74646, and NCSM 14345. Anteriorly and ventrally, the articular of NCSM 14345 remains adhered to the surangular and prearticular, obscuring its articular surfaces. Dorsally, the articular is wide (14.25 cm) and possesses a complex sequence of bony ridges and deep furrows that surround a depressed glenoid region [1], [21]. The lateral and medial glenoids articulate with the quadrate condyles and are separated by a shallow, anteromedially-oriented ridge. The glenoid region of *Allosaurus* possesses a much sharper ridge than that of *Acrocanthosaurus* [21]. The articular of *Sinraptor* also shares this pronounced glenoid ridge, although the ridge in *Mapusaurus* is greatly reduced. No antarticulars are ossified in *Acrocanthosaurus*; the only allosauroid known to possess this proposed neomorph is *Allosaurus* [16], [69].

Along the medial surface of the articular, a bowl-shaped projection envelops the foramen posterior chorda tympani (Figure 32B). Immediately posterior to this projection, the semi-circular retroarticular process of the articular is expanded posterodorsally. Excluding this process, the posterior margin of the articular is steeply inclined anterodorsally. Anterior to the retroarticular process, a tall, rounded spine forms the posterior region of the glenoid fossa [21]. This spine is taller and more strongly developed in *Acrocanthosaurus* than in any other allosauroid from which an articular has been described, including *Sinraptor*, *Monolophosaurus*, *Yangchuanosaurus*, and *Allosaurus*. However, the presence of a similarly pronounced spine in *Mapusaurus* suggests that this feature may be distributed more broadly within Carcharodontosauridae. This spine is reduced in non-allosauroid theropods, including *Coelophysis*, *Cryolophosaurus*, and *Tyrannosaurus* [13], [27], [82], [89].

### Phylogenetic analysis of Allosauroidea

**Figure 33:**
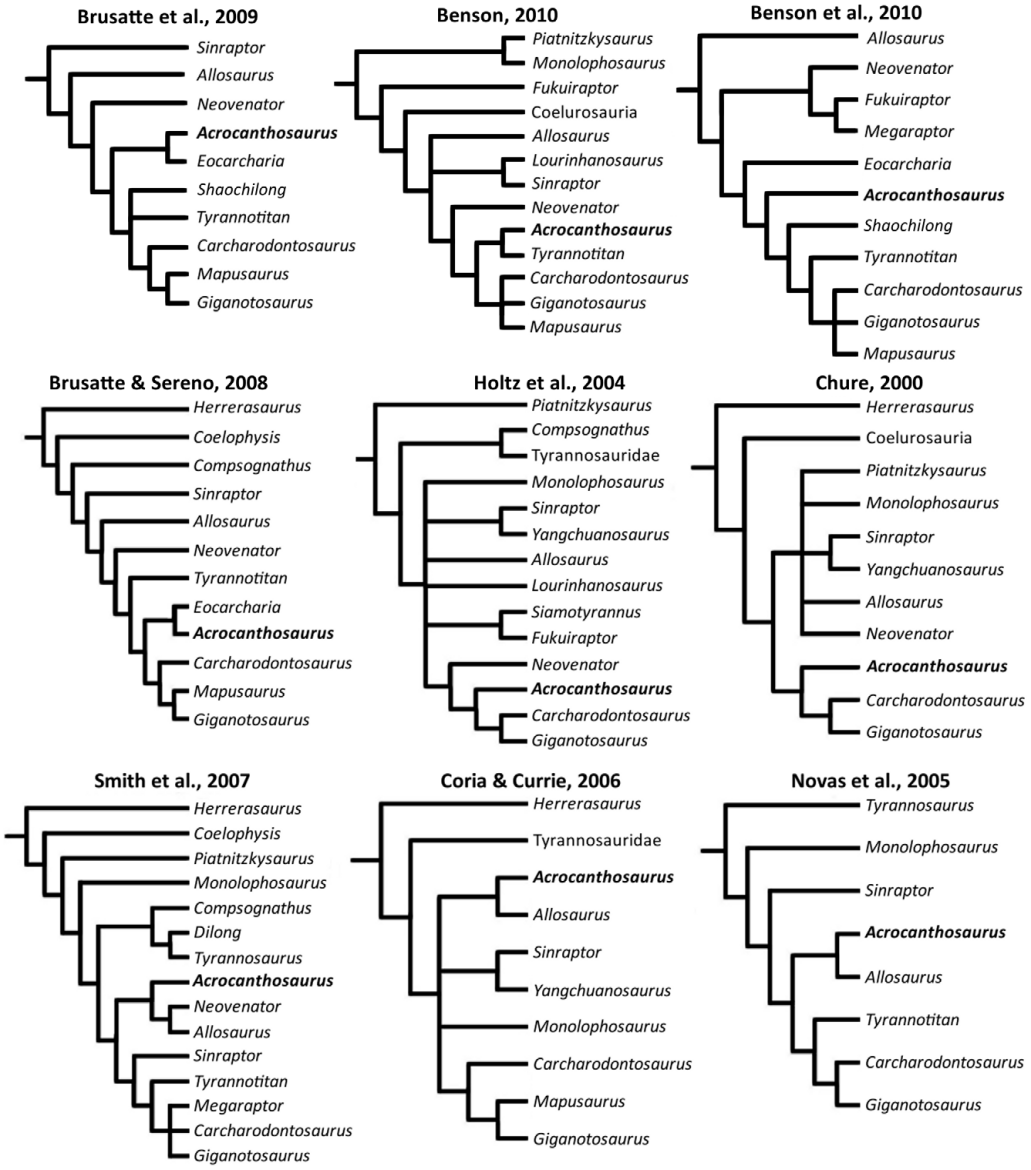
Nine phylogenies recovered by systematic analyses of Allosauroidea and outgroup taxa. Some trees have been pruned to show only those taxa incorporated into the present study. Analyses along the top two rows recover *Acrocanthosaurus atokensis* as a member of Carcharodontosauridae, whereas the bottom row represents those that place *Acrocanthosaurus atokensis* as close relative and/or sister taxon to *Allosaurus*.

The phylogenetic placement of Allosauroidea within Theropoda is well-established (Figure 1). Most researchers recover Coelurosauria as the sister taxon to Allosauroidea, with Megalosauroidea ( = Spinosauroidea) as the nearest outgroup to this relationship [10], [12], [26], [105]–[107] (although see [9]). Despite the phylogenetically-consistent position of Allosauroidea in relation to other large theropod groups, relationships within Allosauroidea have been contentious (Figure 33). Numerous systematic studies during the past fifteen years have addressed the group [1], [9], [10], [12], [13], [19]–[22], [25], [26], [36], [37], [39]–[42], [53]. However, when eleven of these analyses were trimmed to six shared allosauroid taxa (*i.e.*, *Sinraptor*, *Allosaurus*, *Neovenator*, *Acrocanthosaurus*, *Carcharodontosaurus*, and *Giganotosaurus*) and combined in a strict consensus tree, the result was a completely unresolved polytomy [22].

The phylogenetic position of *Acrocanthosaurus atokensis* within Allosauroidea has been a source of conflict. Several phylogenies recover *Acrocanthosaurus* as a sister taxon or close relative to *Allosaurus* [1], [13], [17], [36], [39], [53], [56]. However, recent work on the phylogenetic resolution of Allosauroidea provides robust support for the placement of *Acrocanthosaurus* within the subclade Carcharodontosauridae [10], [22], [25], [42], a hypothesis that corroborates several previous analyses [10], [12], [19]–[21], [59], [49]. Morphological description and scoring of previously undescribed cranial characters from *Acrocanthosaurus* specimen NCSM 14345 has brought new data to bear upon the systematic position of the taxon. These new characters provide increasing support for the resolution of allosauroid interrelationships, including a single most parsimonious placement of *Acrocanthosaurus*. From this analysis, hypotheses concerning the evolution of the allosauroid skull and body size are evaluated, a revised cranial diagnosis of the species *Acrocanthosaurus atokensis* is proposed, and consistencies with the stratigraphic record and biogeographical distribution of Allosauroidea are considered.

The primary phylogenetic analysis scores an ingroup of 12 taxa comprising the most consistently recovered members of Allosauroidea. These taxa are listed in boldface in Table S1 with their OTUs, referred species, percentages of missing character data, and sources for character scores. The majority of scorings taken from the literature were evaluated through personal observation of specimens and images provided by other researchers (see Acknowledgments), although published illustrations were consulted when necessary. Species exemplars are used for *Sinraptor*, *Yangchuanosaurus*, *Allosaurus*, and *Carcharodontosaurus*, and are scored from the species that best comprise the exemplars. Conversely, *Neovenator*, *Tyrannotitan*, *Eocarcharia*, *Acrocanthosaurus*, *Giganotosaurus*, and *Mapusaurus* are all monotypic. Only three included terminals (*i.e.*, *Yangchuanosaurus*, *Eocarcharia*, and *Tyrannotitan*) present greater than 80% missing data, and the most complete taxa in the dataset, *Allosaurus* and *Acrocanthosaurus*, have 0% and 6% missing data, respectively. The monotypic taxon *Monolophosaurus* is also evaluated. Several phylogenetic analyses recover *Monolophosaurus* in an unresolved polytomy, or as the most basal member of Allosauroidea (Figure 33) [12], [19], [26], [36], [39]. However, recent analyses support its placement outside of Allosauroidea entirely [10], [13], [42], [48], [50]. The recently described allosauroid taxa *Aerosteon* and *Concavenator* were not included in the phylogenetic analysis, although more comprehensive theropod analyses have recently examined the systematic position of these two taxa and support their placement within Allosauroidea [25], [42].

Six non-allosauroid supraspecific terminals and species exemplars are included as outgroups to determine character-state polarity within Allosauroidea. Three of these terminals, *Herrerasaurus*, Coelophysoidea Holtz 1994 [17] and *Piatnitzkysaurus* Bonaparte 1979 [108], are chosen as good candidates for being closely related outgroup taxa to the clade Allosauroidea + Coelurosauria [9], [22], [109] with *Piatnitzkysaurus* as a species exemplar sampling Megalosauroidea. *Herrerasaurus* is selected to be the only constrained outgroup taxon. The remaining three terminals, *Tyrannosaurus*, *Dilong*, and Compsognathidae Cope 1871 [110], comprise basal members of Coelurosauria, the established sister taxon to Allosauroidea [12], [14], [17]. Although the use of the supraspecific taxa Coelophysoidea and Compsognathidae is not ideal [111], evaluating every distinct terminal within those clades is outside the scope of the present analysis.

The data matrix totals 177 characters (Appendix S1), comprising 103 cranial characters (58.2%), 31 axial characters (17.5%), and 43 appendicular characters (24.3%). Thirteen multi-state characters (7, 15, 23, 28, 48, 58, 73, 91, 99, 157, 158, 172, and 174) are ordered upon determination of a likely morphocline following Slowenski [112] and are indicated as such in Appendix S1. Comparisons between the character matrix in the present analysis and those from other recent phylogenetic analyses of Allosauroidea are provided in Table 4.

**Table 4:** Comparison of the number of total characters, number of characters pertaining to cranial data, and number of phylogenetically informative characters for Allosauroidea among present and prior analyses illustrated in Figure 33.

	Total Characters	Cranial Characters (% of Total Dataset)	Characters Informative for Allosauroidea
**Present Analysis**	**177**	**103 (58.2%)**	**136**
Brusatte and Sereno [22]	99	59 (59.6%)	99
Holtz *et al*. [12]	638	272 (42.6%)	185
Chure [19]	112	50 (44.6%)	57
Smith *et al*. [13]	347	141 (40.6%)	108
Coria and Currie [36]	110	42 (38.2%)	66
Novas *et al*. [39]	108	?	?
Brusatte *et al*. [37]	106	67 (63.2%)	106
Benson [10]	213	100 (46.9%)	101
Benson *et al*. [42]	233	102 (43.8%)	113

Supplemental information for Novas *et al*. [39], containing the data matrix used in the analysis, was not accessible from the online source provided by the publication.

**Figure 34:**
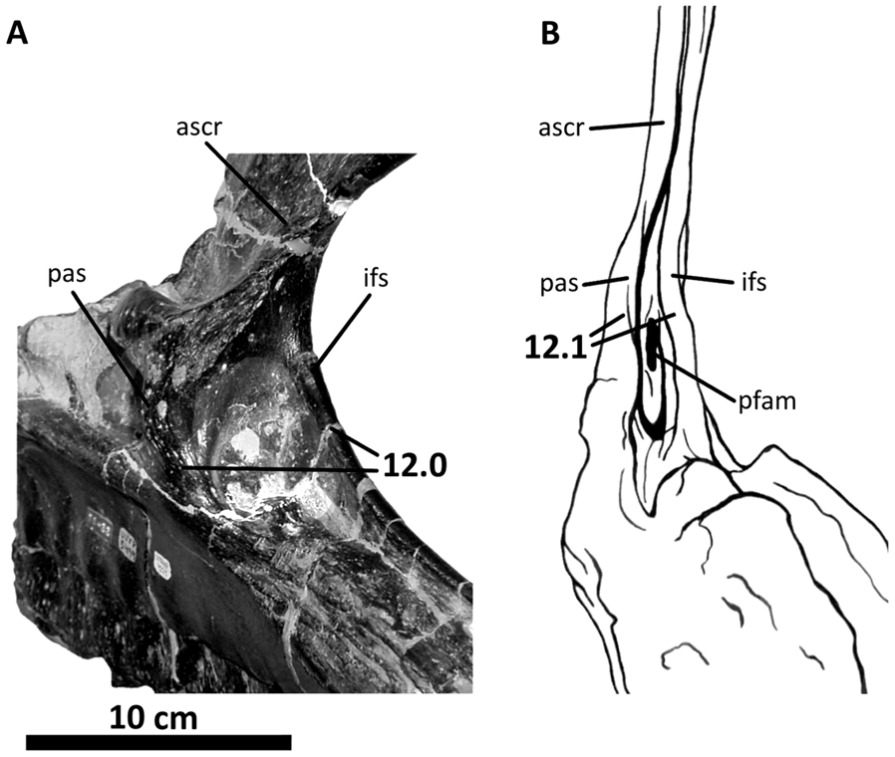
Illustration of character 12 (Appendix S1). Right maxilla of (A) *Allosaurus fragilis* (UUVP 5499) in posteromedial view and (B) *Acrocanthosaurus atokensis* (NCSM 14345) in posterior view. **ascr**, ascending ramus of the maxilla; **pfam**, posterior maxillary fenestra; **ifs**, interfenestral strut; **pas**, postantral strut.

Twenty-four new morphological characters are identified from comparative study and description of the skull of NCSM 14345 (Figures 34–50). These new characters comprise 13.5% of the analysis, and many are identified from elements that are often inaccessible, highly fragmentary, or not preserved in specimens referred to the taxa included in this analysis (*e.g.*, ectopterygoid, pterygoid, epipterygoid, prearticular). The remaining 86.5% of the analysis relies upon 142 characters taken from seventeen previously published theropod phylogenies [1], [9], [10], [12], [13], [20]–[22], [26], [40], [41], [53], [56], [105], [109], [113], [114]. The majority of previously published characters are taken from analyses of basal tetanurans by Holtz *et al*. [12] and Smith *et al*. [13], and an analysis of allosauroid interrelationships by Brusatte and Sereno [22] (note that many of these characters also overlap with those in Benson [10] and Benson *et al*. [42]). These highlighted datasets provide 22, 46, and 61 characters, respectively, to the present analysis, a total of 72.8%. Overlapping characters and those non-informative to Allosauroidea have been removed, although several characters from Holtz *et al*. [12] are included to help resolve Coelurosaurian relationships. The two analyses by Smith *et al*. [13] and Brusatte and Sereno [22] are chosen to provide the majority of the characters taken from the literature because they are relatively recent, encompass most characters previously proposed by the seventeen analyses mentioned above, and yield allosauroid phylogenies that differ with respect to the placement of *Acrocanthosaurus*. For example, Brusatte and Sereno recover *Acrocanthosaurus* as the sister taxon to *Eocarcharia* near the base of Carcharodontosauridae [22], whereas Smith *et al*. recover *Acrocanthosaurus* as more closely related to *Allosaurus* [13].

**Figure 35 pone-0017932-g035:**
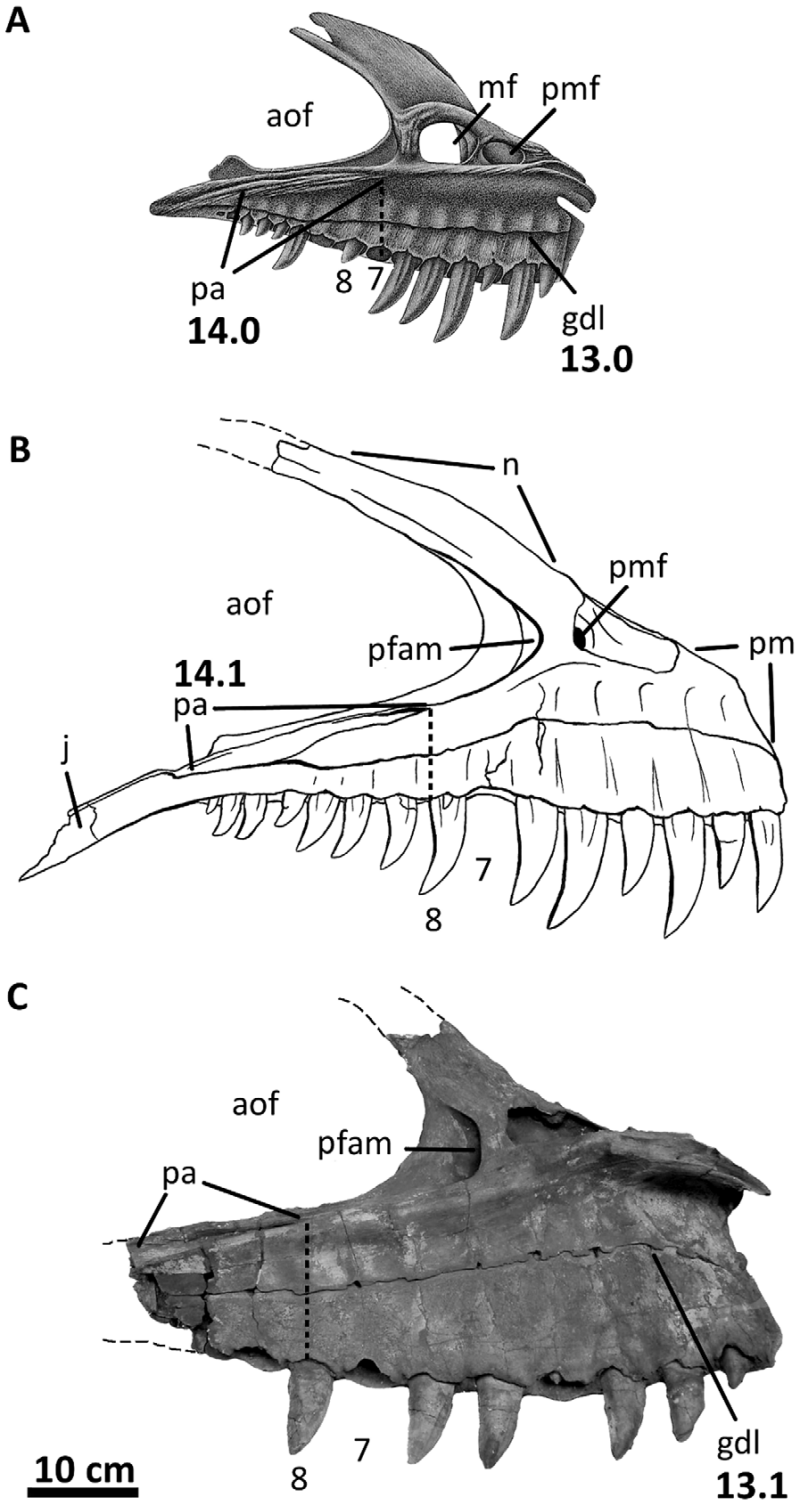
Illustration of characters 13 and 14 (Appendix S1). Left maxillae of (A) *Allosaurus fragilis* (figure modified from [69]), (B) *Acrocanthosaurus atokensis* (NCSM 14345) and (C) *Carcharodontosaurus saharicus* (SGM-Din 1) in medial view. Thick vertical dashed lines represent the anterior extent of palatal contact; thin dashed lines represent material not in the figure. **aof**, antorbital fenestra; **gdl**, groove for dental lamina of the maxilla; **j**, jugal contact; **mf**, maxillary fenestra; **n**, nasal contact; **pa**, palatine contact; **pfam**, posterior maxillary fenestra; **pmf**, promaxillary fenestra.

**Figure 36 pone-0017932-g036:**
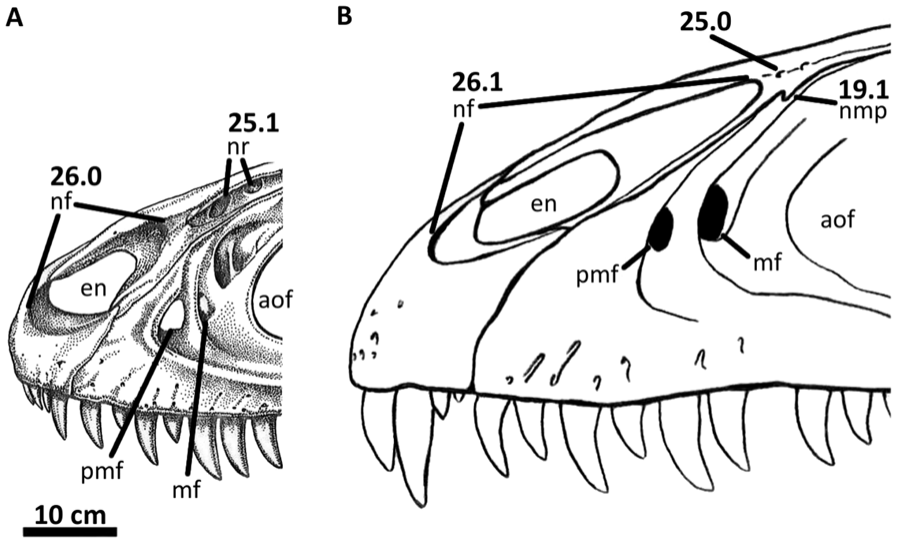
Illustration of characters 19, 25, and 26 (Appendix S1). Left rostra of (A) *Sinraptor dongi* (figure modified from [16]) and (B) *Acrocanthosaurus atokensis* (NCSM 14345). **aof**, antorbital fenestra; **en**, external naris; **mf**, maxillary fenestra; **nf**, narial fossa; **nmp**, naso-maxillary process; **nr**, nasal recesses; **pmf**, promaxillary fenestra.

**Figure 37 pone-0017932-g037:**
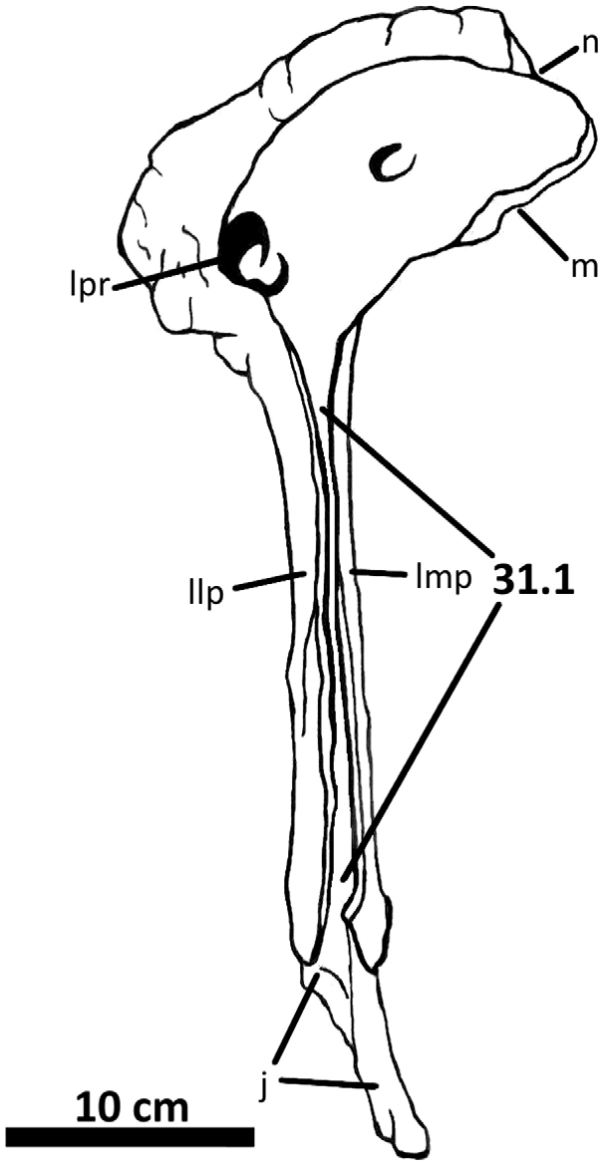
Illustration of character 31 (Appendix S1). Right lacrimal of *Acrocanthosaurus atokensis* (NCSM 14345) in anterolateral view. **j**, jugal contact; **llp**, lateral lacrimal plate; **lmp**, lacrimal medial plate; **lpr**, lacrimal pneumatic recess; **m**, maxillary contact; **n**, nasal contact.

**Figure 38 pone-0017932-g038:**
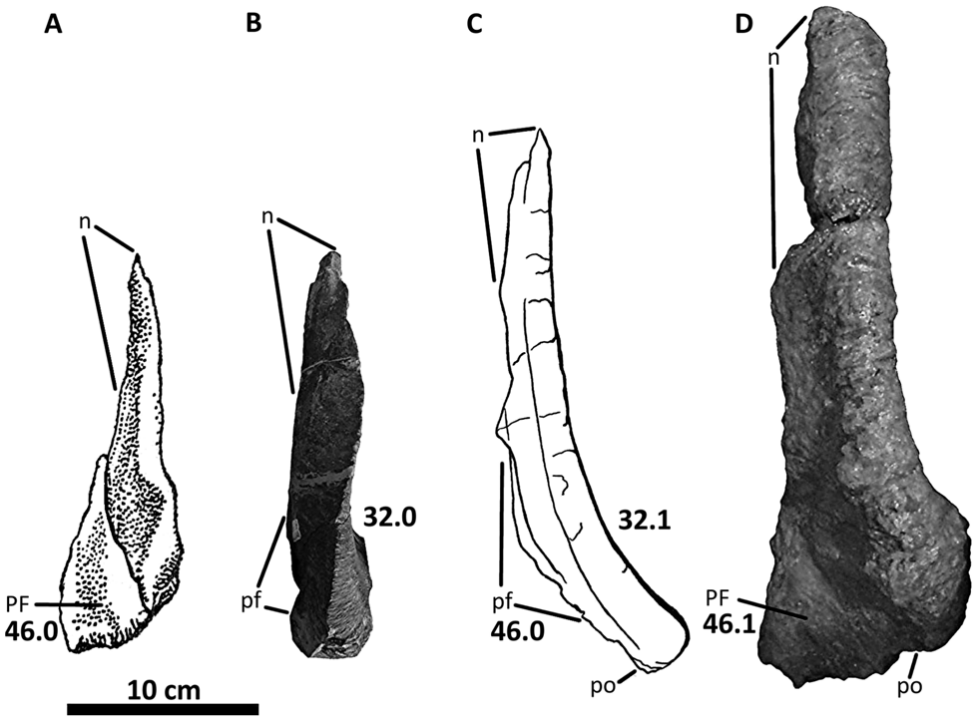
Illustration of characters 32 and 46 (Appendix S1). Right lacrimals of (A) *Sinraptor dongi* (figure modified from [16]), (B) *Allosaurus fragilis* (UUVP 5198), (C) *Acrocanthosaurus atokensis* (NCSM 14345), and (D) *Giganotosaurus carolinii* (MUCPv-CH-1) in dorsal view. **n**, nasal contact; **PF**, prefrontal; **pf**, prefrontal contact; **po**, postorbital contact.

**Figure 39 pone-0017932-g039:**
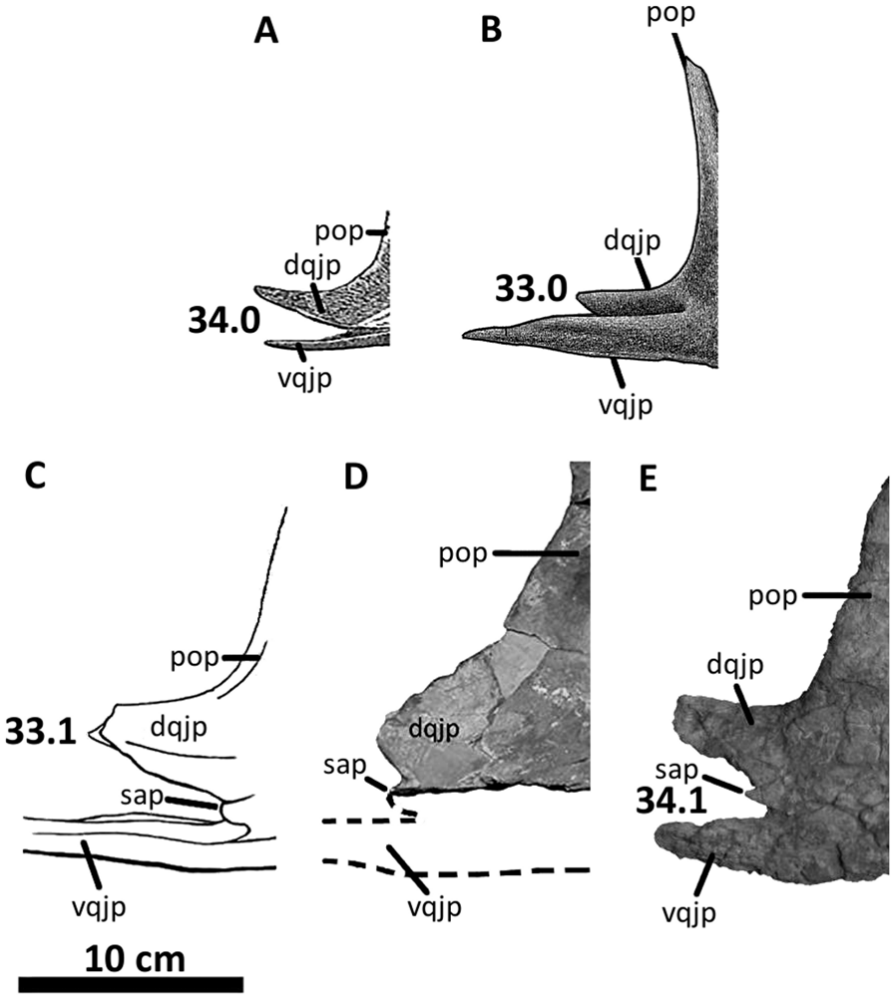
Illustration of characters 33 and 34 (Appendix S1). Right jugals of (A) *Sinraptor dongi* (figure modified from [16]), (B) *Allosaurus fragilis* (figure modified from [69]), (C) *Acrocanthosaurus atokensis* (NCSM 14345), (D) *Carcharodontosaurus saharicus* (SGM-Din 1), and (E) *Mapusaurus roseae* (MCF-PVPH-108.167) in lateral view. **dqjp**, dorsal quadratojugal prong; **pop**, postorbital process of jugal; **sap**, small accessory prong; **vqjp**, ventral quadratojugal prong.

**Figure 40 pone-0017932-g040:**
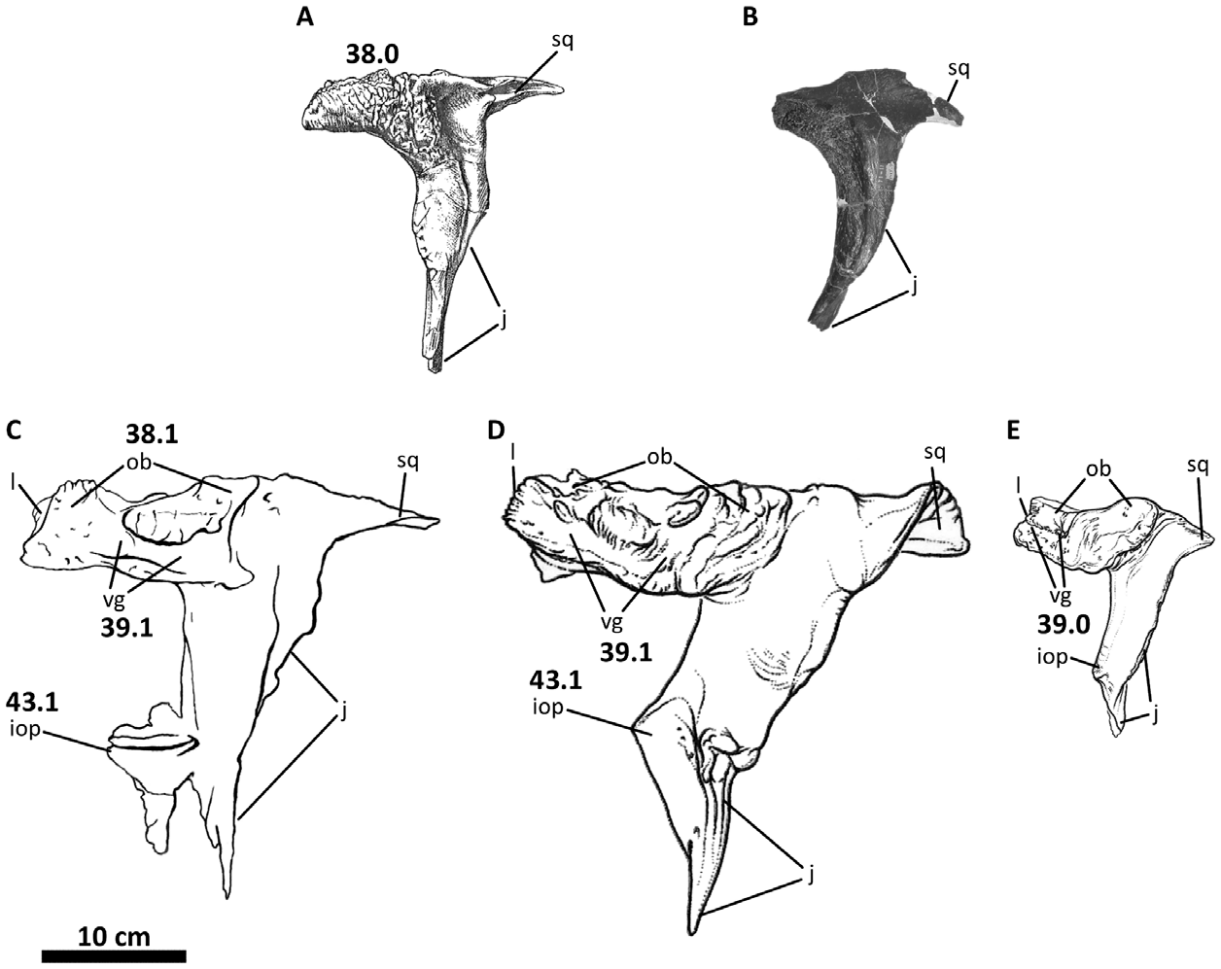
Illustration of characters 38, 39, and 43 (Appendix S1). Left postorbitals of (A) *Sinraptor dongi* (modified from [16]), (B) *Allosaurus fragilis* (UUVP 5958); (C) *Acrocanthosaurus atokensis* (NCSM 14345), (D) *Carcharodontosaurus saharicus* (figure modified from [49]), and (E) *Eocarcharia dinops* (figure modified from [49]) in lateral view. **iop**, intra-orbital process; **j**, jugal contact; **l**, lacrimal contact; **ob**, orbital boss; **sq**, squamosal contact; **vg**, vascular groove.

**Figure 41 pone-0017932-g041:**
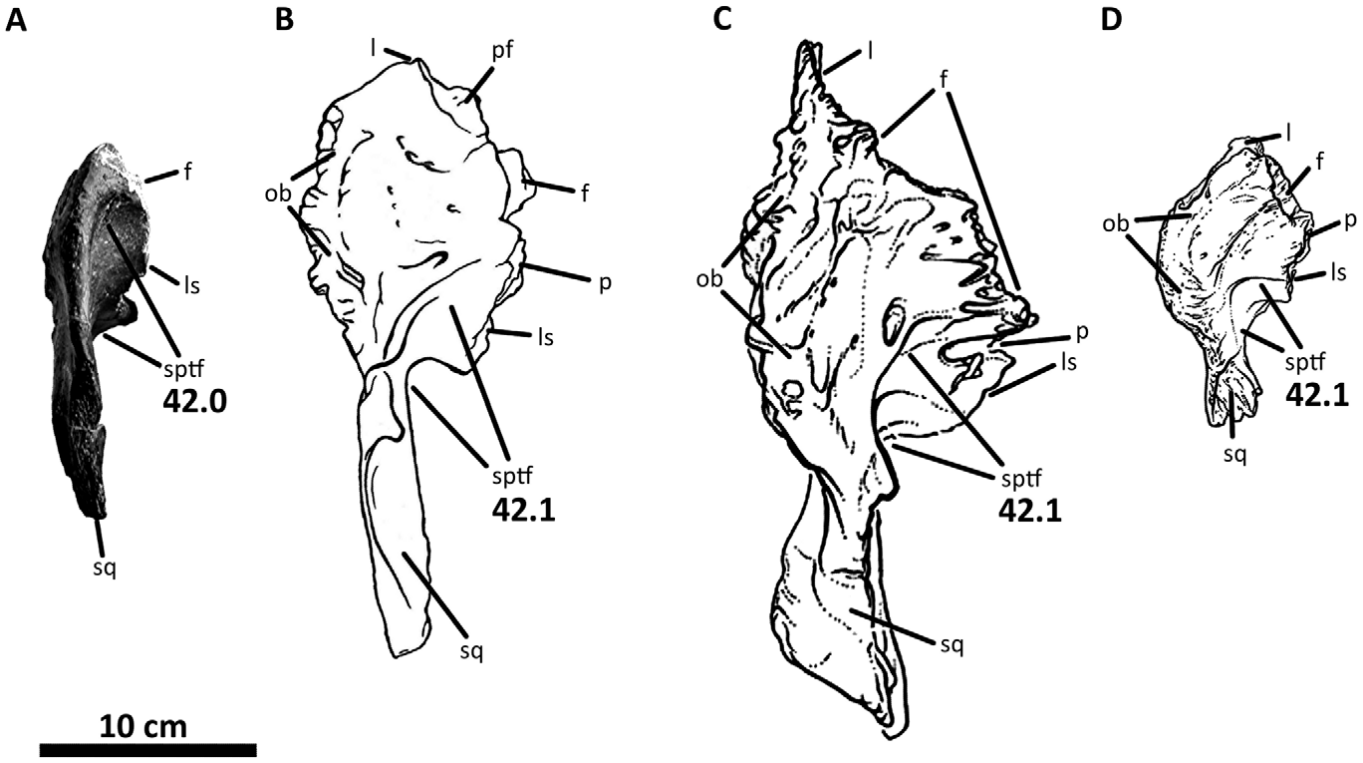
Illustration of character 42 (Appendix S1). Left postorbitals of (A) *Allosaurus fragilis* (UUVP 5160), (B) *Acrocanthosaurus atokensis* (NCSM 14345), (C) *Carcharodontosaurus saharicus* (figure modified from [49]), and (D) *Eocarcharia dinops* (figure modified from [49]) in dorsal view. **f**, frontal contact; **l**, lacrimal contact; **ls**, laterosphenoid contact; **ob**, orbital boss; **p**, parietal contact; **pf**, prefrontal contact; **sptf**, supratemporal fossa; **sq**, squamosal contact.

**Figure 42 pone-0017932-g042:**
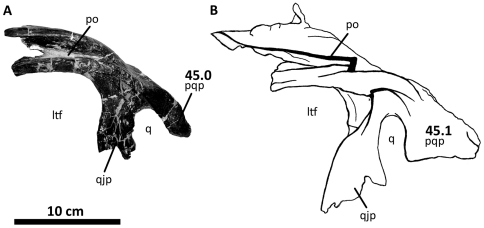
Illustration of character 45 (Appendix S1). Left squamosals of (A) *Allosaurus fragilis* (AMNH 14554) and (B) *Acrocanthosaurus atokensis* (NCSM 14345) in lateral view. **ltf**, lateral temporal fenestra; **po**, postorbital contact; **pqp**, post-quadratic process ( = postcotyloid); **q**, quadrate contact; **qjp**, quadratojugal process.

**Figure 43 pone-0017932-g043:**
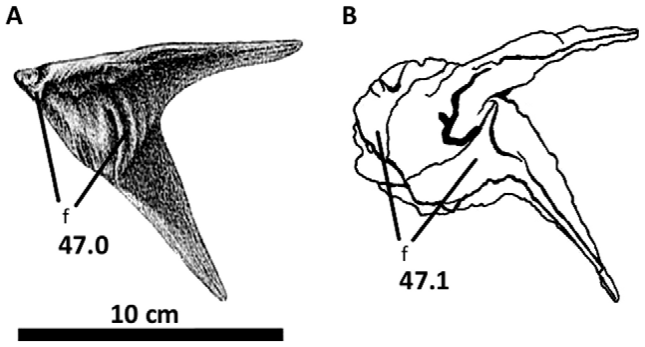
Illustration of character 47 (Appendix S1). Left prefrontals of (A) *Allosaurus fragilis* (image modified from [69]) and (B) *Acrocanthosaurus atokensis* (NCSM 14345) in medial view. **f**, frontal contact.

**Figure 44 pone-0017932-g044:**
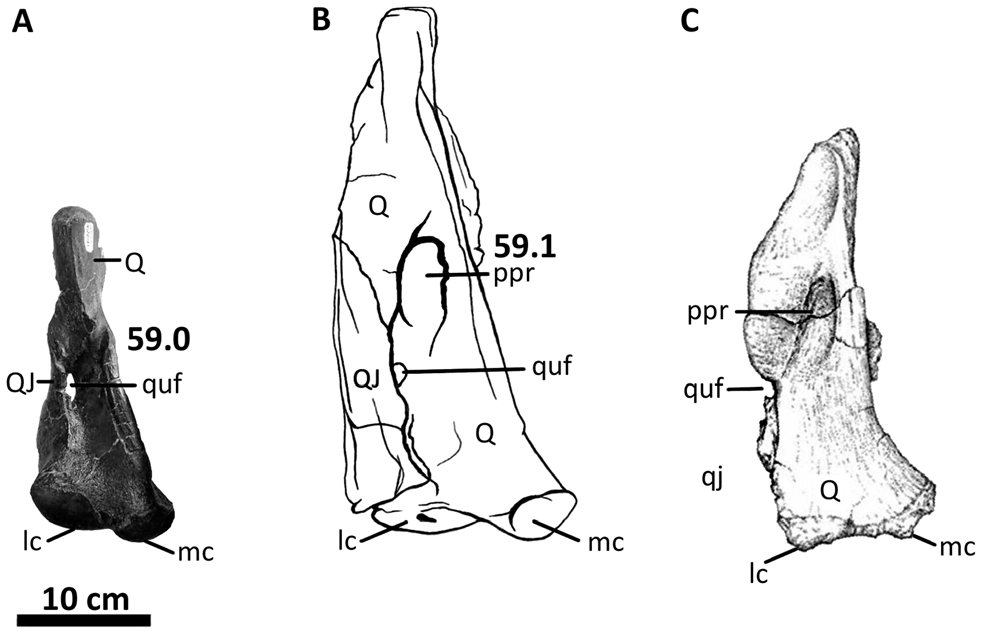
Illustration of character 59 (Appendix S1). Left quadrates of (A) *Allosaurus fragilis* (UUVP 3082), (B) *Acrocanthosaurus atokensis* (NCSM 14345), and (C) *Mapusaurus roseae* (image modified from [36]) in posterior view. **lc**, lateral condyle; **mc**, medial condyle; **ppr**, posterior pneumatic recess; **Q**, quadrate; **QJ**, quadratojugal; **qj**, space occupied by quadratojugal; **quf**, quadrate foramen.

**Figure 45 pone-0017932-g045:**
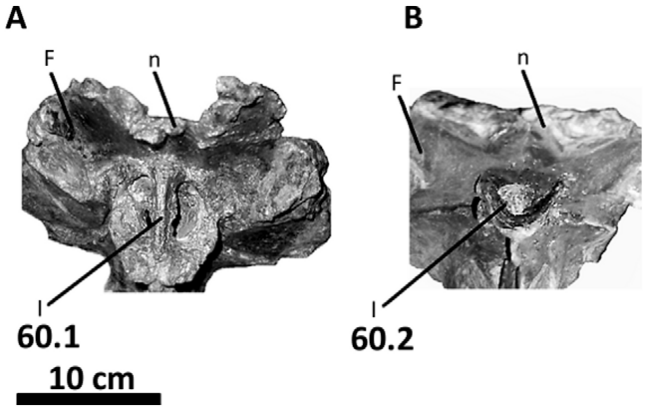
Illustration of character 60 (Appendix S1). Braincase of (A) *Acrocanthosaurus atokensis* (NCSM 14345) and (B) Allosaurus *fragilis* (UUVP 3082) in anterior view. **F**, frontal; **I**, exit for olfactory nerve; **n**, nasal contact.

**Figure 46 pone-0017932-g046:**
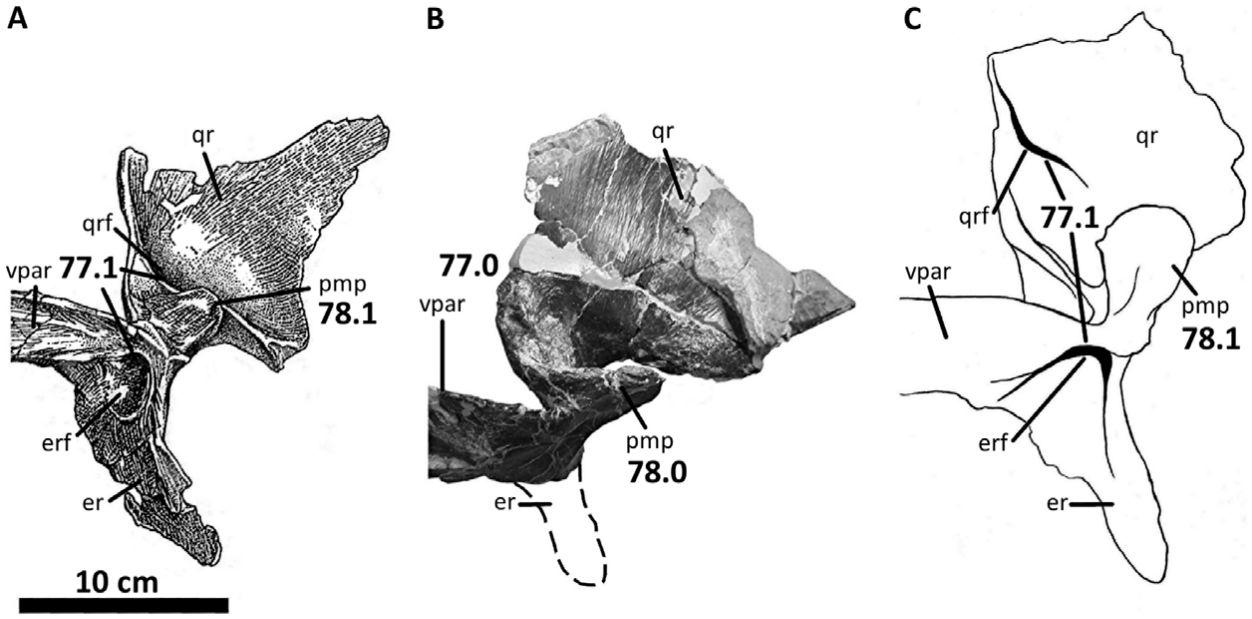
Illustration of characters 77 and 78 (Appendix S1). Right pterygoids of (A) *Sinraptor dongi* (image modified from [16]), (B) *Allosaurus fragilis* (UUVP 5748), and (C) *Acrocanthosaurus atokensis* (NCSM 14345) in medial view. Dashed lines represent missing material. **er**, ectopterygoid ramus; **erf**, ectopterygoid ramus fossa; **pmp**, pterygoid medial process; **qr**, quadrate ramus; **qrf**, quadrate ramus fossa; **vpar**, vomeropalatine ramus.

**Figure 47 pone-0017932-g047:**
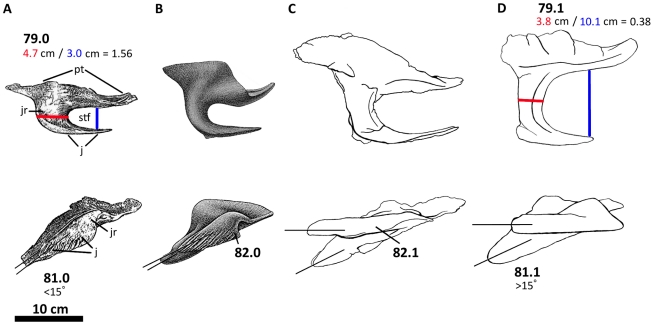
Illustration of characters 79, 81, and 82 (Appendix S1). Left ectopterygoids of (A) *Sinraptor dongi* (image modified from [16]), (B) *Allosaurus fragilis* (image modified from [69]), (C) *Acrocanthosaurus atokensis* (NCSM 14345), and (D) *Giganotosaurus carolinii* (MUCPv-CH-1) in dorsal (top row) and lateral (bottom row) views. Characters 81 and 82 appear to be dependent upon first examination, as both *Acrocanthosaurus* and *Giganotosaurus* share an ectopterygoid with a narrow jugal ramus (82∶1) that is rotated dorsally (81∶1), in contrast to the more robust jugal ramus (82∶0) that lies parallel to the main body of the ectopterygoid (81∶0) in *Allosaurus* and *Sinraptor*. However, the presence of a dorsally rotated jugal ramus (81∶1) in *Carnotaurus* (a basal theropod consistently recovered outside of Allosauroidea [81]) coinciding with a wide jugal ramus and narrow subtemporal fenestra (82∶0), suggests that the states are independent. **j**, jugal contact; **jr**, jugal ramus; **pt**, pterygoid contact; **stf**, subtemporal fenestra.

**Figure 48 pone-0017932-g048:**
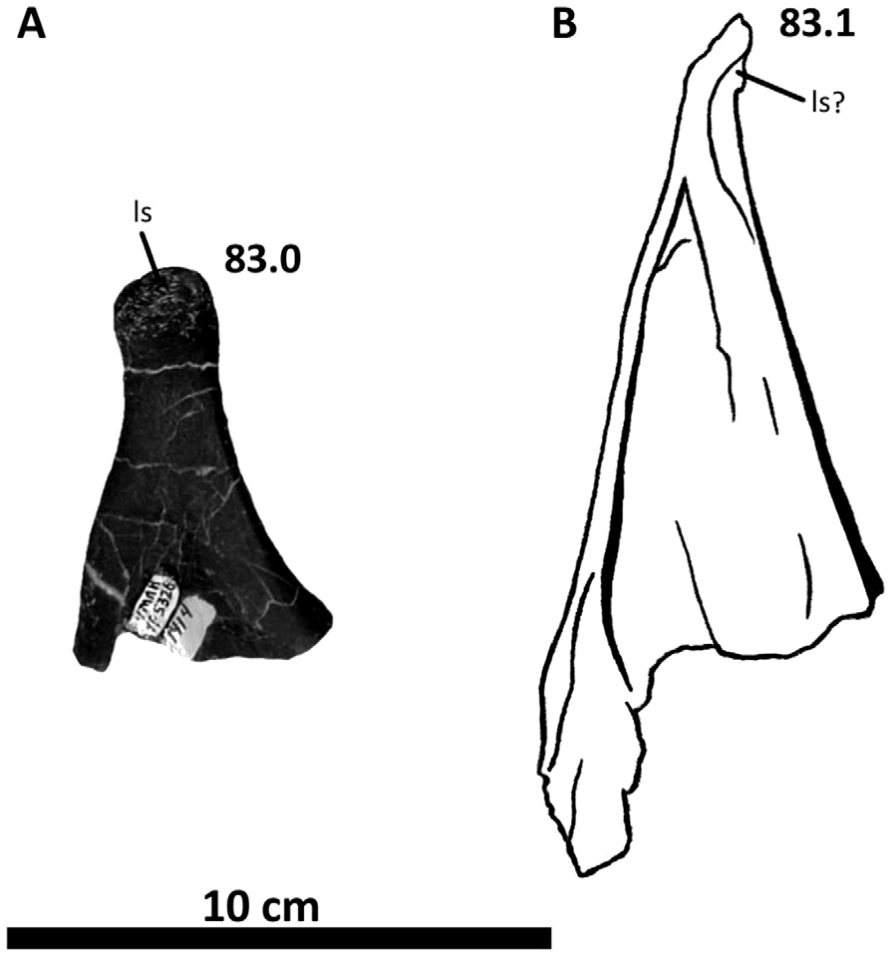
Illustration of character 83 (Appendix S1). Left epipterygoids of (A) *Allosaurus fragilis* (UUVP 1414) and (B) *Acrocanthosaurus atokensis* (NCSM 14345) in medial view. Both elements shown are complete. **ls**, laterosphenoid contact.

**Figure 49 pone-0017932-g049:**
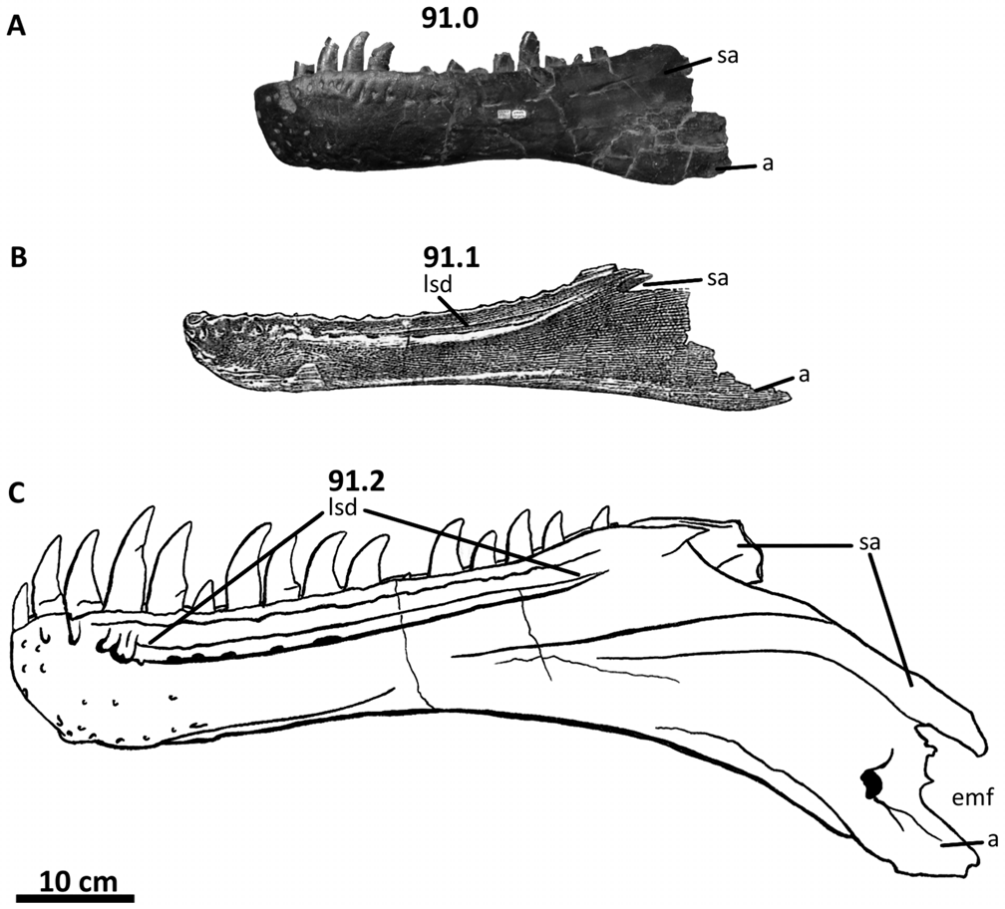
Illustration of character 91 (Appendix S1). Left dentaries of (A) *Allosaurus fragilis* (UUVP 871), (B) *Sinraptor dongi* (image modified from [16]), and (C) *Acrocanthosaurus atokensis* (NCSM 14345) in lateral view. **a**, angular contact; **emf**, external mandibular fenestra; **lsd**, lateral sulcus of the dentary; **sa**, surangular contact.

**Figure 50:**
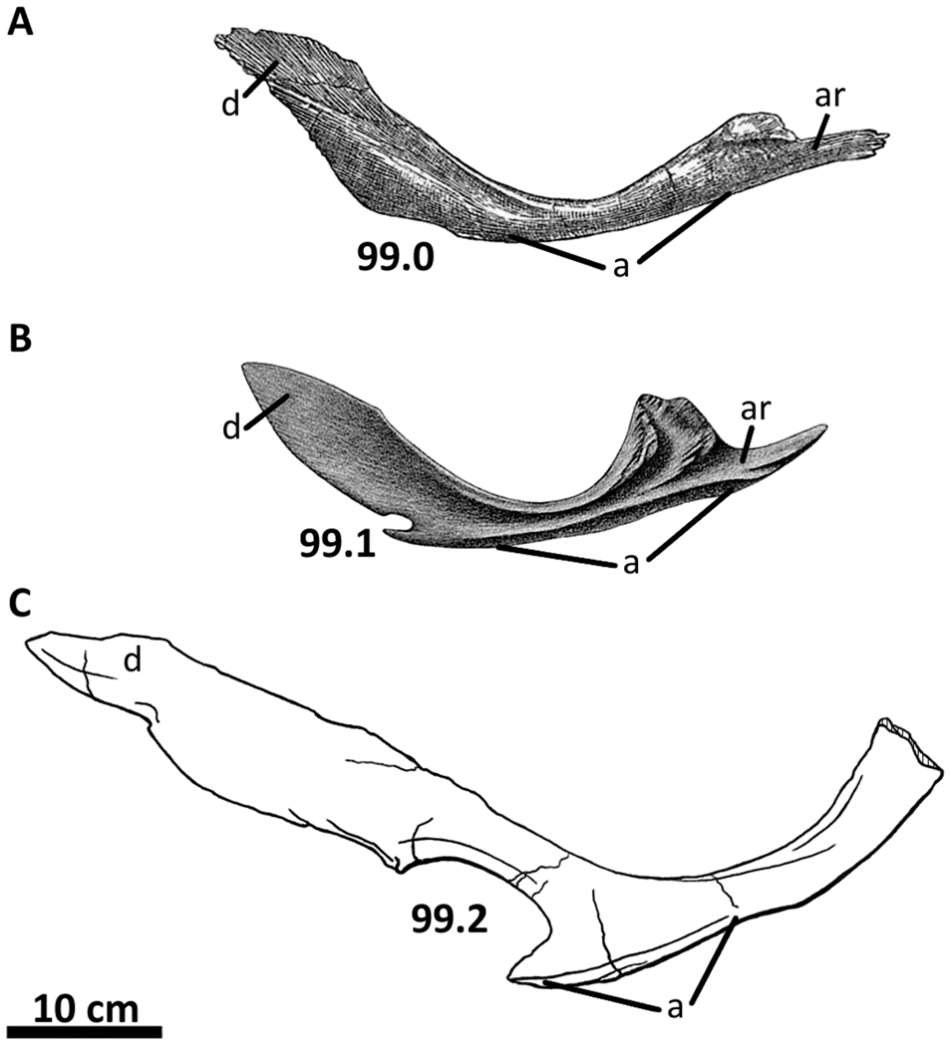
Illustration of character 99 (Appendix S1). Left prearticulars of (A) *Sinraptor dongi* (image modified from [16]), (B) *Allosaurus fragilis* (image modified from [69]), and (C) *Acrocanthosaurus atokensis* (NCSM 14345) in internal views. Hatched lines indicate broken surfaces. **a**, angular contact; **ar**, articular contact; **d**, dentary contact.

The data matrix for the present analysis (Appendix S2) was edited in MESQUITE v.2.0 [115], and analyzed in TNT v.1.1 [116] using the implicit enumeration option (maximum trees =  10,000) and PAUP*v.4.0b10 [117] using the branch-and-bound search option (maximum trees  =  1,000). In both TNT and PAUP*, branches were collapsed to soft polytomies if their minimum length equaled zero. The robustness of the resulting most parsimonious tree (MPT) was evaluated using bootstrap (from 1,000 replicates, same settings as in the primary analysis) [118] and Bremer support values [119]. Character state optimizations were assessed in MacClade and MESQUITE.

**Figure 51:**
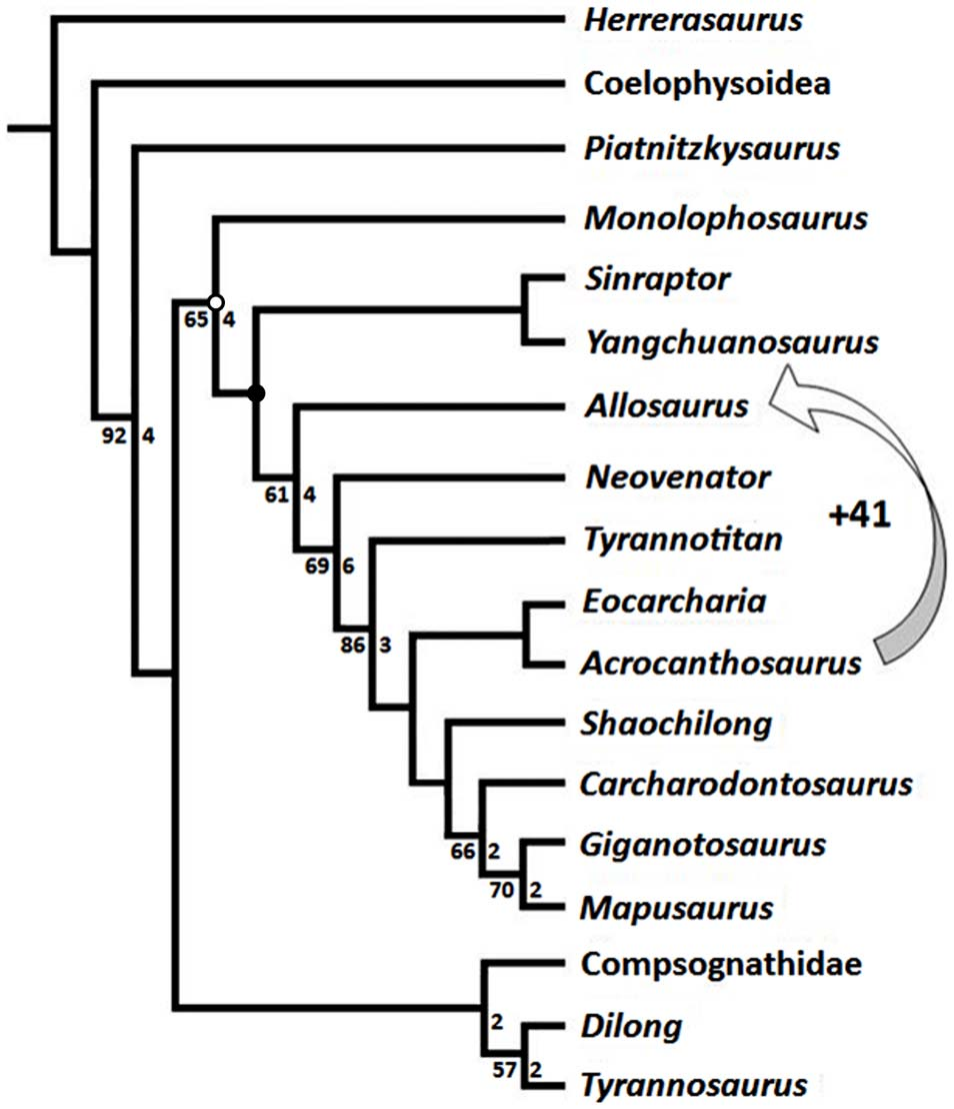
Single most parsimonious tree resulting from the phylogenetic analysis. Primary analysis included 177 characters evaluated for 18 taxa (Length =  325 steps; CI  =  0.60; RI =  0.66; RCI  =  0.39). Bootstrap values >50% are to the left of the nodes; Bremer support values >1 are to the right. The open circle represents Carnosauria; the closed circle represents Allosauroidea. The arrow shows the number of steps needed to remove *Acrocanthosaurus* from its current position within Carcharodontosauridae and place it as the sister taxon to *Allosaurus*, a relationship proposed by previous authors (Figure 33).

Analysis of the 18 primary taxa (*i.e.*, excluding *Fukuiraptor*, *Lourinhanosaurus*, *Siamotyrannus*, and *Australovenator*) produced a single most parsimonious tree in both PAUP* and TNT (Figure 51). The resultant tree is 325 steps in length with a consistency index (CI) of 0.60, a retention index (RI) of 0.66, and a rescaled consistency index (RCI) of 0.39. Branches recovered in greater than 50% bootstrap replicates are reported, as are Bremer support values. Values for these metrics are relatively low, suggested as typical for analyses involving taxa with substantial amounts of missing data [118]–[119].

Both Carnosauria and Allosauroidea are found to be monophyletic, with a relatively high Bremer value of 4 recovered for Carnosauria. Within Allosauroidea, *Acrocanthosaurus* is not recovered as the sister taxon to *Allosaurus*, but is instead nested relatively deeply within Carcharodontosauridae. An additional 41 steps would be added to the tree length to place *Acrocanthosaurus* as the sister taxon to *Allosaurus* (Figure 51). *Acrocanthosaurus* is found to be the sister taxon to *Eocarcharia*. This sister grouping is the nearest outgroup to *Shaochilong* + Carcharodontosaurinae (a carcharodontosaurid subclade comprised of *Carcharodontosaurus*, *Mapusaurus*, and *Giganotosaurus*).

Exclusion of *Monolophosaurus* from the primary phylogenetic analysis does not substantially alter the structure of the recovered tree, except that its removal collapses Sinraptoridae (*Sinraptor* + *Yangchuanosaurus*) at the base of Allosauroidea. Given the contentious placement of *Monolophosaurus* as an allosauroid [9], [10], [12], [13], [22], [36], [42], it is not surprising that low bootstrap (<50%) and Bremer support is shown for Allosauroidea; including *Monolophosaurus* within Allosauroidea adds only one step to the overall tree length. The phylogenetic placement of *Monolophosaurus* has varied widely (Figure 33). *Monolophosaurus* has been recovered as part of Allosauroidea [12], [19], [22], [36] and as a carnosaur placed outside of Allosauroidea [9], [39], and has been suggested to be a basal tetanuran [13]. Given that more recent comprehensive analyses of basal theropods strongly support *Monolophosaurus* as a megalosauroid [10], its placement within Carnosauria by the present analysis (as well as the high Bremer support for Carnosauria) must be viewed as tentative.

Several unambiguously optimized synapomorphies support the monophyly of Carnosauria in this analysis. These synapomorphies have been recognized by previous analyses [9], [12], [13], [22] and include: (character 23∶2) lateral surface of nasal participating in antorbital fossa; (55∶1) a pronounced, posteriorly-placed dorsal projection of parietal; (63∶1) transverse distance across basal tubera less than width of occipital condyle; (85∶1) palatines meet medially; (86∶1) tetra-radiate palatine; (98∶1) articular with a pendant medial process; and (106∶1) ventral margin of the axial intercentrum angled strongly dorsally (see Appendix S1 for the original authorship of these and subsequently discussed characters). Allosauroidea is supported by four previously recognized, unambiguously optimized synapomorphies: (character 5∶1) subnarial process of the premaxilla strongly reduced in width but still contacts nasals; (72∶1) paroccipital processes of the braincase deflected below level of occipital condyle; (119∶1) dorsal vertebrae with hourglass-shaped centrum and dorsoventral thickness less than 60% height of cranial face; and (155∶1) obturator foramen of pubis open ventrally.

**Figure 52:**
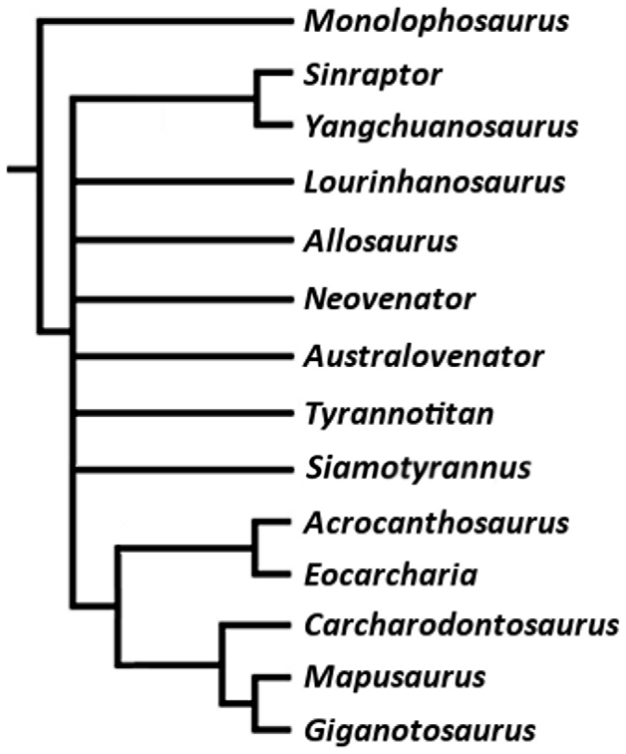
Phylogeny of Allosauroidea upon the inclusion of proposed taxa known from highly fragmentary specimens. Included taxa are *Siamotyrannus*, *Lourinhanosaurus*, and *Australovenator*. A full phylogenetic analysis of 20 terminals recovered 11 MPT's (TL  =  338 steps), the strict consensus of which is shown. This phylogeny has been cropped to show only allosauroid relationships, as the outgroup taxa were unaffected.

A secondary phylogenetic analysis includes “problematic” taxa with less frequently recovered allosauroid affinities. All three taxa in the secondary analysis (*i.e.*, *Siamotyrannus*, *Lourinhanosaurus*, and *Australovenator*) are monotypic. These taxa possess a substantial amount of missing data (>85%) and are excluded from the primary phylogenetic analysis because their addition creates a large polytomy leaving the majority of allosauroid taxa unresolved. The tetanuran theropod *Fukuiraptor*, a taxon recovered within Allosauroidea by some authors [25], [42], [48], [53,], is scored but not included in the primary or secondary analysis. Owing to a large percentage of missing data (91.5%) for *Fukuiraptor*, adding the taxon destroys nearly all resolution in the tree and collapses the sister taxon relationship of Coelurosauria and Allosauroidea. Although including problematic taxa with proposed allosauroid affinities that have little to no referred cranial material broadens the taxonomic sample, it creates a large polytomy at the base of Allosauroidea (Figure 52). This finding is consistent with the results from Holtz *et al*. [12], in which the inclusion of the same three taxa created a similar lack of resolution (Figure 33). In the present analysis, relationships among the following taxa collapse when *Australovenator*, *Fukuiraptor*, *Lourinhanosaurus*, and *Siamotyrannus* are included: Sinraptoridae (*Sinraptor* + *Yangchuanosaurus*), *Monolophosaurus*, *Allosaurus*, *Neovenator*, *Tyrannotitan*, and the unnamed subclade composed of *Acrocanthosaurus* + *Eocarcharia* and *Shaochilong* + Carcharodontosaurinae. More inclusive analyses have recently resolved the position of these taxa, recovering *Fukuiraptor* as either outside of Allosauroidea [10] or a member of Neovenatoridae [25], [42]. *Lourinhanosaurus* has been recovered as an allosauroid [10], but the affinities of *Siamotyrannus* are still uncertain.

## Discussion

### Homoplasy and character support

Although the topology of the recovered tree in the primary phylogenetic analysis (Figure 51) appears unaffected by missing data and homoplastic characters, a closer examination of character state distribution across Allosauroidea reveals a large amount of homoplasy. Distinguishing between underlying synapomorphies and potential autapomorphies becomes increasingly difficult with the amount of missing data present in this analysis; therefore, the majority of synapomorphies described below for the clades within Allosauroidea are unambiguously optimized. Future discovery of more complete specimens referable to ingroup taxa will potentially resolve these ambiguities.

Since the character matrix of the present analysis is supplemented by a large percentage (34.4%) of characters modified from Brusatte and Sereno [22], it is not surprising that a similar topology for Allosauroidea is recovered (Figures 33, 51). However, a more recent analysis by Brusatte *et al*. [37] that included *Shaochilong* recovered a different arrangement of carcharodontosaurid taxa than the present analysis. For example, Brusatte *et al*. [37] place *Tyrannotitan* in an unresolved polytomy with *Shaochilong* and Carcharodontosaurinae, whereas the present analysis recovers *Tyrannotitan* in a more basal position than the sister group *Acrocanthosaurus* + *Eocarcharia*, with *Shaochilong* as the single sister taxon to Carcharodontosaurinae. The present analysis also differs from a recent phylogeny produced by Benson *et al*. [42], in which *Tyrannotitan*, *Shaochilong*, *Acrocanthosaurus*, and *Eocarcharia* are recovered as successively more basal outgroups, respectively, to a polytomous Carcharodontosaurinae. Better understanding of the anatomy of these taxa (*e.g.*, *Tyrannotitan*, *Eocarcharia*, *Shaochilong*; Table S1) will likely resolve differences in their phylogenetic placement in this region of the tree.

In the present analysis, strong support is shown for the placement of *Acrocanthosaurus* well within Carcharodontosauridae, and *Acrocanthosaurus* is recovered as the sister taxon to *Eocarcharia*. The unnamed subclade of *Acrocanthosaurus* + *Eocarcharia* paired with *Shaochilong* + Carcharodontosaurinae is supported by one unambiguously optimized cranial synapomorphy: (character 90∶1) prominent medial flange at the dentary symphysis. Recovery of this group is also supported by ambiguously optimized synapomorphies (“*” indicates new characters originating from the re-evaluation of NCSM 14345), including: (17∶1) extensive external sculpturing covering the main body of the maxilla; (18∶1) dorsoventral depth of anterior maxillary interdental plates more than twice anteroposterior width; (30∶1) suborbital process along posterior margin of lacrimal ventral ramus; (31∶1*) deep sulcus along the anterior margin of the lacrimal ventral ramus; (32∶1*) lateral curvature of the lacrimal dorsal to the lacrimal recess; (40∶1*) vascular groove stretching across entire length of postorbital dorsal boss; (56∶1) medially pneumatized ventral shelf of pterygoid wing of quadrate; (60∶1*) opening for olfactory nerve exit split by mesethmoid; (79∶1*) ectopterygoid jugal ramus width less than 66% the width of the subtemporal fenestra; (80∶1) ectopterygoid with a single accessory foramen; (81∶1*) jugal process of ectopterygoid angled more than 15° dorsally with respect to main body; (82∶1*) jugal ramus of ectopterygoid rectangular in lateral view; (91∶2*) posterior and anterior foramina inset within lateral sulcus of the dentary; (94∶0) mylohyoid foramen of splenial completely enclosed. Missing data from *Eocarcharia*, *Shaochilong*, and *Tyrannotitan* have prevented the optimization of these cranial characters as unambiguous synapomorphies. All recovered members of this unnamed clade for which these characters could be evaluated (*e.g.*, *Acrocanthosaurus*, *Eocarcharia*, *Shaochilong*, *Carcharodontosaurus*, *Giganotosaurus*, *Mapusaurus*) share the same character states.

The phylogenetic placement of the taxon *Neovenator* from the Lower Cretaceous of Europe has also contributed to a lack of phylogenetic consensus regarding Allosauroidea [22]. Consistently recovered as a member of Allosauroidea (Figure 33), *Neovenator* has been placed as either a close relative to *Allosaurus* [13], [19] or a basal member of Carcharodontosauria [9], [12], [21], [22]. The present recovery of *Neovenator* as the basal-most member of Carcharodontosauria (Figure 51) supports the latter hypothesis, as do other recent analyses [37], [42], [75]. A relatively large Bremer support (8) for Carcharodontosauria is supported by eight unambiguously optimized synapomorphies, including: (character 4∶1) posterodorsal inclination of premaxilla anterior margin by at least 10°; (10∶1) solid medial wall of promaxillary recess; (21∶1) nasals of subequal width throughout length; (22∶0) flat lateral nasal margin lacking crest; (25∶0) nasal lateral recesses absent or reduced to small pits; (108∶1) pleurocoels on postaxial cervicals with multiple openings; (116∶1) pleurocoels present on all dorsals; and (169∶1) distal extent of lateral malleolus of tibia beyond that of the medial malleolus and 7% or more of the length of the tibia.

Less inclusive clades within Carcharodontosauridae are supported by ambiguously optimized synapomorphies, including several newly recognized characters from the re-description of NCSM 14345 (“*”). Assigning these features as synapomorphies for Carcharodontosauridae is contingent upon the discovery of more complete crania referable to *Neovenator* and *Tyrannotitan*, as missing data for these taxa preclude the ability to unambiguously optimize these new characters. Nevertheless, the current grouping of *Tyrannotitan*, *Eocarcharia* + *Acrocanthosaurus*, and *Shaochilong* + Carcharodontosaurinae shows high bootstrap (86) and Bremer support (3), and shares the following character states: (character 12∶1*) narrow separation between interfenestral and postantral struts of the maxilla; (13∶1*) sinuous medial ridge across interdental plates of the maxilla; (34∶1*) small accessory prong of the jugal between dorsal and ventral quadratojugal prongs; (35∶1) pronounced lateral ridge of the jugal overhanging posterior ramus of the maxilla; (38∶1) dorsal boss of postorbital extensively overhanging orbit; (39∶1*) presence of vascular groove across dorsal boss of postorbital; (42∶1*) expansion of supratemporal fossa limited to posterior margin of the main body of the postorbital; (43∶1) suborbital flange on ventral process of postorbital; (50∶1) fused frontal-frontal suture; (52∶1) fused frontal-parietal suture; (53∶1) frontal excluded from orbital rim by lacrimal-postorbital contact; (88∶1) dentary with squared and expanded anterior end; (93∶1) dentary symphysis U-shaped in dorsal view.

Similar to previous phylogenetic analyses of theropod relationships [12], [26], [105], homoplasy in the present analysis likely precludes the unambiguous optimization of several synapomorphies and/or autapomorphies. For example, as noted by Brusatte and Sereno [22], *Sinraptor* shares braincase characters (in this analysis, characters 69∶1; 70∶1) with *Carcharodontosaurus* and *Giganotosaurus* to the exclusion of *Allosaurus* and *Acrocanthosaurus*. It is suggested that these character states evolved independently in *Sinraptor* and Carcharodontosaurinae [22], and the topology of the present analysis supports this hypothesis. Similarly, character states scored from the pterygoid (77∶1; 78∶1; Figure 46) may have also been independently derived in *Sinraptor* and *Acrocanthosaurus*. In both taxa, the medial pterygoid process is rotated dorsally with respect to the vomeropalatine ramus, and the pterygoid itself is invaginated by fossae of the ectopterygoid and quadrate rami. Conversely, in *Allosaurus* the medial pterygoid process is approximately confluent with the vomeropalatine ramus, and no fossae are present. It is unclear whether or not *Sinraptor* and *Acrocanthosaurus* share these character states with other members of Allosauroidea, because pterygoids are either not well preserved or have yet to be described from any other allosauroid taxa. The only member of Carcharodontosauridae aside from *Acrocanthosaurus* with referred pterygoid material is *Giganotosaurus*, but the fragmentary and weathered nature of the pterygoid referred to this taxon (MUCPv-Ch1) precludes scoring of the above character.

**Figure 53:**
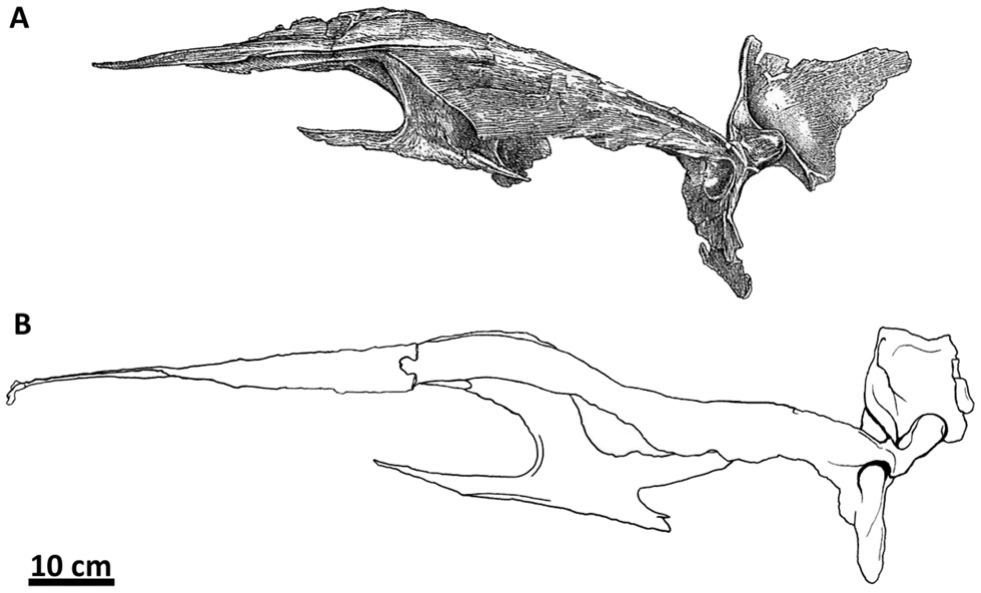
Similarity in palatal morphology between basally-positioned and derived allosauroid taxa. Palatal reconstructions of (A) *Sinraptor dongi* (image modified from [16]) and (B) *Acrocanthosaurus atokensis* (NCSM 14345).

In addition to the pterygoid, other elements of the palatal complex are nearly identical in *Sinraptor* and *Acrocanthosaurus* (*e.g.*, palatines, vomer; Figures 46, 53), although a lack of comparative material from other allosauroids is needed to fully test hypotheses of convergence. Differences between the ectopterygoids of these and other taxa may nevertheless underscore important transitions in the morphology of crania within Allosauroidea. For example, when compared to the carcharodontosaurids *Acrocanthosaurus* and *Giganotosaurus*, the jugal processes of the ectopterygoids of *Sinraptor* and *Allosaurus* are thicker and contact the jugal with a wide, triangular surface area. This corresponds to a transversely wider posterior region of the skull in *Sinraptor* than in *Acrocanthosaurus*; in *Acrocanthosaurus* and *Giganotosaurus*, the jugal process of the ectopterygoid is thinner and extended further laterally to contact the jugal with a narrow, rectangular surface, which creates a wide subtemporal fenestra. Furthermore, *Acrocanthosaurus* possesses a large opening between the palatine and pterygoid (the ‘pterygopalatine fenestra’) that is filled by a wall of bone in *Sinraptor* (Figure 46). While the skulls of carcharodontosaurid taxa became larger and heavier than those of more-basally positioned allosauroids (Table 6), the structure of the palate may have compensated by becoming narrower and more fenestrated. Basally-positioned allosauroids (*e.g.*, *Sinraptor*, *Allosaurus*) may not have required a lightened skull to the extent seen in carcharodontosaurids and therefore retained the ancestral condition of a broader palate with more robust elements (also see the abelisaurid *Carnotaurus* [81] and the megalosaurid *Dubreuillosaurus* [56]).

### Revised cranial diagnosis of *Acrocanthosaurus atokensis*

The most prominent diagnostic feature of material referred to the species *Acrocanthosaurus atokensis*, elongated neural spines along the vertebrae, influenced the etymology of its generic name and is shared by at least one other carcharodontosaurid (*Mapusaurus* [36]). However, all prior diagnoses of *Acrocanthosaurus atokensis* have relied upon several characteristics of the skull to distinguish it from other large theropods [1], [21], [23]. Description of the complete skull of NCSM 14345 has provided a better understanding of the comparative cranial anatomy of *Acrocanthosaurus atokensis*, thereby helping to modify its diagnosis. The following section includes a review and categorization of all cranial characters previously proposed to be diagnostic of the species *Acrocanthosaurus atokensis*. Several characters (and character states) are reviewed and excluded from the diagnosis due to their uninformative status, as are those that exhibit a broad distribution within Allosauroidea. New cranial characters from the reanalysis of the skull of NCSM 14345 that are found to be diagnostic are also addressed.

Characters shown to diagnose the species *Acrocanthosaurus atokensis* are placed in either primary or ancillary categories on the basis of their diagnostic strength. Primary characters have an autapomorphic character state unique to *Acrocanthosaurus atokensis* within Allosauroidea, or have an optimization such that the autapomorphic distribution of the character state is unlikely to change with new phylogenetic information. Ancillary characters contain character states more ambiguously supported as diagnostic of *Acrocanthosaurus atokensis*; these character states often have a wider, but nonubiquitous, distribution within Allosauroidea. Furthermore, ancillary characters may represent potential synapomorphies of larger clades within Allosauroidea upon the arrival of more complete phylogenetic information. Missing character states impose limitations on all diagnostic characters described in this section. For instance, the cranial material currently referred to the species *Eocarcharia dinops* lacks specific regions of the skull that cannot be scored for any primary or ancillary diagnostic characters. Given that *Eocarcharia dinops* is recovered as the sister taxon to *Acrocanthosaurus atokensis*, any new information concerning the skull of *Eocarcharia dinops* will influence the character state optimization and thus the diagnostic strength of the characters described herein.

Several cranial characters were proposed in the original diagnosis of *Acrocanthosaurus atokensis* [23] despite the fragmentary nature of the skull of the holotype specimen (see Table 2). Harris [21] reviewed and assigned numbers (2–6) to the cranial characteristics described by Stovall and Langston [23] in his description of *Acrocanthosaurus atokensis* specimen SMU 74646. Harris eliminated two characters as either arbitrary or excessively subjective, including: (2) proportionately massive skull and (3) moderately heavy bones surrounding orbital region [21]. Compared to smaller theropods (*e.g.*, *Coelophysis bauri*, *Compsognathus longipes* Wagner 1861 [120], *Herrerasaurus ischigualastensis*), ‘massive’ skulls are characteristic of every theropod currently referred to Allosauroidea (see Table 6). Large skulls are also widely distributed among the theropod groups Coelurosauria, Spinosauroidea, and Abelisauroidea. These facts combined strongly suggest that character (2) is not diagnostic of *Acrocanthosaurus atokensis*. Heavy bones surrounding the orbital region (3), an arbitrary character according to Harris [21], is also not recovered as diagnostic of *Acrocanthosaurus atokensis*. Robust elements (*i.e.*, postorbital, lacrimal, and jugal) enclose the orbital fenestra in *Acrocanthosaurus atokensis*, *Carcharodontosaurus saharicus*, *Giganotosaurus carolinii*, and *Mapusaurus roseae*. In addition, the postorbital of *Eocarcharia dinops* and the jugal of *Tyrannotitan chubutensis* are similar in overall scale and robustness to comparable elements in *Acrocanthosaurus atokensis*. Although more gracile skull bones surround the orbits of *Allosaurus fragilis*, *Monolophosaurus jiangi*, *Sinraptor dongi*, and *Yangchuanosaurus shangyouensis*, the distribution of (3) is widespread among carcharodontosaurids and the character is non-unique to *Acrocanthosaurus atokensis*.

The remaining four cranial characters (4–7) proposed by Stovall and Langston [23] were not addressed by Harris [21] due of a lack of comparative material associated with SMU 74646, and included: (4) orbits and postorbital fenestra somewhat reduced; (5) enlarged jugal pneumatic recess; (6) frontals and parietals solidly coossified; and (7) quadratosquamosal movement somewhat reduced. Review of character (4) finds that, aside from its subjective phrasing, it references a range of continuous variation within Allosauroidea and is not recovered as diagnostic of *Acrocanthosaurus atokensis*. The orbital and antorbital fenestrae are proportionally smaller than those of *Acrocanthosaurus atokensis* in the skulls of *Allosaurus fragilis*, *Monolophosaurus jiangi*, and *Yangchuanosaurus shangyouensis*. In *Sinraptor dongi* the orbit is proportional in size to *Acrocanthosaurus atokensis*, but the antorbital fenestra is relatively larger. Additionally, the skull of NCSM 14345 shows that the orbital fenestra of *Acrocanthosaurus atokensis* is proportionally smaller than the orbit reconstructed for the holotype specimen. Neither character (5) nor (6) is recovered as diagnostic of *Acrocanthosaurus atokensis*. Large jugal recesses and coossified frontals and parietals exhibit a broader distribution within Allosauroidea (See Appendix S1, character states 36∶1; 52∶1). For example, the jugal recess is enlarged in *Acrocanthosaurus atokensis*, *Carcharodontosaurus saharicus*, *Mapusaurus roseae*, *Monolophosaurus jiangi*, *Sinraptor dongi*, and *Tyrannotitan chubutensis*. This feature could not be evaluated for allosauroids without referred jugal material, specifically *Eocarcharia dinops*, *Giganotosaurus carolinii*, *Neovenator salerii*, and *Shaochilong maortuensis*. Specimens referred to *Acrocanthosaurus atokensis* share the presence of frontal and parietal coosification (6) with *Carcharodontosaurus iguidensis* Brusatte and Sereno 2007 [75], *Carcharodontosaurus saharicus*, *Eocarcharia dinops*, *Giganotosaurus carolinii*, and *Shaochilong maortuensis*. These elements are unfused in the more basally-positioned allosauroid taxa *Allosaurus fragilis* and *Sinraptor dongi*, and in *Monolophosaurus jiangi*. However, given its broad distribution within Carcharodontosauridae and shared presence in the sister taxon *Eocarcharia dinops*, character (6) is not diagnostic of *Acrocanthosaurus atokensis*. Missing quadrate material in the holotype specimen prevented a thorough assessment of character (7) in the original diagnosis of *Acrocanthosaurus atokensis*. Evidence for restricted quadratosquamosal movement includes a concave squamosal surface for articulation with the quadrate and development of exostotic material upon this surface [23]. The morphology of the squamosal articular surface for the quadrate is not diagnostic of *Acrocanthosaurus atokensis*, as this region is nearly identical in morphology to the squamosals of *Allosaurus fragilis* and *Sinraptor dongi*. Additionally, exostotic material between the quadrate and squamosal is not developed in NCSM 14345 (Figure 11), and its presence in the holotype specimen is likely a non-inheritable pathologic feature that is not ubiquitous within or unique to *Acrocanthosaurus atokensis*. Reassessment of the diagnostic nature of this character may be necessary upon the discovery of new data, because no squamosal material is yet known for any taxa within Carcharodontosauria aside from *Acrocanthosaurus atokensis*. A character listed in the diagnosis of *Acrocanthosaurus atokensis* by Currie and Carpenter [1], lacrimal-postorbital contact, is shown here to be nondiagnostic of *Acrocanthosaurus atokensis*; this feature is a synapomorphy of Carcharodontosauridae.

**Figure 54:**
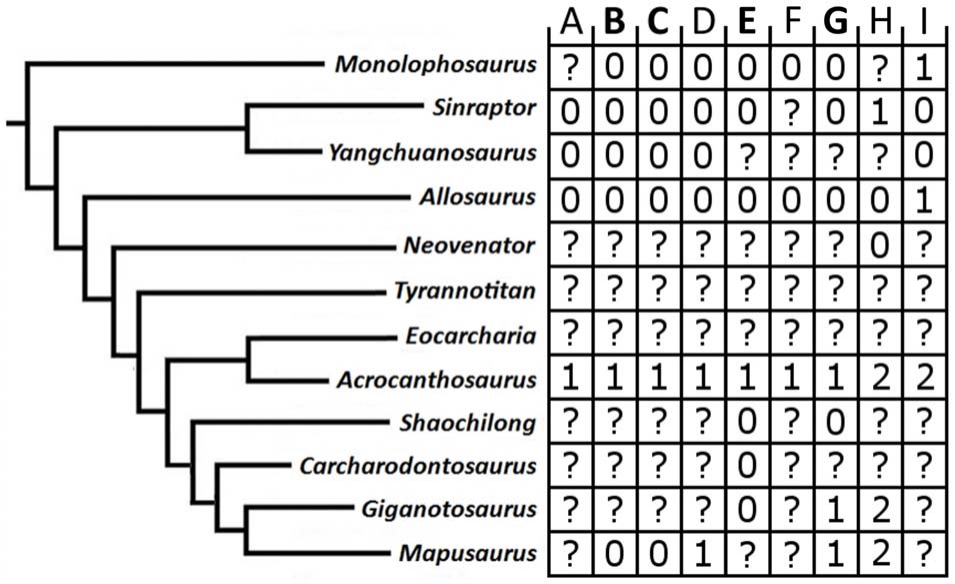
Distribution across Allosauroidea of primary and ancillary characters diagnostic of the species *Acrocanthosaurus atokensis*. Primary diagnostic characters are shown in boldface print. Refer to Appendix S2, Appendix S3, and Table S1 for character scorings.

Primary and ancillary diagnostic characters for the species *Acrocanthosaurus atokensis* are categorized as such on the basis of their diagnostic strength. The distribution of these characters across all allosauroid taxa is shown in Figure 54, with primary diagnostic characters in boldface. Four of these characters (lettered A–D) were proposed during the description of the fragmentary skull of SMU 74646 [21], including: (A) bifurcating jugal process of palatine; (B) pronounced knob on lateral surangular shelf; (C) enlarged posterior surangular foramen; and (D) reduced ridge dividing glenoid region of articular. From these, (B) and (C) are shown to be primary diagnostic characters of *Acrocanthosaurus atokensis*, while (A) and (D) are ancillary. Presence of a knob on the lateral surangular shelf (B) and an enlarged posterior surangular foramen (D) are unique to *Acrocanthosaurus atokensis* within Allosauroidea, and the absence of these character states in the carcharodontosaurid *Mapusaurus roseae* and several basal allosauroids (*e.g.*, *Allosaurus fragilis*, *Sinraptor dongi*) suggests that their current distributions are less likely to become synapomorphic of a larger clade (Figure 54). However, it is again cautioned that the absence of comparative material from *Eocarcharia dinops* limits the strength of both of these primary diagnostic characters. Bifurcation of the jugal process of the palatine (A) distinguishes *Acrocanthosaurus atokensis* from basal allosauroids that possess a solid jugal process of the palatine, but is categorized as ancillary because the character cannot be scored for any other allosauroid taxa. A reduced glenoid ridge (D) is also found to be an ancillary diagnostic character of the species *Acrocanthosaurus atokensis*. Although this character distinguishes *Acrocanthosaurus atokensis* from basally-positioned allosauroids, the carcharodontosaurian species *Mapusaurus roseae* is also scored for a reduced glenoid ridge. Therefore, (D) has an increased probability of being recovered as a synapomorphy of a larger clade within Allosauroidea upon the inclusion of more complete phylogenetic data.

Currie and Carpenter [1] identified one additional cranial character in their revised diagnosis of *Acrocanthosaurus atokensis*: (E) supraoccipital protruding as a double-boss on either side of the midline posterior to the nuchal crest. The presence of a double-boss on the supraoccipital (E) is recovered as a primary diagnostic character of *Acrocanthosaurus atokensis*. Basal carnosaurs (*e.g.*, *Allosaurus fragilis*, *Monolophosaurus jiangi*) and several carcharodontosaurids preserve a supraoccipital expressed as a single boss above the midline, and therefore the autapomorphic distribution of this character for *Acrocanthosaurus atokensis* is less likely to change with more complete phylogenetic data.

Newly recognized morphologies from the re-analysis of the skull of NCSM 14345 have resulted in four amendments (F–I) to the cranial diagnosis of *Acrocanthosaurus atokensis*. Presence of a deeply inset, septate pneumatic recess within the medial surface of the quadrate (G) is shown to be a primary diagnostic character of *Acrocanthosaurus atokensis* (Figures 10, 42, 54; Appendix S1, 59∶1). A medial quadrate recess is present, but non-septate in *Mapusaurus roseae*, and in *Giganotosaurus carolinii* and *Aerosteon riocoloradensis* this recess is non-septate and shallow. However, these taxa are scored for the same character state as in *Acrocanthosaurus atokensis*. Presence of an apneumatic medial surface of the quadrate in the carcharodontosaurid *Shaochilong maortuensis* supports the primary diagnostic status of (G), as it is currently more likely to be an autapomorphic character state for *Acrocanthosaurus atokensis* than a synapomorphy for Carcharodontosauria.

The new characters (F), (H), and (I) are recovered as ancillary diagnostic characters of the species *Acrocanthosaurus atokensis*. A squared postcotyloid process of the squamosal (F) is unique to *Acrocanthosaurus atokensis* (Figure 11A, 54; Appendix S1, 45∶2), because the postcotyloid process is rounded in basally-positioned allosauroids (*e.g.*, *Allosaurus fragilis*, *Sinraptor dongi*, *Monolophosaurus jiangi*). However, because no other allosauroid taxa have referred squamosal material, the distribution and diagnostic strength of this character state is likely to change with more complete data. A lateral sulcus is expanded across the entire length of the dentary of *Acrocanthosaurus atokensis* (H), a characteristic that also occurs in dentaries referred to the carcharodontosaurid species *Mapusaurus roseae* and *Giganotosaurus carolinii* (Figures 27A, 28, 49; Appendix S1, 91∶2). More data are needed to determine if this character is autapomorphic of *Acrocanthosaurus atokensis*, or a synapomorphy of a larger group within Allosauroidea. An expanded posterior mylohyoid foramen of the prearticular (I) is recovered as unique to *Acrocanthosaurus atokensis* (Figures 30, 50, 54; Appendix S1, 99∶2), but cannot be categorized as a primary diagnostic character at this time.

Reevaluation of previously proposed and new diagnostic characters has made it necessary to revise the formal diagnosis of the species *Acrocanthosaurus atokensis*. Four characters serve as primary diagnostic features, and include: a knob on the lateral surangular shelf, an enlarged posterior surangular foramen; the supraoccipital protruding as a double-boss posterior to the nuchal crest; and a pneumatic recess within the medial surface of the quadrate. Ancillary characters may have a greater diagnostic potential upon the availability of more complete phylogenetic data, but are currently limited in their strength. These five characters include: a bifurcating jugal process of the palatine; a reduced ridge dividing glenoid region of articular; a squared postcotyloid process of the squamosal; an anteroposteriorly expanded lateral sulcus on the dentary; and an enlarged mylohyoid foramen of the prearticular.

### Stratigraphic Consistency

Measures of stratigraphic fit are often used as an independent source of comparison among competing phylogenetic hypotheses. The fit of a given phylogeny to the stratigraphic record integrates the age of appearance of the terminal taxa (based on the geologic age of the fossils referred to those taxa) with the order of successive branching events implied by the structure of the phylogenetic tree [121]. When stratigraphic fit metrics are calculated for competing tree topologies that share the same taxa (usually pruned phylogenetic trees), support is shown for one tree versus another if the differences between their stratigraphic fit metrics are statistically significant [122]. Such metrics have been previously calculated for Allosauroidea [22], and the present study attempts to update these metrics using newer methods and phylogenetic data.

In order to determine the fit of the recovered phylogeny (Figure 51) to the fossil record, stratigraphic fit metrics are calculated and compared to those from two major, competing systematic analyses of Allosauroidea (although see [22] for an exhaustive analysis that compares the stratigraphic consistency of several other phylogenies). The first, that of Benson *et al.* [42] similarly recovers *Acrocanthosaurus* within Carcharodontosauridae, but presents a different topology than that of the present analysis (Figure 33). The second comparison is made with the phylogeny of Smith *et al*. [13] that recovers *Acrocanthosaurus* as more closely related to *Allosaurus* near the base of Allosauroidea, with both taxa excluded from Carcharodontosauria. As a matter of convenience, these two analyses will be referred to as BEN (for Benson *et al*. [42]) and SET (for Smith *et al*. [13]) from this point forward, and the present phylogeny will referred to as EAC (for Eddy and Clarke). EAC, BEN, and SET each recover different topologies and include taxonomic samples of different sizes (18, 15, and 56, respectively). In order to avoid biases associated with sample size [121]–[123], each tree is pruned to their shared taxa. Comparisons between EAC and BEN are made using the absolute age ranges of 9 taxa (*i.e.*, *Allosaurus*, *Neovenator*, *Acrocanthosaurus*, *Shaochilong*, *Tyrannotitan*, *Eocarcharia*, *Carcharodontosaurus*, *Mapusaurus*, and *Giganotosaurus*), while comparisons between EAC and SET are made using a slightly different assemblage of 8 shared taxa (*i.e.*, *Monolophosaurus*, *Allosaurus*, *Sinraptor*, *Neovenator*, *Acrocanthosaurus*, *Carcharodontosaurus*, *Tyrannotitan*, and *Giganotosaurus*).

The computer program ASCC (Assistance with Stratigraphic Consistency Calculations) generates measures of stratigraphic fit for EAC and SET (Table 5), including GER Range and MSM* Range [124]. Although GER (Gap Excess Ratio) is usually calculated as a single value using the maximum, midpoint, or minimum age of first appearance [125], ASCC generates a range of GER values, as well as MSM* Range (modified Manhattan Stratigraphic Measure). To compute these metrics, minimum and maximum ages of first appearance are entered into ASCC for each taxon, as well as Newick notations of EAC, BEN, and SET tree structures. Absolute ages for each epoch or stage are taken from the current International Stratigraphic Chart [126] and include errors associated with upper and lower bounds. The ASCC output file is analyzed using the maximum parsimony optimality criterion in TNT for 10,000 replications [116]. TNT then outputs a text-based file from which GER Range and MSM* Range are obtained. Values of MSM*, as well as their associated *p*-values, are calculated for maximum ages of first appearance in PAUP* [117] with input from files generated following the Pol and Norell [127] methodology. SCI values (Stratigraphic Consistency Index) [128] are calculated using the program Ghosts v.2.4 [125].

**Table 5:** Stratigraphic consistency metrics for the present analysis (EAC), and the analyses of Benson *et al*. (BEN) [42] and Smith *et al*. (SET) [13].

Analysis	GER Range	MSM* Range	MSM* (First Appearance)	*p* (MSM*)	SCI
EAC - Full analysis	1.00 – 0.76	1.00 – 0.59	1.00	<0.050	1.0
EAC - Cropped to 9 shared taxa with BEN	1.00 – 0.91	1.00 – 0.74	1.00	<0.050	1.0
BEN – Cropped to 9 shared taxa with EAC	1.00 – 0.66	1.00 – 0.38	1.00	<0.050	1.0
EAC – Cropped to 8 shared taxa with SET	1.00 – 0.90	1.00 – 0.76	1.00	<0.050	1.0
SET – Cropped to 8 shared taxa with EAC	0.80 – 0.54	0.60 – 0.38	0.45	0.185	0.6

**Figure 55:**
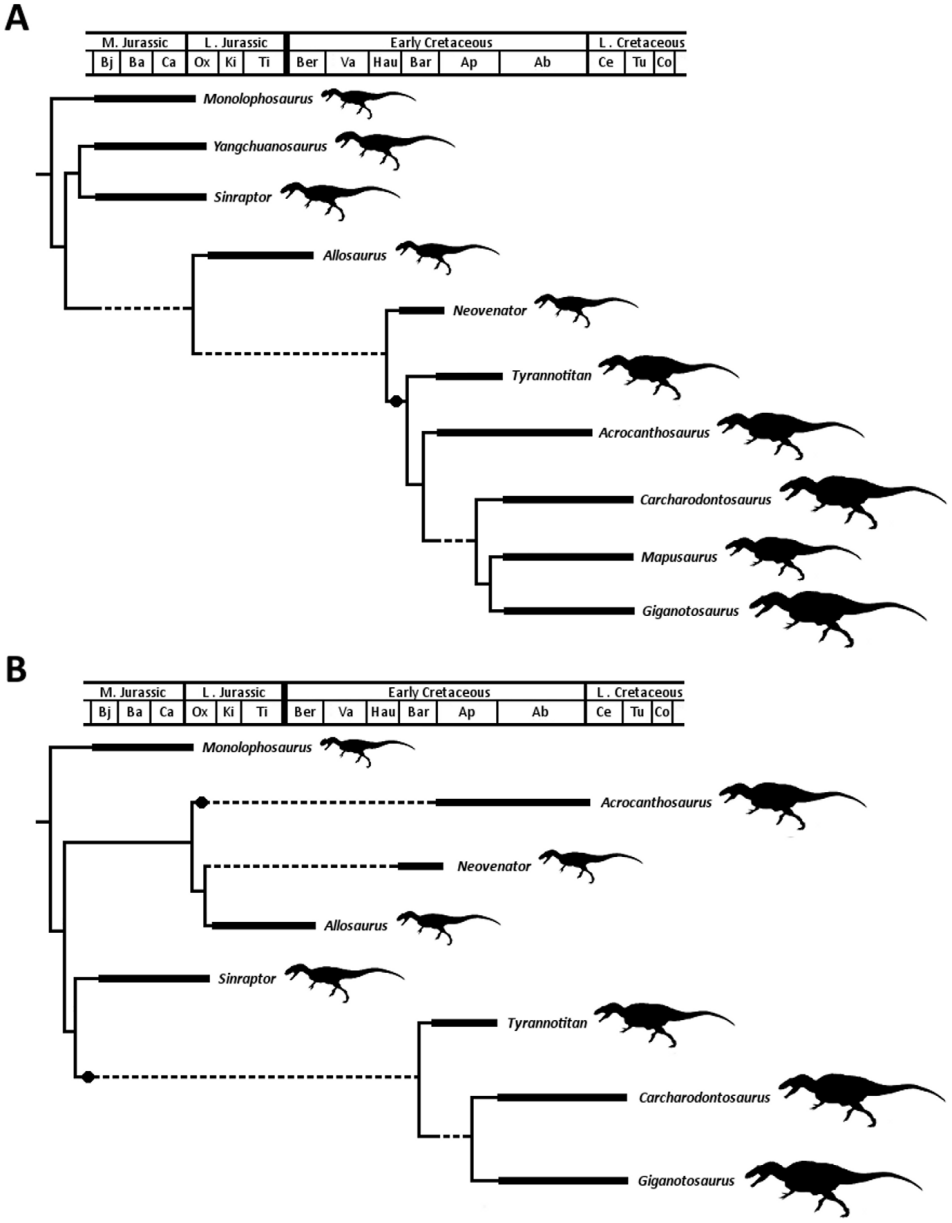
Phylograms and comparisons of body size optimization across Allosauroidea. Constructed from the trees recovered by (A) EAC, the present analysis, and (B) SET, Smith *et al*. [13]. Silhouettes are to scale according to measurements listed in Table 6.

Results from the stratigraphic consistency analyses for EAC, BEN, and SET are presented in Table 5. GER Range and MSM* Range are significantly higher for EAC than for SET. Ranges are also generally higher for EAC than BEN, but this difference is not statistically significant. SCI and MSM* values are greater for EAC than for SET. Furthermore, EAC is significantly more congruent with the stratigraphic record at the *p*<0.050 level; the MSM* value for SET (*p*  =  0.185) was not found to be significant at this level. Phylograms (Figure 55) constructed by combining each phylogeny with the reported epochs or stages of first appearance of their shared taxa (Table S2) confirm these numerical differences visually: EAC matches the stratigraphic record well, while the SET phylogram requires several sizable ghost lineages. It is not necessary to compare EAC and BEN with a phylogram, as the differences between the GER-Range and MSM*-Range for the two datasets are not statistically significant.

The stratigraphic fit metrics presented above suggest that the pruned topology of the present analysis is significantly more congruent with the stratigraphic record than that of Smith *et al*. [13]. Contributing to this difference in stratigraphic fit is the placement of *Acrocanthosaurus atokensis* within Allosauroidea. Smith *et al*. [13] recover *Acrocanthosaurus*, known from the Aptian-Albian of the Early Cretaceous, as closely related to the Late Jurassic *Allosaurus*, requiring sizable ghost lineages to construct this region of the tree (Figure 55B). Placement of *Acrocanthosaurus* among carcharodontosaurid taxa of similar age requires noticeably fewer ghost lineages, a result independently recovered by a previous analysis of stratigraphic fit to allosauroid phylogenies [10].

### Distribution of Body Size

The importance of body size of a related group of organisms should not be overlooked, as body size influences and is, in turn, affected by evolution, ecology, development, physiology, and reproductive strategy [83], [129], [130]. Charting trends in body size across competing phylogenies can provide an additional means of comparison, and potentially elucidate how a given group of organisms responds to evolutionary or environmental pressures [131]–[132]. The fact that there is a noticeable discrepancy in body size across Allosauroidea from basally-positioned, moderately-sized allosauroids (*e.g.*, *Allosaurus*, *Sinraptor*, *Neovenator*) to the derived, extremely large-bodied carcharodontosaurians (*e.g.*, *Acrocanthosaurus*, *Giganotosaurus*, *Carcharodontosaurus*) that include some of the largest known land predators [35] permits Fitch optimization of body size across competing phylogenies. The analysis described here presents a relatively coarse approach to visualizing trends in body size; a more rigorous quantitative analysis that incorporates all known allosauroid taxa is needed to further explore body size evolution within the group (although see [133]).

Body size reconstructions for specimens referred to taxa within Allosauroidea are taken from the literature and reported for each taxon in Table 6. Relative size is charted across the recovered phylogram (Figure 55) according to the methods demonstrated by Turner *et al*. [132]. Both the topology recovered by EAC and SET are compared visually. Fitch optimization [134] of the discrete character state of “larger body size” is performed on the trees of EAC and SET. As with comparisons of stratigraphic fit, differences between the Fitch of optimization body size across pruned trees of BEN and EAC are minimal, and therefore BEN is not figured. Note that while all known allosauroid taxa are relatively large predators, the terms “larger-bodied” is distinguished from “smaller-bodied” by total body lengths, skull lengths, and femur lengths greater than 10 m, 1 m, and 1 m, respectively.

**Table 6:** Measurements indicative of body size (Fig. 55) for twelve allosauroid taxa.

	Total Body Length (cm)	Skull Length (cm)	Femur Length (cm)
*Monolophosaurus jiangi*	510	67.0	-
*Yangchuanosaurus shangyouensis*	790	78.0	85.0
*Sinraptor dongi*	900	90.0	87.6
*Allosaurus fragilis*	970	100.8	88.0
*Neovenator salerii*	750*	70.0*	75.0
*Tyrannotitan chubutensis*	1220*	-	140.0
*Eocarcharia dinops*	800*	98.0*	-
*Acrocanthosaurus atokensis*	1150*	129.0	109.0
*Shaochilong maortuensis*	-	-	-
*Carcharodontosaurus saharicus*	1279	160.0*	126.0
*Mapusaurus rosae*	1260*	-	-
*Giganotosaurus carolinii*	1320*	195.0*	143.0

Abbreviation:

*, estimated measurement.

Maximum published values for each taxon are shown. Measurements for Eocarcharia dinops were estimated as one-half the linear dimensions of the derived carcharodontosaurid Giganotosaurus carolinii in accordance with Brusatte and Sereno [22]. Measurements for Shaochilong maortuensis are not estimated by Brusatte et al. [37], although the taxon is interpreted to be smaller than most allosauroids given that the length of its maxilla is 40% that of Acrocanthosaurus atokensis and Carcharodontosaurus saharicus and 75% the length of Allosaurus fragilis. Other publications providing measurements are listed in Table S1 and Appendix S3.

Comparing the two trees (Figure 55) suggests that EAC is more parsimonious than SET with respect to Fitch optimization of large body size. For example, if *Acrocanthosaurus* is placed with *Allosaurus* and *Neovenator*, as it is with SET, two separate acquisitions of large body size are implied (or one acquisition of large body size followed by one reversal to smaller body size). Conversely, placing *Acrocanthosaurus* within Carcharodontosauria implies only a single acquisition of large body size. This discrepancy in parsimony holds true even upon the addition of two “larger-bodied” allosauroid species not figured in Table 6 (*Allosaurus maximus* [135], *Yangchuanosaurus magnus* Dong, Chang, Li, and Zhao 1983 [73]). If these taxa were to replace the species exemplars “*Allosaurus*” and “*Yangchuanosaurus*” in the tree recovered by EAC, the number of times independent evolution of large size is optimized within Allosauroidea increases to at least 3: one acquisition of large body size for *Yangchuanosaurus magnus*, the second for *Allosaurus maximus*, and a third acquisition within Carcharodontosauria. However, the more parsimonious optimization of body size still favors placement of *Acrocanthosaurus* within Carcharodontosauria. If *Acrocanthosaurus* were the sister taxon to a *Neovenator salerii* + *Allosaurus maximus* clade as with SET, either two independent gains of large body size (one for *Acrocanthosaurus atokensis* and one for *Allosaurus maximus*) or an added reversal to small body size would be necessary, raising the total changes in allosauroid body size to 4.

### Paleobiogeography

Previous attempts to reconstruct the global distribution and dispersal routes of Allosauroidea across time were complicated by the recovery of *Acrocanthosaurus* as a derived member of Carcharodontosauria [12], [20]–[22]. Particularly, it was problematic to explain nesting of a North American taxon (*e.g.*, *Acrocanthosaurus*) within a carcharodontosaurid clade with a largely Gondwanan distribution (*e.g.*, *Eocarcharia*, *Carcharodontosaurus*, *Giganotosaurus*, *Mapusaurus*), considering the two landmasses were isolated by the time that these taxa first appear in the fossil record [12], [20]. For this reason, paleobiogeographical distributions were once thought to weaken the hypothesis that *Acrocanthosaurus* was more closely related to carcharodontosaurids, as no likely connection between North America and Gondwana was thought to have existed after the Jurassic [1], [12].

Paths for dispersal between North America, Europe, and Gondwana during the Early Cretaceous did exist; unfortunately the allosauroid fossil record during this time is poor, and thus makes testing hypotheses of biogeography and timing of dispersal events difficult. It has recently been suggested, however, that paleobiogeographic dispersal routes may have opened between Gondwana and Europe near the Barremian-Aptian boundary at 125 Ma. [136]. This proposal was based largely on the occurrence of rebbachisaurid sauropods and spinosauroid theropods from the Hauterivian-Barremian of Europe, two groups that are also known from the Aptian of Gondwana [96], [137]–[138]. Canudo *et al.* [136] suggest that a land bridge may have connected what is now the Apulia region of Italy to Africa via a series of microplates in the Early Cretaceous. Based on the presence of abundant shallow carbonate shelves, this region is interpreted to have undergone periods of emersion that coincide with eustatic depressions of global sea levels of up to 100 m [139]. The discovery of sauropod footprints in the Apulia region during this time supports its potential use as a thoroughfare [136].

The biogeographical hypothesis of Canudo *et al*. [136] is also congruent with the fossil record of Allosauroidea and may help explain the close phylogenetic relationship between *Acrocanthosaurus* and Gondwanan carcharodontosaurians. During the Valanginian (143.2 – 133.9 Ma), Europe and North America were still at least partially connected via present-day Greenland and a series of small islands [138]. Specimens of *Neovenator* from Europe, taken with the recovery of this taxon as the most basally-positioned member of Carcharodontosauridae, suggest that the common ancestor of Carcharodontosauridae likely inhabited Europe before the start of the Barremian (131.5 Ma), or lived elsewhere and dispersed to Europe before the Barremian. The ancestor of Carcharodontosauria could have easily been present in Europe before the earliest appearance of that group in the fossil record (*Neovenator*, 131.5–124.0 Ma), and then dispersed both southward into Gondwana giving rise to *Eocarcharia* and *Tyrannotitan*, eastward into Asia giving rise to *Shaochilong* [37], and westward into North America to give rise to *Acrocanthosaurus* by the Aptian. The recent discovery of the basal carcharodontosaurian *Concavenator* from the Upper Barremian of Spain [25] lends further support to this biogeographic hypothesis. Harris [21] recognized the possibility of a distribution route through Europe for the ancestor of *Acrocanthosaurus*. Sereno *et al*. [20] also proposed that ancestors to the Gondwanan carcharodontosaurids *Carcharodontosaurus* and *Giganotosaurus* became globally ubiquitous during the Early Cretaceous; similarly, this cosmopolitan distribution for Allosauroidea has emerged as a common characteristic of concurrent faunas [37], [42], [72], [76].

Although allosauroids continued to diversify after the Early Cretaceous, as evidenced by the presence of *Giganotosaurus*, *Mapusaurus*, and *Carcharodontosaurus* in the early Late Cretaceous of Africa and South America, the North American allosauroid fossil record is poorly sampled following the most recent stratigraphic occurrence of *Acrocanthosaurus*. Nevertheless, mid-Cretaceous North American sediments should not be ruled out as potential sources for undiscovered allosauroids, despite previous reports of their paucity [140]. Inadequate sampling also pervades sections of the allosauroid fossil record of Europe, Asia, and Antarctica, and Australia. Globally, poorly explored regions with contemporaneous terrestrial strata should also produce new allosauroid specimens. For example, although parts of Asia have yielded a solid record of basally-positioned allosauroids (*i.e.*, *Sinraptor*, *Yangchuanosaurus*, and *Monolophosaurus*), more complete specimens of enigmatic taxa with allosauroid affinities from Asia (*Siamotyrannus* and *Fukuiraptor*), Europe (*Lourinhanosaurus* and *Concavenator*), Australia (*Australovenator*), and Africa (*Afrovenator*) are needed to better understand the phylogenetic relationships within Allosauroidea and elucidate the paleobiogeographic patterns of this group.

### Conclusions

A re-evaluation of the skull of *Acrocanthosaurus atokensis* specimen NCSM 14345 has prompted an emended diagnosis of the species and brought new characters to bear on allosauroid phylogenetic relationships. This analysis has thoroughly supplemented prior descriptions of the taxon [1], [21], [23] and highlighted several newly recognized morphological features of *Acrocanthosaurus*, many of which may suggest structural re-organization of the allosauroid skull that have accompanied trends of increased size through time. Furthermore, many new characters may potentially represent unambiguously optimized synapomorphies of Carcharodontosauria (or less inclusive clades therein) upon the availability of more complete phylogenetic data.

Systematic analysis of Allosauroidea strongly supports the placement of *Acrocanthosaurus* as a nested member of Carcharodontosauria, removed from a sister taxon relationship with *Allosaurus* by 41 steps (Figure 51). Low Bremer and bootstrap support for internodes within Carcharodontosauria (*e.g.*, the relationships between *Eocarcharia* and *Tyrannotitan*) suggest that more complete phylogenetic data from poorly-known taxa will likely change relationships within this clade. Similarly, discoveries of new specimens referable to taxa lacking any cranial data (*e.g.*, *Siamotyrannus*, *Lourinhanosaurus*, *Fukuiraptor*) are necessary to better approximate their systematic placement within Theropoda.

Recovery of an allosauroid topology placing *Acrocanthosaurus* within Carcharodontosauridae also retains a significantly better fit to the fossil record than phylogenies that group *Acrocanthosaurus* with *Allosaurus* (Figure 55). Accordingly, the current analysis is more robustly supported by stratigraphic metrics and produces a phylogram with substantially shorter and fewer ghost lineages. The acquisition of large body size is also more parsimoniously optimized with the recovered phylogeny. Reconstructions of the paleobiogeography of allosauroid taxa support an emerging understanding of the cosmopolitan distribution of Early Cretaceous terrestrial faunas, and therefore strengthen the hypothesis of recovering the North American taxon *Acrocanthosaurus* within a group of Gondwanan carcharodontosaurids.

## Supporting Information

Table S1 **Operational taxonomic units, referred taxa, and sources for character state scorings.**
(DOC)

Table S2 **Stratigraphic occurrence and geologic age ranges for 12 allosauroid taxa used in stratigraphic consistency analysis.**
(DOC)

Appendix S1 **Description of characters in phylogenetic analysis.**
(DOC)

Appendix S2 **Data matrix.**
(DOC)

Appendix S3 **Supplemental references.**
(DOC)
