# Supplementary material for: New Information on the Cranial Anatomy of Acrocanthosaurus atokensis and Its Implications for the Phylogeny of Allosauroidea (Dinosauria: Theropoda)
Source: PLoS One. 2011 Mar 21;6(3):e17932. doi: 10.1371/journal.pone.0017932 (PMC3061882; doi:10.1371/journal.pone.0017932)
Supplement: Table S1 — Operational taxonomic units, referred taxa, and sources for character state scorings. (DOC) [file pone.0017932.s001.doc]

**Table S1.** Operational taxonomic units and percentages of missing data of taxa incorporated in the primary and secondary systematic analyses, taxa referred to those operational taxonomic units, and consulted sources for character state scorings.

|  | **Missing Data (%)** | **Referred Taxa** | **Consulted Publications** | **Specimens Examined and Image Sources** |
| --- | --- | --- | --- | --- |
| ***Herrerasaurus*** | 15.9 | *Herrerasaurus ischigualastensis*^†^ | [67,88,142] | N. Smith (of PVSJ 407) |
| **Coelophysoidea** | 18.2 | *Coelophysis bauri, Coelophysis rhodesiensis, Zupaysaurus rougieri* | [89,109,143] |  |
| ***Monolophosaurus*** | 44.9 | *Monolophosaurus jiangi* | [71-72,144-145] |  |
| ***Yangchuanosaurus*** | 81.9 | *Yangchuanosaurus shangyouensis, Yangchuanosaurus magnus* | [45,145] |  |
| ***Sinraptor*** | 9.7 | *Sinraptor dongi, Sinraptor hepengensis* | [16,146] |  |
| ***Allosaurus*** | 1.1 | *Allosaurus fragilis*^*^*, Allosaurus jimmadseni*^*^*, Allosaurus europaeus, Allosaurus maximus^*^* | [19,27,69,102,135,147] | BYU 571/8901, 683/9466, CM 1254,11843, OMNH 780, UUVP (multiple) |
| ***Neovenator*** | 57.6 | *Neovenator salerii*^†^ | [54,74,87] | R. Benson (of MIWG 6348) |
| *Siamotyrannus* | 93.2 | *Siamotyrannus isanensis* | [148] |  |
| *Lourinhanosaurus* | 85.8 | *Lourinhanosaurus antunesi* | [46] |  |
| *Fukuiraptor* | 91.5 | *Fukuiraptor kitadaniensis* | [53] |  |
| ***Tyrannotitan*** | 80.2 | *Tyrannotitan chubutensis*^†^ | [39] | F. Novas (of MPEF-PV 1156) |
| *Australovenator* | 87.0 | *Australovenator wintonensis* | [48] |  |
| ***Acrocanthosaurus*** | 8.5 | *Acrocanthosaurus atokensis*^*^ | [1,21,23,91] | NCSM 14345, OMNH 10146, 10147, SMU 74646 |
| ***Eocarcharia*** | 87.0 | *Eocarcharia dinops*^*^ | [49] | MNN GAD2 |
| ***Shaochilong*** | 81.4 | *Shaochilong maortuensis* | [37,76] |  |
| ***Carcharodontosaurus*** | 52.5 | *Carcharodontosaurus saharicus*^*^*, Carcharodontosaurus iguidensis*^*^ | [20,22,43-44,149-150] | SGM-Din 1, MNN IGU2 |
| ***Mapusaurus*** | 68.4 | *Mapusaurus roseae*^†^ | [36] | J. Canale & R. Benson, (of MCF-PVPH 108) |
| ***Giganotosaurus*** | 33.3 | *Giganotosaurus carolinii*^†^ | [35,41,104] | J. Canale & R. Benson, (of MUCPv-CH-1) |

**TABLE S1. (continued)**

|  | **Missing Data %** | **Referred Taxa** | **Consulted Publications** | **Specimens Examined and Image Sources** |
| --- | --- | --- | --- | --- |
| **Compsognathidae** | 52.8 | *Compsognathus longipes, Huaxiagnathus orientalis, Sinosauropteryx prima* | [110,120,151-155] |  |
| ***Tyrannosaurus*** | 21.5 | *Tyrannosaurus rex^*^* | [27,82,90,101] | UMNH VP 16690 |
| ***Dilong*** | 56.8 | *Dilong paradoxus* | [77] |  |
| ***Piatnitzkysaurus*** | 74.5 | *Piatnitzkysaurus floresi*^†^ | [108] | N. Smith (of PVL 4073) |

Taxa in the primary phylogenetic analysis are in bold. For references 142-155, see supplemental references in Appendix S3.

*Abbreviations*: ^*^, taxa with specimens examined by authors, ; ^†^, taxa scored from images provided by other researchers (see Acknowledgements). All other scorings relied on publications listed above (Appendix S1, S3).
