# Supplementary material for: New Information on the Cranial Anatomy of Acrocanthosaurus atokensis and Its Implications for the Phylogeny of Allosauroidea (Dinosauria: Theropoda)
Source: PLoS One. 2011 Mar 21;6(3):e17932. doi: 10.1371/journal.pone.0017932 (PMC3061882; doi:10.1371/journal.pone.0017932)
Supplement: Table S2 — Stratigraphic occurrence and geologic age ranges for 12 allosauroid taxa used in stratigraphic consistency analysis. (DOC) [file pone.0017932.s002.doc]

**Table S2.** Stratigraphic occurrence and geologic age ranges for 12 allosauroid taxa used in the stratigraphic consistency analysis.

|  | **Stratigraphic Occurrence(s)** | **Geologic Age(s) (Epoch)** | **Age Range (Ma.)** |
| --- | --- | --- | --- |
| *Monolophosaurus* | Wucaiwan Formation, China | **Bathonian – Callovian** (Middle Jurassic) | 171.2 – 157.2 |
| *Yangchuanosaurus* | Upper Shaximiao Formation, China | **Bathonian – Oxfordian** (Middle – Upper Jurassic) | 171.2 – 155.6 |
| *Sinraptor* | Shishugou Formation, China | **Bathonian – Oxfordian** (Middle – Upper Jurassic) | 171.2 – 155.6 |
| *Allosaurus* | Morrison Formation, USA; Lourinha Formation, Portugal | **Kimmeridgian – Tithonian** (Upper Jurassic) | 155.6 – 141.5 |
| *Neovenator* | Wessex Formation, England | **Barremian** (Lower Cretaceous) | 131.5 – 124.0 |
| *Eocarcharia* | Elrhaz Formation, Niger | **Aptian – Albian** (Lower Cretaceous) | 126.0 – 98.5 |
| *Shaochilong* | Ulansuhai Formation, China | **Aptian – Albian** (Lower Cretaceous) | 126.0 – 98.5 |
| *Acrocanthosaurus* | Antlers Formation, USA; Twin Mountains Formation, USA | **Aptian – Albian** (Lower Cretaceous) | 126.0 – 98.5 |
| *Tyrannotitan* | Cerro Barcino Formation, Argentina | **Aptian** (Lower Cretaceous) | 126.0 – 111.0 |
| *Carcharodontosaurus* | Bahariya Formation, Egypt; Tegana Formation, Morocco; Echkar Formation, Niger | **Albian – Cenomanian** (Lower – Upper Cretaceous) | 113.0 – 92.8 |
| *Mapusaurus* | Huincul Formation, Argentina | **Albian – Cenomanian** (Lower – Upper Cretaceous) | 113.0 – 92.8 |
| *Giganotosaurus* | Ro Limay Formation, Argentina | **Albian – Cenomanian** (Lower – Upper Cretaceous) | 113.0 – 92.8 |

Geologic and absolute ages are taken from the literature (see Appendix S1, Appendix S3, Table S1) and current International Stratigraphic Chart [126], respectively.
