# Supplementary material for: New Information on the Cranial Anatomy of Acrocanthosaurus atokensis and Its Implications for the Phylogeny of Allosauroidea (Dinosauria: Theropoda)
Source: PLoS One. 2011 Mar 21;6(3):e17932. doi: 10.1371/journal.pone.0017932 (PMC3061882; doi:10.1371/journal.pone.0017932)
Supplement: Appendix S1 — Description of characters in phylogenetic analysis. (DOC) [file pone.0017932.s003.doc]

**Appendix S1: Description of characters in phylogenetic analysis**

Characters modified from previous analyses were done so with respect to minor wording changes (“wording modified from”) or the actual character and character states (“modified from”). All characters presented as “new” were originally included in a Master’s thesis by Eddy [141], but this is the first formal publication of these new characters. However, three of the characters have been independently noted and published since that time.

**Cranial**

1. Premaxilla, number of teeth: three or four (0); five (1). ([22]:2; modified from [21]:47)
2. Premaxilla, lateral view, position of posterior end of tooth row: ventral to external naris (0); anterior to external naris (1). (wording modified from [105]:36)
3. Premaxilla, lateral view, ratio of height from external naris to alveolar margin relative to length of premaxillary alveolar margin: 0.5 to 1.25 (0); less than 0.5 (1); greater than 1.25 (2). (modified from [13]:5; [9]:1)
4. Premaxilla, lateral view, anterior margin immediately above alveolar rim, inclination from vertical: 0-9° (0); 10-20° (1). ([49]:2)
5. Premaxilla, subnarial posterior process: wide, plate-like, broadly contacting the nasals and excluding the maxilla from the external nares (0); strongly reduced in width, but still contacting the nasals (1); strongly reduced process does not contact the nasals, and the maxilla forms part of the posteroventral border of the external nares (2). (wording modified from [9]:6; [113]:2)
6. Maxilla, ventral margin of antorbital fenestra, position of the medial rim: lower than (0); or level with (1) lateral rim. ([22]:5; modified from [12]:36)
7. Maxilla, promaxillary fenestra, lateral exposure: absent, no fenestra (0); partially or fully exposed (1); present, but completely obscured by the lateral lamina of the ascending ramus (2). (modified from [22]:6; ordered)
8. Maxilla, maxillary fenestra, position relative to the anterior corner of the antorbital fossa: absent or anterior margin terminates posterior to the anterior margin of the antorbital fossa (0); terminates along the anterior margin of the antorbital fossa (1). ([22]:8; modified from [12]:43)
9. Maxilla, pneumatic excavation on the medial lamina of the ascending ramus: absent (0); present (1). ([22]:9; modified from [21]:2)
10. Maxilla, promaxillary recess, form of medial wall: recess absent or small (0); solid (1); fenestrated, open medially (2). ([22]:10; [56]:5)
11. Maxilla, dorsal view, orientation with respect to counterpart: acutely angled (0); subparallel (1). (wording modified from [12]:27)
12. Maxilla, posterior view, maximum separation between interfenestral and postantral struts: wider (0); or narrower (1) than the combined width of interfenestral and postantral struts. (new character; Figure 34)
13. Maxilla, medial view, shape of ridge across interdental plates: straight (0); sinuous (1). (new character, see also [10]:218; Figure 35)
14. Maxilla, medial view, anterior termination of palatal suture: anterior to distal margin of tooth seven (0); posterior to mesial margin of tooth eight (1). (new character; Figure 35)
15. Maxilla, curvature of posterior ramus: straight (0); curved, ventral deflection does not surpass ventral margin of largest maxillary tooth (1); curved, ventral deflection equal to or surpasses ventral margin of largest maxillary tooth (2). (modified from [12]:32; ordered)
16. Maxilla, medial view, fusion of interdental plates: absent (0); present (1). (wording modified from [40]:3)
17. Maxilla, external sculpturing, extent: absent, or present and restricted to the anterior and lateral margins above the tooth row (0); present, extensively covering the main body of the maxilla (1). ([22]:13; modified from [40]:11)
18. Maxilla, anterior interdental plates, dorsoventral depth: less (0) or more (1) than twice anteroposterior width. ([22]:15)
19. Nasal, lateral view, naso-maxillary process: absent (0); present (1); (new character; Figure 36)
20. Nasal, dorsal surface: low texture (0); heavily rugose (1). ([22]:16; modified from [40]:11; [9]:18)
21. Nasal, shape in dorsal view: expanding posteriorly so that lateral margins diverge (0); equal or subequal width throughout their length (1). (modified from [22]:17; [9]:21)
22. Nasal, lateral margin, form: flat (0); offset with a small lateral crest (1). ([22]:18; modified from [9]:22)
23. Nasal, participation in the antorbital fossa: nasal entirely excluded from the antorbital fossa (0); antorbital fossa reaches the nasomaxillary suture, but the lateral surface of the nasal is excluded from the antorbital fossa (1); lateral surface of the nasal participates in the antorbital fossa (2). (wording modified from [12]:53; ordered)
24. Nasal, shape of the posterior suture: medial projection extends as far or farther posteriorly than the lateral projection (0); lateral projection extends farther posteriorly than the medial projection (1). ([22]:19; wording modified from [12]:56)
25. Nasal, lateral view, size of recesses: absent or reduced to the size of small pits (0); present as large recesses (1). (modified from [12]:62; Figure 36)
26. Narial fossa, shape: ovular (0); highly elongated, with significant posterodorsal expansion onto nasal (1); posterior extent of narial fossa obscured by rugosity (2). (new character; Figure 36)
27. Lacrimal, lateral view, dorsal surface relative to skull roof: level with or slightly raised above skull roof (0); rugose, with a raised crest or a pronounced horn (1). (wording modified from [22]:20; [13]:52)
28. Lacrimal, length of anterior ramus: greater than 65% of the height of the ventral ramus (0); less than or equal to 65% of the height of the ventral ramus (1); anterior ramus strongly reduced and almost nonexistent (2). ([13]:56; [137]:2; ordered)
29. Lacrimal, shape and proportions of ventral ramus: broadly triangular, contact with jugal nearly twice as wide anteroposteriorly as the lacrimal at the junction between anterior and ventral rami (0); bar- or strut-like, roughly the same anteroposterior width throughout (1). (wording modified from [13]:59)
30. Lacrimal, suborbital process along posterior margin of ventral ramus: absent (0); present (1). (modified from [12]:79)
31. Lacrimal, anterior view, contact between lateral and medial plates of ventral ramus: present, plates fused (0); absent, deep sulcus along anterior margin (1). (new character; Figure 37)
32. Lacrimal, shape in dorsal view: straight (0); exhibiting a lateral curvature above the lacrimal recess (1). (new character; Figure 38)
33. Jugal, lateral view, relative heights of quadratojugal prongs: dorsal prong shorter or equal in height (0); or taller (1) than ventral prong. (new character; Figure 39)
34. Jugal, lateral view, small accessory prong between dorsal and ventral prong of quadratojugal process: absent (0); present (1). (new character; Figure 39)
35. Jugal, lateral ridge expressed along the ventral margin of the jugal: absent, smooth lateral surface (0); present, overhanging posterior ramus of the maxilla (1). (modified from [13]:49)
36. Jugal, lateral view, accessory pneumatization of the antorbital fossa: absent or shallow (0); extensive, invaginated recess (1). (modified from [22]:28; [21]:12)
37. Jugal, medial view, foramen present on medial surface ventral to postorbital process: absent (0); present (1). (wording modified from [13]:48; [1]:16)
38. Postorbital, dorsal boss, lateral expansion: absent or only slightly overhanging orbit (0); bulbous swelling extensively overhanging orbit (1). (wording modified from [22]:26;Figure 40)
39. Postorbital, vascular groove across dorsal boss: absent (0); present (1). (new character; Figure 40)
40. Postorbital, dorsal boss, extent of vascular groove: limited to anterior half of dorsal boss (0); present across entire dorsal boss (1). (new character; Figure 40)
41. Postorbital, ventral termination of ventral ramus: close to ventral margin of the orbit and ventral to squamosal-quadratojugal contact (0); dorsal to ventral margin of orbit and at same height or dorsal to squamosal-quadratojugal contact (1). (Modified from [21]:7)
42. Postorbital, dorsal view, expansion of supratemporal fossa onto main body (*i.e.*, excluding the squamosal process): closer to anterior margin of main body (0); closer to posterior margin of main body (1). (new character, see also [42]:47; Figure 41)
43. Postorbital, suborbital flange on ventral process, form: absent or indistinct (0); present as a discrete projection on the ventral ramus (1). ([22]:23; modified from [20]:49) (Figure 40)
44. Postorbital, cross-section of ventral process: triangular (0); U-shaped (1); ([13]:71; [20]:35)
45. Squamosal, lateral view, distal end of postquadratic (= postcotyloid) process: rounded and slightly tapering (0); squared and expanded (1). (new character; Figure 42)
46. Prefrontal: present as a distinct element (0); absent, lost or coossified with the lacrimal or frontal and not visibly distinct (1). ([22]:31) (Figure 38)
47. Prefrontal, medial view, shape of frontal articular surface: triangular (0); rounded (1). (new character; Figure 43)
48. Frontal, supratemporal fossa: absent (0); occupying the posterolateral third (1), occupying the posterolateral half (2); occupying most of the posterior frontal, meeting along the midline to form a frontal sagittal crest (3). ([12]:87; ordered)
49. Frontal, dorsal view, anteromedial corner of supratemporal fossa, open dorsally (0); roofed over by frontoparietal shelf (1). (wording modified from [40]:33; [41]:1)
50. Frontal, dorsal view, suture with counterpart: unfused (0); fused (1). (wording modified from [22]:34; [26]:41)
51. Frontal, dorsal view, relative length of associated frontals: longer than wide (0); as wide as (or wider than) long (1). (wording modified from [13]:63; [56]:21)
52. Frontal, dorsal view, suture with parietal: unfused (0); fused (1). (wording modified from [40]:38)
53. Frontal, contribution to the orbital rim: present (0); absent, excluded by lacrimal-postorbital contact (1). ([22]:36; modified from [20]:48)
54. Frontal, contribution to midline nasal crest: absent (0); present (1). (wording modified from [13]:64)
55. Parietal, posteriorly-placed knob-like dorsal projection, form: absent or very low (0); pronounced (1). ([22]:38; modified from [9]:42)
56. Quadrate, medial view, pneumatization of ventral shelf of pterygoid wing (= quadrate recess): absent (0); present (1). (modified from [9]:48; [56]:26)
57. Quadrate, posterior view, quadrate foramen: developed as a distinct opening between the quadrate and quadratojugal (0); almost entirely enclosed in the quadrate (1); absent (2). (wording modified from [13]:87; [9]:49)
58. Quadrate, ventral view, anterior most extension of jaw joint: only slightly posterior to (0); well posterior to (1); or anterior to (2) the posterior margin of the occipital condyle. (modified from [12]:132; ordered)
59. Quadrate, posterior view, pneumatic fossa dorsomedial to the quadrate foramen: absent (0); present (1). (new character; Figure 44)
60. Braincase, anterior view, exit for olfactory nerve (cranial nerve I): single opening (0); opening split medially by mesethemoid (1). (new character; Figure 45)
61. Braincase, participation of the supraoccipital in the dorsal margin of the foramen magnum: present (0); absent or reduced (1). (wording modified from [13]:96; [56]:45)
62. Braincase, supraoccipital, width of dorsal expansion: less than twice the width (0); or more than twice the width (1) of the foramen magnum. (wording modified from [13]:97; [41]:13)
63. Braincase, transverse distance across basal tubera: greater than width of occipital condyle (0); less than width of occipital condyle (1). (modified from [22]:39; [26]:97)
64. Braincase, angle between occipital condyle and basal tubera: perpendicular, at or near 90º (0); acute (1). (wording modified [13]:99; [41]:7).
65. Braincase, shape of occipital condyle: subspherical (0); dorsoventrally compressed (1). (wording modified from [41]:15)
66. Braincase, posteroventral view, neck of occipital condyle invaded by ventrolateral pair of pneumatic cavities that join medially: absent (0); present (1). (wording modified from [41]:6)
67. Braincase, ossification of the interorbital septum: absent (0); present (1). ([23]:43; modified from [41]:4)
68. Braincase, location of trigeminal foramen (cranial nerve V) relative to nuchal crest: anterior or ventral (0); posterior (1). (wording modified from [22]:44; [41]:2)
69. Braincase, form of trigeminal foramen (cranial nerve V) exit: single (0); fully split (1). (wording modified from [13]:108)
70. Braincase, pneumatic openings associated with the internal carotid artery: absent (0); present (1). (wording modified from [13]:106; [56]:23)
71. Braincase, basal tubera, composition: formed equally by the basioccipital and basisphenoid and not subdivided (0); subdivided by a lateral longitudinal groove into a medial part entirely formed by the basioccipital and a lateral part entirely formed by the basisphenoid (1). ([22]:46; modified from [9]:55; [20]:44)
72. Braincase, posterior view, paroccipital process: directed laterally, or slightly ventrolaterally (0); directed strongly ventrolaterally, with distal end entirely below the level of the foramen magnum (1). ([13]:90; modified from [12]:167; [9]:52)
73. Braincase, basisphenoid recess, form: absent or poorly developed (0); present between basisphenoid and basioccipital (1); present entirely within basisphenoid (2). ([13]:104; modified from [9]:57; ordered)
74. Braincase, basisphenoid recess, shape of posterior opening: single opening (0); divided into two small, circular foramina by a thin bar of bone (1). (wording modified from [13]:105)
75. Braincase, exposure of middle ear region in occipital view: absent (0); present (1). (wording modified from [41]:3)
76. Braincase, median ridge splitting exit of cranial nerve VI: present (0); absent (1). (wording modified from [41]:5)
77. Pterygoid, medial view, fossae penetrating the quadrate and ectopterygoid rami: absent (0); present (1). (new character; Figure 46)
78. Pterygoid, medial view, angle of medial process with respect to angle of vomeropalatine ramus: parallel or subparallel (0); rotated dorsally by at least 30º (1). (new character; Figure 46)
79. Ectopterygoid, dorsal view, ratio of anteroposterior width of jugal ramus to lateromedial width of subtemporal fenestra: greater than or equal to 0.66 (0); less than 0.66 (1). (new character; Figure 47)
80. Ectopterygoid, medial view, number of foramina: none (0); one (1); two (2). (wording modified from [12]:153).
81. Ectopterygoid, angle of jugal process with respect to main body of ectopterygoid: parallel or rotated dorsally less than 15º (0); non-parallel, dorsal rotation greater than or equal to 15º (1). (new character; Figure 47)
82. Ectopterygoid, lateral view, ventral margin of jugal process in relation to dorsal margin: ventrally expanded, giving jugal ramus triangular shape (0); parallel to dorsal margin, giving jugal ramus rectangular shape (1). (new character; Figure 47)
83. Epipterygoid, dorsal region, shape: rounded, bulbous tip (0); pointed tip (1). (new character; Figure 48)
84. Palatine, pneumatic recess, form: absent or small foramen (0); large fossa with one or more foramina (1). ([22];47; modified from [21]:33)
85. Palatines, medial contact: absent, separated by vomer and/or pterygoid (0); present (1). (wording modified from [12]:140)
86. Palatine, shape: subrectangular or trapezoidal (0); tetra-radiate (1); triradiate with no jugal processes (2). ([12]:141; [21]:29)
87. External mandibular fenestra, lateral view, maximum anteroposterior length of fenestra relative to length of mandible: greater than 15% (0); less than 15% (1). (modified from [21]:38)
88. Dentary, lateral view, shape of anterior end: rounded (0); squared and expanded (1). (wording modified from [22]:49; [20]:50)
89. Dentary, posterior end, form: strongly forked (0); slightly forked or straight (1). ([22]:55; modified from [9]:77)
90. Dentary, medial view, flange at anteroventral symphysis: absent or strongly reduced (0); prominent (1). (modified from [22]:50)
91. Dentary, expression of foramina on lateral surface: foramina expressed at the surface of the dentary (0); only posterior foramina inset within sulcus (1); posterior and anterior foramina inset within lateral sulcus (2). (new character, modified from [12]:217, see also [10]:80; Figure 49; ordered)
92. Dentary teeth, number: less than 25 (0); 25 to 30 (1); greater than 30 (2). (modified from [13]:127)
93. Dentary, symphysis, angle in dorsal view: low, acute angle, tooth row forms V-shaped convergence (0); high, obtuse angle, tooth row forms U-shaped convergence (1). ([22]:53).
94. Splenial, medial view, mylohyoid foramen: present, completely enclosed by splenial (0); present, opens anteroventrally (1); absent (2). (modified from [12]:239)
95. Angular, posterior termination: posterior or ventral to surangular foramen (0); anterior to surangular foramen (1). (wording modified from [12]:233)
96. Surangular, dorsoventral depth over the external mandibular fenestra: less than (0) or more than (1) half the depth of the mandible. ([22]:56; modified from [20]:47)
97. Articular, ridge dividing mandibular glenoid: pronounced (0); reduced (1). ([21]:41)
98. Articular, pendant medial process: absent (0); present (1). ([12]:246)
99. Prearticular, mylohyoid foramen: absent or fails to perforate anteroventral margin of prearticular (0); present, foramen height less than half dorsoventral height of prearticular above foramen midline (1); present, foramen height greater than half dorsoventral height of prearticular above foramen midline (2). (new character; Figure 50; ordered)

**Dentition**

1. Premaxillary teeth, shape of cross-sections: elliptical (0); subcircular (1); D-shaped (2). (wording modified from [13]:18; [114]:56)
2. Premaxillary teeth, labiolingual symmetry: symmetrical (0); asymmetrical (1). (wording modified from [13]:19)
3. Teeth, interdenticular sulci on lateral teeth: absent (0); present (1). (Benson, 2009:90; modified from [13]:18;)
4. Tooth crown recurvature: present (0); reduced or absent (1). ([137]:35; [10]:96)

**Axial Skeleton**

1. Axis, pleurocoel: absent (0); present (1). ([13]:142; [109]:74)
2. Axis, ventral keel: present (0); absent (1). ([21]:51)
3. Axis, ventral margin of the axial intercentrum, orientation relative to the ventral margin of the axial centrum: approximately parallel (0); angled strongly dorsally (1). ([22]:60; wording modified from [21]:50)
4. Cervicals, number of pleurocoels: two (0); one (1). ([13]:148)
5. Cervicals, pleurocoels on postaxial cervicals, form: absent or single opening (0); multiple openings within a single fossa (1). ([22]:65; modified from [21]:61)
6. Cervicals, vertebral centra: amphi-to platycoelous (0); strongly opisthocoelous (1). (wording modified from [13]:151)
7. Cervicals, posterior articular face of mid cervical centra, width: approximately as broad as tall (0); at least 20% broader than tall (1). ([22]:62; modified from [20]:53)
8. Cervicals, interior structure of centrum, pneumaticity: apneumatic or camerate (simple) (0); camellate (complex) (1). ([22]:64; wording modified from [21]:62)
9. Cervicals, location of the zygapophyses relative to the midline: over centrum (0); displaced lateral to centrum (1). ([22]:66; modified from [26]:155)
10. Mid-cervicals, length of vertebral bodies: about twice the diameter of the cranial face (0); more than two and up to four times or more the diameter of the cranial face (1); less than twice but more than half the diameter of the cranial face (2). (modified from [12]:309)
11. Mid-cervicals, anterior articular face of centra, orientation relative to posterior face: elevated (0); approximately at same level (1). (wording modified from [22]:63; [20]:54)
12. Anterior cervicals, shape of anterior articular facet: approximately as tall as wide or taller (0); significantly wider than tall (1). (wording modified from [13]:154)
13. Dorsals, distribution of pleurocoels: absent or restricted to the anterior dorsals (0); present on all dorsals (1). ([22]:67; modified from [21]:66)
14. Dorsals, height of neural spine relative to centrum height: less than (0); or more than (1) twice centrum height. ([22]:69; modified from [26]:167)
15. Dorsal vertebrae, neural spines: transversely compressed sheets (0); transversely broadened anteriorly and posteriorly and central regions of lateral surface embayed by deep, vertically oriented troughs (1). ([10]:119)
16. Dorsal vertebrae, centrum shape relative to mid section height: subcylindrical, the dorsoventral thickness of the central section greater than 60% the height of the cranial face (0); hourglass-shaped, the dorsoventral thickness less than 60% the height of the cranial face (1). ([22]:70; modified from [26]:175)
17. Posterior dorsal vertebrae, centrum height to length ratio: 1.0 to 2.5 (0); less than 1.0 (1); greater than 2.5 (2). ([13]:169; modified from [105]:48)
18. Anterior dorsal vertebrae, ventral keel, form: absent or very poorly developed (0); present (1). (wording modified from [13]:170; [9]:108)
19. Presacral vertebrae, pneumaticity: pleurocoels developed as deep and uninvaginated depressions (0); large chambers within centrum (camerate) (1); subdivided into sub-chambers (camellate) (2). ([10]:109; modified from [21]:62)
20. Sacral vertebrae, number: five (0); six (1). ([10]:125; modified from [102:121)
21. Sacral vertebrae, fenestrae between sacral neural spines: absent (0); present (1). ([10]:127)
22. Sacral vertebrae, pleurocoels: absent (0); present (1). (wording modified from [9]:115; [21]:70)
23. Caudal vertebrae, rudimentary pleurocoels: absent, or as shallow fossa (0); present (1). ([22]:72; modified from [20]:55)
24. Anterior caudal vertebrae, ventral surface: shallow groove (0); distinct sunken groove (1); robust ventral ridge (2). (wording modified from [9]:120; [10]:128)
25. Anterior caudal vertebrae, ventral surface: rounded (0), having a distinct keel bearing a narrow, shallow groove on its midline (1). (wording modified from [12]:362)
26. Mid-caudal vertebrae, spinous process, shape and angle: rodlike and posteriorly inclined (0); subrectangular and sheetlike (1). (modified from [12]:353).
27. Distal caudal vertebrae, length of prezygapophyses: more (0); or less (1) than 40% overlap of the preceding vertebral body. (wording modified from [22]:73; [26]:199)
28. Caudal vertebrae, number bearing transverse processes: 16 or more (0); fewer than 16 (1). (wording modified from [13]:191)
29. Caudal vertebrae, distribution of elongate prezygapophyses: caudal twenty-five (0); or caudal fifteen (1) and more distal caudal vertebrae. (wording modified from [12]:367)
30. Mid-caudal vertebrae, anterior margin of neural spines, form: straight (0); bearing a distinct kink, dorsal part of anterior margin inclined more posteriorly than ventral part (1). (wording modified from [13]:195; [9]:123)
31. Mid-caudal vertebrae, anterior spur in front of neural spine: absent (0); present (1). (wording modified from [13]:198)
32. Mid-caudal chevrons, shape: rod-like or slightly ventrally expanded (0); L-shaped (1). (wording modified from [13]:201; [20]:26)

**Pectoral Girdle and Forelimb**

1. Gastralia, distal end of medial element, shape: tapered (0); club-shaped prominence (1). ([22]:74)
2. Gastralia, number of sets of fused medial elements: zero or one (0); greater than one (1). ([22]:75)
3. Scapula, distal end, shape: expanded (0); not expanded relative to proximal portion of scapula (1). ([13]:213)
4. Scapula, acromion process, size: prominent (0); reduced or absent (1). ([23]:96; wording modified from [26]:213)
5. Scapula, blade length relative to minimum neck width: more (0) or less than (1) 7.5. ([23]:97; modified from [40]:63)
6. Scapulocoracoid, notch between scapular acromion process and coracoid: absent (0); present (1). ([22]:94; wording modified from [1]:68)
7. Coracoid, posteroventral process, length relative to the width of the glenoid: shorter (0); longer, process strongly hooked (1). ([22]:95; wording modified from [20]:28)
8. Radius, shape of radial external tuberosity and ulnar internal tuberosity: low and rounded (0); hypertrophied (1). ([13]:230; modified from [137]:33)
9. Metacarpals, proximal articular ends, transverse width: less than minimum transverse shaft width (0); two times or more than minimum transverse shaft width (1). ([22]:99)
10. Metacarpal I, length: greater that 50% metacarpal II length (0); less than 50% metacarpal II length (1). ([13]:234)
11. Metacarpal I, shape: longer than wide (0); approximately as wide as long (1). (modified from [13]:235; [9]:164)
12. Metacarpal IV: present (0); absent (1). ([22]:98; wording modified from [21]:100)
13. Manual ungual I: less than 50% the length of the radius (0); more than 50% the length of the radius (1). (modified from [13]:249)

**Pelvic Girdle and Hindlimb**

1. Ilium, anterodorsal rim, shape: convex or straight (0); concave (1). (wording modified from [13]:255; [9]:173)
2. Ilium, lateral wall of iliac brevis fossa: deeper than medial wall (0); shallower than medial wall (1); positioned ventral to medial wall (2). (wording modified from [13]:271; [114]:111)
3. Ischium, distal end, form: confluent with shaft or only slightly expanded (0); strongly expanded into distinct boot, with anterior and posterior projections (1). ([22]:81; modified from [21]:119)
4. Ilium, length to width ratio of pubic peduncle: 1 or lower (0); 1.3–1.4 (1); 1.55–1.75 (2); greater than 2.0 (3). ([10]:166; modified from [24]:28).
5. Ischium, posteriorly-directed flange on iliac peduncle: absent (0); present (1). ([22]:82)
6. Ischium, ventral notch between obturator-process: absent (0); present (1). (wording modified from [13]:289; [20]:12)
7. Pubis, obturator foramen: completely enclosed (0); open ventrally (1); absent (2). ([13]:276; modified from [17]:94)
8. Pubis, strongly expanded pubic boot: absent (0); present (1). ([13]:280; [21]:113)
9. Pubis, pubic boot size relative to pubic length: less than 50% (0); 50-60% (1); greater than 60% (2). ([22]:80; wording modified from [20]:51; ordered)
10. Femur, direction of femoral head: ventral (0); horizontal (1); dorsal (2). (wording modified from [13]:294; ordered)
11. Femur, lateral distal condyle, form: bulbous (0); cone-shaped (1). ([22]:84)
12. Femur, medial epicondyle (= mediodistal crest), length: poorly developed or short (0); pronounced, extending 30% or more up the length of the femoral shaft (1). ([22]:85; modified from [12]:574)
13. Femur, fourth trochanter, form: robust or completely absent (0); present but reduced to a weak crest (1). ([22]:86; modified from [21]:126)
14. Femur, ridge for cruciate ligaments in flexor groove, form: absent or indistinct (0); present and robust (1). ([22]:88; wording modified from [21]:128)
15. Femur, oblique ligament groove on posterior surface of head: absent or very shallow (0); deep, bound medially by a well-developed posterior lip (1). ([10]:189; modified from [9]:197).
16. Femur, muscle scar situated medially on anterior surface of distal femur: suboval rugose patch not extending to distal end of femur (0); large oval depression (1). ([10]:194)
17. Distal femur, broad groove on cranial surface: absent or poorly developed (0); well developed and bound medially by an expanded medial lamella (1). ([13]:302; modified from [9]:202; [40]:86)
18. Tibia, lateral condyle: confluent with cnemial crest anteriorly in proximal view (0); strongly offset from cnemial crest by incisura tibialis (1). ([9]:204; [10]:200)
19. Tibia, lateral condyle separation from remainder of proximal end in proximal view: bulging from the main surface of the tibia (0); conspicuous narrowing between the body of the condyle and the main body of the tibia (1). ([22]:89; modified from [53]:108)
20. Tibia, medial malleolus, medial expansion: only slightly expanded medially (0); expanded 9% or more of the length of the tibia (1). ([22]:90)
21. Tibia, lateral malleolus, distal extension relative to medial malleolus: even with or extends slightly distally (0); extent beyond the medial malleolus 7% or more of the length of the tibia (1). ([22]:91)
22. Fibula, length relative to femur: greater (0) or less than (1) 70%. ([22]:92)
23. Fibula, proximal end: less than 75% of the proximal width of the tibia (0); 75% or more of the proximal width of the tibia (1). ([12]:592)
24. Fibula, deep groove on the medial side of the proximal end: absent or ridge (0); present but covering less than two-thirds of the width of the fibula (1); present and wide, covering more than two-thirds of the width of the fibula (2). ([12]:593; ordered)
25. Astragalus, height of ascending process relative to tibial length: less than 1/6 (0); between 1/6 and 1/4 (1); greater than 1/4 (2). (wording modified from [22]:93; [21]:135)
26. Astragalus, fibular facet, form: large and facing partially proximally (0); reduced and confined to the anterior half of lateral side of astragalus (1); strongly reduced, facing laterally or absent (2). ([13]:319; modified from [9]:213; [114]:148; ordered)
27. Astragalus, anterior base of ascending process: confluent or only slightly off-set from astragalar body (0); offset from astragalar body by pronounced groove (1). ([13]:322; [9]:216)
28. Astragalus, anteriorly horizontal groove across condyles: absent (0); present (1). ([13]:325; [9]:218).
29. Metatarsal IV, well-developed posteromedial flange on proximal end for articulation with metatarsal III: absent (0); present (1). ([13]:339)
