# Supplementary material for: New Information on the Cranial Anatomy of Acrocanthosaurus atokensis and Its Implications for the Phylogeny of Allosauroidea (Dinosauria: Theropoda)
Source: PLoS One. 2011 Mar 21;6(3):e17932. doi: 10.1371/journal.pone.0017932 (PMC3061882; doi:10.1371/journal.pone.0017932)
Supplement: Appendix S2 — Data matrix. (DOC) [file pone.0017932.s004.doc]

**Appendix S2: Data matrix**

Question marks represent missing data, and gaps are denoted by an underscore symbol.

*Acrocanthosaurus*

0101101011111111111010210111111111111111111?1011011110110111101000001101100011111111110111201001?1201?0100?111102101110112??11011100110111101101111???1?11?11101011111111101????0

*Allosaurus*

100010210210011100000120101110000000000_10000001001000000100101000001111100000000000111010000101?1101101111010012000001111?000011000111001001100111000031111110101111110000111011

*Australovenator*

??????????????????????????????????????????????????????????????????????????????????????1010?00??????????????????????????????????????????00??????10011?????????10100????1010??1????

*Carcharodontosaurus*

?????1?111?111?11111102001111?1111111111111??11111111?1????1110101110001??11?????1?????1?1??1????????11????01110211??????????1?1???????1????????????????1?1?12110??1?????1???????

Coelophysoidea

01102000000???000?0001000?000000??00?00_100?00?00000000?000?100000000000100????0???0001000?10000?0?10?00000?000000?000020?0000?00100000??0000000000000??01000000001?0000000000000

Compsognathidae

0?0020?002????000??0000?0?0????????0?00_1?0??0?000?000???2??1?0???????0011?????????????0?0?0??01????1?01??1?1??100?00000??0?00??00?10??0000000?0??1?0?0?01?10??01??????0001?1????

*Dilong*

10201?200?1???1?0?000111100110?0??01?00_0?0000?10?00000?10???000?0???0??21????????????101?00???1???21?0???1?0???20?????0??????????1??????01?????10??100??021?10??????????????????

*Eocarcharia*

?????1101??1111100???????????????????110111?????01111?????????????????????????????????????????????????0??????????????????????????????????????????????????????????????????????????

*Fukuiraptor*

???????????????????????????????????????????????????????????????????????????????????????0?0??0?????????0????????????0??0??1???0?????????????????????????0?????101??11?1???????????

*Giganotosaurus*

0??1??21????1??111?1102?0211?111?????1??1?10?11111111011010111?1111100?1??11??1111?????1?1?01??????0?1110101100?21?10101?20??1211100?001111111??????000311111211101?111101011????

*Herrerasaurus*

00000000001???0000?000010000000???00000_0?0?00?00000000?00??0000100000000000???????0000000000?00?0?0000000?00000000000010?000000000000?0010100?00001?0000001000000010000000000000

*Lourinhanosaurus*

????????????????????????????????????????????????????????????????????????????????????????????????????????????????0?????????01??0111??110?????????????0??2011??1001?10?01???01?????

*Mapusaurus*

????????0??110?111?110??02??110?1111?111?1???10????????1??1????????????????????????????1?12??0???????11????????????1?1???2??112??1????????101??1??????0?1???1?1110???1?11???1????

*Monolophosaurus*

100021?10?1?????0??101201010000?1?01?00_000000?10010011?010?101010??0?00100?????????1110?0000111?110??00?110?00120?01001?1???00?????????????0????????001000?0????????1???????????

*Neovenator* 1001?00101?011?100?0102000?????????????????????????????????????????????????????????1???0?0000??????0?00?10?1?00020?10011?2??0?2110?01??0?1?0?1???????0130??101110??11?10100?1???1

*Piatnitzkysaurus*

??????????????1????????????????????????????????1??00?0????????0010??00?0100??????????????????????????100??0?0??10?1??0?1110?0?1??????????1????0??????011?101?1????1001???????11??

*Shaochilong*

?????02?0???1??101?1???????????????????????????1011110?00?0?01010??101011??????????????????????????????11??????????????????????????????????????0??1??????????????????????????????

*Siamotyrannus*

???????????????????????????????????????????????????????????????????????????????????????????????????????????????????????????????011??11??????????????0??301110????????????????????

*Sinraptor*

00001010121?000000000121101111001001100_0000?0110010001001001111100000111000110000?11100?0100010?10011011110100120001?1111100011??0???10?10100?0??0?000?0111010001111000000101011

*Tyrannosaurus*

002010010110102?100010000?0110010011110_001?10?3011010011001100010001100?1000000?001001010001001?0021?01?0110000100?0?011???0??00011001??00???0?00?01?0??021?1????1?1?????12?2100

*Tyrannotitan*

????????????????????????????????11111??????????????????????????????????????????????????1?0?01?????????0???011??1??110101?2??00??????0??????0?1??????0?0??1?1?2?1???11????1???????

*Yangchuanosaurus*

?0?01?101?1?????0??1?12?1011100???0?????0?0????????????????????????????????????????1?10????0?2?0??0???0?0??0???1?????????????????????????????????????????????????????????????????
