# Supplementary material for: New Information on the Cranial Anatomy of Acrocanthosaurus atokensis and Its Implications for the Phylogeny of Allosauroidea (Dinosauria: Theropoda)
Source: PLoS One. 2011 Mar 21;6(3):e17932. doi: 10.1371/journal.pone.0017932 (PMC3061882; doi:10.1371/journal.pone.0017932)
Supplement: Appendix S3 — Supplemental references. (DOC) [file pone.0017932.s005.doc]

**Appendix S3: Supplemental references**

142. Sereno PC, Novas FE (1993) The skull and neck of the basal theropod *Herrerasaurus*

*ischigualastensis*. J Vert Paleontol 13: 451-476.

143. Bristowe A, Raath MA (2004) A juvenile coelophysoid skull from the Early

Jurassic of Zimbabwe, and the synonymy of *Coelophysis* and *Syntarsus*.

Palaeontol Afr 40: 31-41.

144. Zhao X-J, Benson RB, Brusatte SL, Currie PJ (2010) The postcranial skeleton of *Monolophosaurus jiangi* (Dinosauria: Theropoda) from the Middle Jurassic of Xinjiang, China, and its systematic relevance. Geol Mag 147: 13-27.

145. Dong ZM (1992) Dinosaurian faunas of China. Bejing: China Ocean Press. 188 p.

146. Gao YH (1992) *Yangchuanosaurus hepingensis*-a new species of carnosaur from

Zigong, Sichuan. Vertebrat PalAsiatic 30: 313-324.

147. Mateus O, Walen A, Antunes MT (2006) The large theropod fauna of the Lourinha Formation (Portugal) and its similarity to that of the Morrison Formation, with a description of a new species of *Allosaurus*. N M Mus Nat Hist Sci Bull 36: 123-129.

148. Buffetaut E, Suteethorn V, Tong H (1996) The earliest known tyrannosaur from

the Lower Cretaceous of Thailand. Nature 381: 689-691.

149. Stromer E (1934) Ergebnisse der Forschungsreisen Prof E. Stromers in den Wüsten

Ägyptens. II. Wirbeltierreste der Baharje-Stufe (unterstes Cenoman). 13. Dinosauria von E. Stromer. Abhandlungen der Bayerischen Akademie der Wissenschaften Mathematisch-Naturwissenschaftliche Abteilung, Neue Folge 22: 1-79.

150. Rauhut OWM (1995) Zur systematischen Stellung der afrikanischen Theropoden

*Carcharodontosaurus* Stromer 1931 und *Barhariasaurus* Stromer 1934. Berliner

Geowissenschaftliche Abhandlungen E 16: 357-375.

151. Ostrom JH (1978) The osteology of *Compsognathus longipes* Wagner. Zitteliana 4:

73-118.

152. Peyer K (2006) A reconsideration of *Compsognathus* from the Upper Tithonian of Canjuers, Southeastern France. J Vert Paleontol 26: 879–896.

153. Ji Q, Ji S (1996) [On the discovery of the earliest fossil bird in China (*Sinosauropteryx* gen. nov.) and the origin of birds] (In Chinese). Geol China 233: 30-33.

154. Currie PJ, Chen P-J (2001) Anatomy of *Sinosauropteryx prima* from Liaoning,

northeastern China. Can J Earth Sci 38: 1705-1727.

155. Hwang SH, Norell MA, Ji Q, Gao K (2004) A large compsognathid from the

Early Cretaceous Yixian Formation of China. J Syst Palaeontol 2: 13-30.
